# China’s rising hydropower demand challenges water sector

**DOI:** 10.1038/srep11446

**Published:** 2015-07-09

**Authors:** Junguo Liu, Dandan Zhao, P. W. Gerbens-Leenes, Dabo Guan

**Affiliations:** 1 School of Nature Conservation, Beijing Forestry University, Qinghua East Road 35, Haidian District, 100083, Beijing, China; 2 Department of Water Engineering and Management, University of Twente, P.O. Box 217, 7500 AE, Enschede, The Netherlands; 3 Water Security Research Centre, School of International Development, University of East Anglia, Norwich NR4 7TJ, United Kingdom

## Abstract

Demand for hydropower is increasing, yet the water footprints (WFs) of reservoirs and hydropower, and their contributions to water scarcity, are poorly understood. Here, we calculate reservoir WFs (freshwater that evaporates from reservoirs) and hydropower WFs (the WF of hydroelectricity) in China based on data from 875 representative reservoirs (209 with power plants). In 2010, the reservoir WF totaled 27.9 × 10^9^ m^3^ (Gm^3^), or 22% of China’s total water consumption. Ignoring the reservoir WF seriously underestimates human water appropriation. The reservoir WF associated with industrial, domestic and agricultural WFs caused water scarcity in 6 of the 10 major Chinese river basins from 2 to 12 months annually. The hydropower WF was 6.6 Gm^3^ yr^−1^ or 3.6 m^3^ of water to produce a GJ (10^9^ J) of electricity. Hydropower is a water intensive energy carrier. As a response to global climate change, the Chinese government has promoted a further increase in hydropower energy by 70% by 2020 compared to 2012. This energy policy imposes pressure on available freshwater resources and increases water scarcity. The water-energy nexus requires strategic and coordinated implementations of hydropower development among geographical regions, as well as trade-off analysis between rising energy demand and water use sustainability.

Energy and water resources are an important nexus recognized in academic and policy debates^1^,^2^,^3^. The Organization for Economic Co-operation and Development (OECD) and the International Energy Agency (IEA) state that the availability of an adequate water supply is an increasingly important criterion for assessing the physical, economic and environmental viability of energy projects^4^. Water could therefore become a serious issue for power generation projects^4^. Today, fossil fuels are the dominant energy carriers; they supply 80% of the world’s energy use^5^. The growing demand for energy, especially in rapidly developing countries such as China^6^, Brazil^7^ and India^8^, has stimulated an expansion of renewable energy. Hydropower is often a key component in achieving renewable energy targets as part of climate change policies, e.g. in China. However, recent studies have revealed that hydroelectric reservoirs are an important source of greenhouse gases, partly offsetting the continental carbon sink^9^,^10^. There is also an increasing concern about the water sustainability of hydropower^11^,^12^,^13^,^14^,^15^, leading to the need of an in-depth study on energy-water nexus of hydropower.

Conventional policy development tends to occur in “silos”, reducing the ability to consider potential trade-offs and synergies among sectors^3^. The water footprint (WF) concept quantifies the freshwater consumed in the production or consumption of a commodity, good, or service^16^ and is a powerful and increasingly popular tool to study the water and energy nexus^16^. The WF quantifies water consumption of different components of freshwater: green water (soil water), blue water (surface and groundwater), and grey water (polluted water). Water consumption refers to the volume of freshwater used and then evaporated or incorporated into a product^16^. It is important to distinguish the term “water consumption” from the term “water utilization”. A part of “water utilization” is water consumption, while the rest either returns to the catchment where water was withdrawn or flows to another catchment or to the sea. As of 2013, few researchers had attempted to quantify the WF of hydropower production^11^,^12^,^13^,^14^,^15^. There has been no spatially explicit analysis of reservoir WFs by considering a large number of reservoirs in different locations, leading to speculation about how reservoirs influence water consumption and water scarcity. Moreover, when a reservoir provides more than hydropower (e.g. flood control, irrigation and navigation), its WF should be allocated among the different purposes. Most studies have attributed a hydroelectric reservoir’s water consumption entirely to power generation, thereby overestimating the hydroelectric WF^12^,^13^,^14^,^15^,^17^.

Here, we analyzed the reservoir WFs in China by determining the volume of freshwater that evaporates from reservoirs and the hydroelectric WF of reservoirs that generate power. In this analysis, reservoir evaporation only includes *blue* water, as we do not consider soil water or water pollution. We investigated the spatial distribution of reservoir WFs, how it affects water scarcity at a river basin level and the spatial distribution of the hydroelectric WF.

China has a strong political emphasis on future hydroelectric development^18^. China possesses the world’s largest number of dams (about half of the world’s total) and generated 864 × 10^9^ kWh of hydropower in 2012 (~20% of the world’s total)^19^. In 2007, China’s *Medium- and Long-term Plan for Renewable Energy Development* proposed developing a gross installed hydropower capacity of 300 GW by 2020 — more than double the 2007 capacity^6^. Following the publication of China’s 12^th^ five-year plan (2011–2015), the state council set a goal for non-fossil fuel energy to account for 15% of the total energy consumption by 2020, with more than half coming from hydropower^20^. The target of installed hydropower capacity was re-set to 420 million kW by 2020^20^, which is a 70% increase in comparison to that in 2012^20^. However, the water requirement for food production will also increase due to socioeconomic development and dietary shifts towards water-intensive foods such as meat^21^,^22^, putting additional pressure on China’s water resources. To meet these goals, it is necessary to understand the spatial distribution of the reservoir and hydropower WFs; however, until the present study, no such assessments are found.

## Results

### Reservoir WF

The Chinese reservoir WF totaled 27.9 × 10^9^ m^3^ (Gm^3^) in 2010, with values ranging from 0.7 Gm^3^ for the Northwest rivers basin to 8.0 Gm^3^ for the Yangtze River basin (Table 1). The reservoir WF accounted for 22% of the total *blue* water WF of China (Tables S1, S2); this proportion ranged from 5% for the Northwest rivers basin to 57% for the Southeast rivers basin (Table S2). Hence, neglecting reservoir WF seriously underestimates the *blue* water WF.

**Figure 1:**
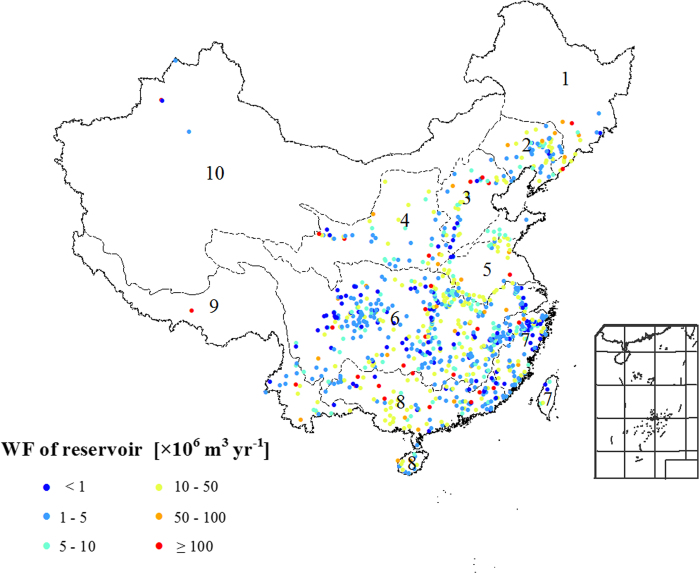
The water footprint (WF) of 875 representative reservoirs for China’s 10 river basins. The 10 river basins include the Songhuajiang (1), Liaohe (2), Haihe (3), Yellow river (4), Huaihe (5), Yangtze river (6), Southeast rivers (7), Zhujiang (8), Southwest rivers(9), and Northwest rivers (10). [*Created with ArcGIS 9.3.1*]

The reservoir WFs varied widely among the basins (Fig. 1) and months (Table 1). The Yangtze and Zhujiang river basins have the highest reservoir WF; they account for 46% of the total national reservoir WF. One explanation is that about 45,200 dams have been built throughout the Yangtze River basin, which amounts to about 51% of the Chinese total^20^. The Southwest and Northwest river basins have the lowest reservoir WF; they account for about 6% of the national total. In the Northwest river basin, precipitation is low and the climate and topography are unsuitable for the construction of large dams. The WF from May to September accounts for more than half of the annual WF in all basins, except in the Southwest rivers basin (Table 1). The Huang, Huai, Hai, and Yangtze river basins have large reservoirs (WF > 10 × 10^6^ m^3^) that coexist with small reservoirs (WF < 10 × 10^6^ m^3^), whereas large reservoirs dominate the Southeast rivers basin.

### Water scarcity

**Figure 2:**
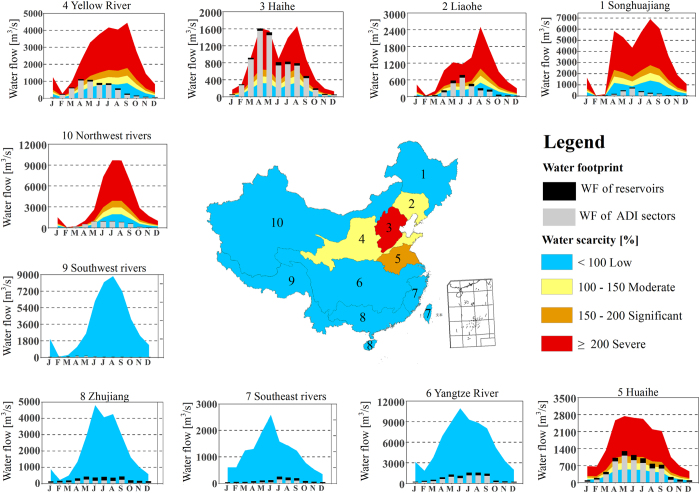
Annual and monthly water scarcity assessment for the 10 Chinese river basins. ADI represents the agricultural, domestic and industrial sector (i.e., excludes the reservoir WF). Note that the y-axis scale differs from graph to graph. Different color tones indicate different levels of water scarcity: red for severe, orange for significant, yellow for moderate and blue for low. In the graph, for each river basin, the color tones associated with the bars indicate the same severity of water scarcity. For example, when the blue WF (the grey and black bars) reaches a red zone, severe water scarcity occurs in that month for the basin. [*Created with ArcGIS 9.3.1*]

When the reservoir WF is not considered, only three river basins suffered from a moderate to severe scarcity on an annual basis: the Haihe (371%), Huaihe (154%), and Liaohe (102%) basins. The severity of the water scarcity equaled the ratio of the WF to the water availability (see Method). But when the reservoir WF is taken into account, four river basins suffered from a moderate to severe annual water scarcity: the Haihe (378%), Huaihe (182%), Liaohe (127%), and Huanghe (hereafter, Yellow River: 104%) basins (Fig. 2). Thus, water scarcity is significantly underestimated when the reservoir WF is not considered.

We found a moderate to severe water scarcity in six river basins for at least two months per year: for the Haihe (12 months), Huaihe (10 months), Liaohe (6 months), Yellow river (6 months), Northwest rivers (4 months) and for the Songhuajiang (2 months) basins (Fig. 2; Tables S3 and S4). Although the Liaohe and Northwest rivers basins do not show a moderate to severe annual water scarcity, they still suffer from a seasonal water scarcity. Previous studies often assessed water scarcity on an annual basis^23^,^24^, but monthly assessments can reveal critical seasons when measures should be taken to mitigate or adapt to water scarcity, particularly when the needs of hydroelectricity and other sectors must be balanced.

### Hydroelectric WF

**Figure 3:**
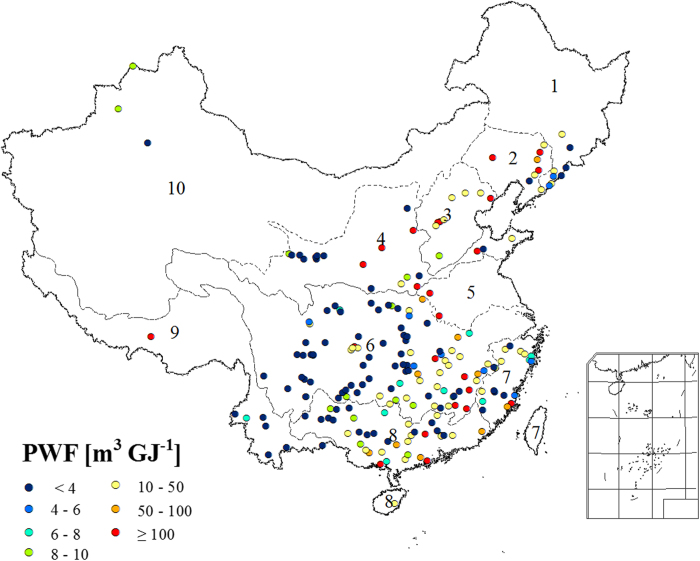
The product water footprint (PWF) of hydropower (WF_h_) for 209 representative power plants in China. [*Created with ArcGIS 9.3.1*]

China’s hydroelectric WF totaled 6.6 Gm^3^ yr^−1^ in 2010. This was about 24% of the reservoir WF. We also calculated the product water footprint (PWF; the WF per unit of goods or services, including hydropower generation^16^). PWF varied widely among the plants (Fig. 3). The largest hydropower plant, the Three Gorges Dam on the Yangtze River, had a product WF of 2.0 m^3^ GJ^−1^; the second-largest plant, the Gezhouba Dam on the Yangtze River, had a PWF of only 0.6 m^3^ GJ^−1^. There is a general trend of relative lower PWF at upstream and larger PWF at downstream within a river basin, e.g. for the Yangtze and Zhujiang river basins (Fig. 3). Table S5 summarizes the hydroelectric PWF for the 209 power plants.

By weighing the WF of the representative hydropower plants by their power generation, we calculated a weighted average hydroelectric PWF of 3.6 m^3^ GJ^−1^. Hydroelectric PWF varied widely, from 0.001 m^3^ GJ^−1^ for the Hongyi plant to 4234 m^3^ GJ^−1^ for the Zhanggang plant, both in the Yangtze river basin. This range is much wider than previous IPCC estimates of between 0.011 and 58 m^3^ GJ^−1^ (or 0.04 to 209 m^3^ MWh^−1^)^11^. The IPCC summarized the range based on two data points from four literature sources^17^. Comparatively speaking, our results are more solid and specific by including 209 officially surveyed hydropower plants in China.

## Discussion

The reservoir WF is seldom accounted for when assessing the human appropriation of water resources or the pros and cons of constructing reservoirs to provide hydroelectricity. We provided a spatially explicit assessment of the reservoir and hydropower WFs by considering 875 representative reservoirs and 209 hydropower plants in different locations, and demonstrated that accounting for these WFs is an important consideration when evaluating the environmental sustainability of a reservoir or of hydropower as an energy source. We therefore recommend that the WF should be assessed in any sustainability evaluation for new reservoirs, as well as in the evaluation of existing reservoirs, so that the consequences of the WF on downstream environmental flows and other water users can be evaluated. About one-fourth of the world’s reservoirs with a dam higher than 15 m provide multiple services, and more than 40% of the 8689 reservoirs that provide hydropower are also used for other purposes^17^. For multi-purpose reservoirs, it is more logical to “share the burden of water consumption” among the different beneficiaries^17^, as we have done in the present study.

The PWF of 3.6 m^3^ GJ^−1^ calculated here is lower than several previously reported hydroelectric PWFs: e.g. 68 m^3^ GJ^−1^ by Mekonnen and Hoekstra^13^ and 22 m^3^ GJ^−1^ by Gerbens-Leenes *et al*.^12^ for the global average, 6.1 m^3^ GJ^−1^ for New Zealand^15^ and 19 m^3^ GJ^−1^ for the United States^25^ (Table 2). Although the reservoirs of many power plants are also used for other purposes (e.g., irrigation supply), the WF was attributed only to hydropower in all previous studies except one^26^. Many of these studies were based on a limited number of power plants, and may therefore contain large errors in the hydroelectric WF. Mekonnen and Hoekstra^13^ considered only 35 of the world’s 8689 plants^17^, which accounted for 8% of global electricity generation^17^. Moreover, the selected plants were not broadly representative, and included only two Chinese plants and no plants in the United States. In addition, Gerbens-Leenes *et al*.^12^ and Mekonnen and Hoekstra^13^ did not use recent data on electricity generation. Those studies are pioneer work but results are insufficiently representative. We allocated the total WF among the different outputs of the reservoirs, and to the best of our knowledge, we are the first to obtain results and analyze the variances based on a large number of reservoirs (i.e. 875 for China) and hydropower plants (i.e. 209 for China) and to demonstrate the spatial distribution of WF of reservoirs and hydropower.

The Chinese national average hydroelectric PWF of 3.6 m^3^ GJ^−1^ is higher than that of most other technologies, which typically have maximum values of 1.1 to 1.4 m^3^ GJ^−1^ (4 to 5 m^3^ MWh^−1^)^27^. At present, the most important primary energy carriers include crude oil, coal, natural gas, uranium, hydroelectric power, solar and wind energy^28^. The PWF of wind energy and underground uranium mining is negligible^29^. The water footprint of electricity from solar energy, coal-fired and nuclear thermal energy is generally far below 1.0 m^3^ GJ^−1^^14^,^29^ (Table 3). Thus, hydropower is not an efficient solution to energy supply from a water consumption perspective. As a large water consumer, the hydropower sector has triggered great concern^27^ since it may result in reputational and financial risks, in particularly in regions where freshwater is scarce.

**Figure 4:**
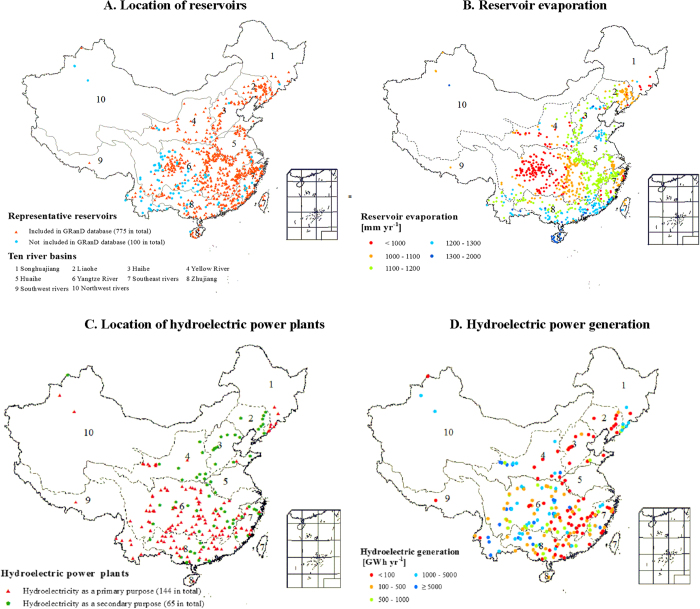
Locations of representative reservoirs, corresponding reservoir evaporation, the location of representative hydroelectric power plants and their power generation. [*Created with ArcGIS 9.3.1*]

China covers a large geographic area, with a wide and complex variation in climatic conditions, thus we expect no correlation between latitude and WF; our results (Fig. S1) confirm this hypothesis. Evaporation is a key parameter in influencing the WF. Evaporation varies from 748 mm yr^−1^ for the Ankang reservoir (Yangtze River basin) to 1646 mm yr^−1^ for the Yantan reservoir (Zhujiang River basin) (Fig. 4B). Most reservoirs had an evaporation rate between 900 and 1500 mm yr^−1^, but tropical reservoirs generally had higher evaporation rates than temperate-zone reservoirs (Fig. 4B). However, different evaporation rates cannot explain the large variation in PWF.

**Figure 5:**
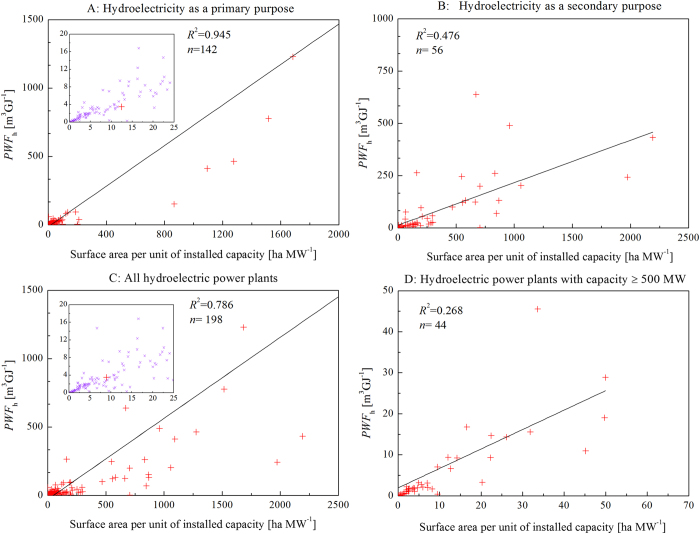
Relationship between the PWF of hydroelectric power (*PWF*_*h*_) and the flooded area per unit of installed hydroelectric capacity (α) for (**A**) plants with power generation as the primary purpose; (**B**) plants with power generation as the secondary purpose; (**C**) all plants; and (**D**) hydroelectric power plants with an installed hydroelectric capacity greater than 500 MW.

We found that the large variation of the PWF was mainly determined by the reservoir area per unit of installed hydroelectric capacity (α). The value of α averaged 9.9 ha MW^−1^, with a minimum of 0.004 ha MW^−1^ (Dayingjiang plant, Southwest rivers basin) and a maximum of 5280 ha MW^−1^ (Zhanggang plant, Yangtze River basin). Plants with a relatively large α generally had a larger PWF than those with a small α (Fig. 5). We found a linear relationship between PWF and α (Fig. 5). This linear relationship was much stronger for plants with hydropower as their main purpose than for those with power as their secondary purpose. Plants with a high installed hydroelectric capacity also had a strong linear relationship with PWF (Fig. 5), probably because they were often constructed primarily to provide hydropower. Hence, the reservoir surface area also plays an important role in the size of the hydropower WF^13^,^17^. To our best knowledge, very few studies reported on the relationship between the PWF of electricity, the reservoir area and the installed hydroelectric capacity except for the study of Mekonnen and Hoekstra^13^. The large reservoir area is not exclusively due to the need for power production, as the reservoir may be used for multiple purposes (e.g., irrigation supply or flood control), or originates from a natural lake before there was a reservoir^17^.

The generally lower PWF upstream of the major river basins can largely be explained by the close relation between the PWF and α. In China, several large rivers, such as the Yangtze River, originate from high-altitude western regions. The upstream areas are often characterized by steep mountains, while downstream areas are dominated by flat plains. These topographical differences make upstream areas more suitable to construct relatively small-area reservoirs.

Water scarcity depends on many factors, such as blue water consumption, water management and precipitation . In this paper, water consumption is reflected by the concept of the water footprint, which refers to water consumption in different sectors. Water resources come directly or indirectly from precipitation. Water management is a factor that can influence water scarcity, but management is also included in the estimation of water footprint. For example, irrigated agriculture differs from rainfed agriculture, generating a blue WF. The sustainability of monthly *blue* water consumption is assessed by comparing *blue* WF with blue water availability (the difference between blue water resources and environmental flow requirements). The general idea of the monthly *blue* water scarcity assessment is to judge whether the human induced *blue* WF is beyond or below blue water availability in a month, and such an assessment approach is recommended by the Water Footprint Network^16^.

To decrease the human impact on climate change, the Chinese government promotes the use of renewable energy, in particular hydropower^20^. The reservoir WF accounts for over one-fifth of the total human water appropriation in China. A 70% increase in installed hydropower capacity planned in the national energy policy^20^ will further increase the hydropower WF, as well as the reservoir WF, unavoidably resulting in more competitive water consumption between energy supply and other purposes, such as water for food production. There may be large trade-offs between food security and energy security in China given its already serious water scarcity. The reservoir WF may reduce the availability of water for food production, but at the same time, the regulating role of reservoirs may benefit food production. After a reservoir is constructed, it can restore flood. As a result, food production can benefit from reduced flood damage to agricultural fields and from increased availability of irrigation water in the reservoir. The complex relation makes the trade-off assessment more difficult, although such an assessment is beyond the scope of the current paper.

China’s hydropower resources are mainly concentrated in the western regions^30^, where the PWF is generally low; but energy demand is dominant in the populous eastern regions with a generally high PWF. From a water conservation point of view, eastern China should not further expand its capacity in hydropower. Future reservoir construction in eastern China should avoid aiming at hydroelectric production. Hydropower capacities can be increased strategically and coordinated by the national government, with mutual agreement with provincial governments. On the other hand, many ongoing hydropower projects are located in the ecologically important but vulnerable western regions^30^,^31^, which are sources of many large rivers, including the Yangtze River and the Mekong River that is shared by six countries. There is a need for strategic and coordinated implementation of hydropower development among different geographical regions. China’s future hydropower development must take into account nature conservation, biodiversity protection^30^, as well as ecological resilience locally and for downstream regions^31^. Furthermore, hydropower development should take the impacts of hydropower on water use sustainability and global climate change into account in addition to the raising energy demand.

Our study has several limitations. First, reservoirs also have beneficial impacts. They store water for agriculture during dry months, thereby alleviating drought and increasing food production. Moreover, they reduce the risks of devastating floods in downstream regions^19^. With proper management, reservoirs can increase water availability during dry seasons and prevent floods in wet seasons. Unfortunately, these benefits cannot be handled by the present simplistic analytical approach. A broader conceptual framework that accounts for trade-offs among different water uses is still needed, but is beyond the scope of our study.

Second, we calculated the reservoir WF by accounting for the total evaporation. It is reasonable to note that before the reservoir was created, there was also evaporation from the area^27^. However, evaporation from the original flowing river is likely to be considerably less than that from the reservoir, since the reservoir area is generally much larger than the river’s original area^27^. We therefore argue that the full reservoir evaporation (without subtracting evaporation from the original rivers) should be considered when quantifying the water consumption that can be associated with a specific human purpose, such as electricity generation. In addition, after a reservoir is constructed, precipitation can be completely turned into runoff over the reservoir surface; hence, more blue water is available. However, it remains a shortcoming for this study that cannot quantify the change of blue water resources caused by the reservoir construction. Furthermore, we only included the functional reservoir evaporation, and excluded the supply-chain WF of hydroelectric generation; that is, we excluded the WF of the materials used in the construction, operation, and maintenance of the site, although this is negligible compared to the functional WF^27^,^32^.

Third, the water that evaporates from a reservoir can be considered “lost” because, unless it falls as precipitation close to its source, it cannot be reused in the same catchment. However, some of this water may be recaptured within the same catchment, particularly in areas with steep mountains that create orographic precipitation. Reservoirs also have a temporally dynamic water surface area. As a result of annual and seasonal fluctuations in the water volume, the difference between the minimum and maximum reservoir area will also vary. Reported areas generally refer to the maximum, and this probably leads to some overestimation of annual evaporation.

Finally, using economic values to allocate water consumption to hydroelectric power generation does not tell the entire story, since the “social value” of water (e.g., for irrigation or recreation) might be higher than its economic price. In addition, there is an omission of including opportunity costs in the methodology. Consumption of different water users is inevitable and one has to ensure that consumption takes place in those sectors where there is more economic value per unit of water. Hydropower is the lowest cost energy option and its generation provides extensive benefits to major economic benefits and contributes to growth and improvements in social well being. Thus, although our study provides a strong first attempt to demonstrate the importance of reservoir and hydroelectric WFs, it will be necessary to develop a more sophisticated methodology for estimating water consumption from multi-purpose reservoirs to obtain better estimates that can be used to support reservoir management^27^.

## Methods

### Reservoir WF

The WF of a product or service is defined as the volume of freshwater used to produce that good at the place where it was actually produced^16^. A WF consists of three components: the green WF, the blue WF, and the grey WF^16^. The green WF refers to soil water from precipitation that evaporates during the production of a good or service. The blue WF refers to surface and groundwater consumed during the production of a good or service. The grey WF is related to water pollution and is defined as the amount of water needed to dilute pollutants discharged into natural water systems to the extent that the quality of the ambient water remains better than the required water quality standards^16^. Based on these definitions, hydroelectric power only generates a blue WF.

We selected 875 representative reservoirs in China’s 10 river basins (Fig. 5A): the Songhuajiang, Liaohe, Haihe, Huanghe (Yellow River), Huaihe, Changjiang (Yangtze River), Southeast rivers, Zhujiang, Southwest rivers, and Northwest rivers basins. These representative reservoirs accounted for 76% of the total reservoir volume in China. There are two main criteria to select these reservoirs: (1) A good coverage of large- and medium-sized reservoirs; and (2) data availability. Among these reservoirs, 775 are included in the GRanD database^33^, which contains information on the location, main use, dam height, storage capacity, surface area and the name of the basin where the dam is located. We selected the other 100 reservoirs as follows: there are 185 large and medium-sized hydroelectric power plants in China^34^, of which 55 plants are included in the GRanD database^33^. Among the remaining 130 plants, data on either the reservoir volume or height are available for 100 plants (see the data sources in SI Appendix I). We calculated the reservoir area based on one of the following two types of regression functions^33^: when both the reservoir volume and height were available, we used a regression function that related the reservoir area to these two variables; when only the reservoir volume was available, we used another regression function that related the reservoir area to the reservoir volume^33^. Table S1 provides the data for the 875 reservoirs.

We first calculated the annual WF of a representative reservoir i in river basin j, *F* (m^3^ yr^−1^), which was equivalent to the total reservoir evaporation on an annual basis, by multiplying the annual water evaporation by the surface area:




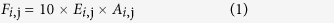




where, *E* (mm yr^−1^) is the water evaporation and *A* (ha) is the surface area of reservoir i in river basin j. Since 1 mm is equal to 10 m^3^ ha^−1^, the factor 10 is used to convert mm into m^3^ ha^−1^.

We used the measured reservoir evaporation from literature for 69 reservoirs (Tables S6 and S7) to estimate the WF of these reservoirs. These reservoirs accounted for 62% of the total power generation and 40% of the total storage volume of the 875 reservoirs. When measured data were unavailable, we calculated the annual evaporation *E* (mm yr^−1^) from the surface area of the reservoir by multiplying the reference evaporation^35^,^36^ with a coefficient^35^. Data on reference evaporation were obtained from the International Institute for Applied Systems Analysis (IIASA)^36^, which provides information for each month and on an annual basis for global land areas, excluding Antarctica, from 1961 to 1990 with a spatial resolution of 30 arc-minutes. In most cases, the hydroelectric power reservoir was smaller than the size of a grid cell in the IIASA dataset. For the reservoirs that appeared in more than one grid cell, we used the arithmetic average value of the cells. The coefficient shows the ratio of surface water evaporation to reference evaporation, and a value of 1.05 is used^35^.

To check the accuracy of the simulated reservoir evaporation (Fig. 5B) based on the reference evaporation from IIASA^36^ and the coefficient^35^, we compared the calculated evaporation with the measured evaporation for the 69 reservoirs for which measured data were available. We defined the relative error (*e*) as the difference between the estimated and measured evaporation divided by the measured value:




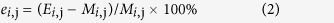




where *e*, *E*, and *M* are the relative error (%), estimated evaporation (mm yr^−1^), and measured evaporation (mm yr^−1^) for representative reservoir i in river basin j.

There are many reasons that can lead to differences between measured and simulated evaporation. These reasons include the imperfectness of simulation models, and measurement errors, among others. In this study, we found that for 90% of the reservoirs (62 out of 69), the relative error was within 30% of the measured reservoir evaporation, and for half of the reservoirs, the relative error was within 15% of the measured reservoir evaporation (see Table S7). This indicates a good estimation of the reservoir evaporation. Hence, for the reservoirs where data on measured evaporation were not available, we used the simulated evaporation.

### Reservoir WF at river basin and national levels

The reservoir WF for a river basin was calculated by first summing up the reservoir WF of all representative reservoirs and then by dividing this sum by the proportion of the total reservoir area in the river basin that was accounted for by the area of the representative reservoirs:




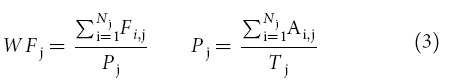




where *WF* is the total reservoir *WF* (m^3^ yr^−1^) of a river basin j, *N* is the number of representative reservoirs in river basin j, *P* is the proportion of the total reservoir area in the river basin j that was accounted for by the area of the representative reservoirs in the same river basin, *T* is the total reservoir area (ha) in a river basin j. *F* and A have the same meaning and units as those in Eq. 1.

China’s total reservoir WF was estimated by summing up the reservoir WFs of the 10 river basins:




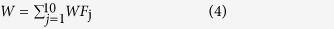




where *W* is the total reservoir *WF* (m^3^ yr^−1^) in China and 10 is the number of total river basins in China.

### Hydroelectric WF

We estimated the hydroelectric WF for 209 hydropower plants (Fig. 5C,D), which generate 53% of China’s total hydroelectricity. From the Global Reservoir and Dam (GRanD) database^33^, we first selected 160 hydroelectric power plants for which hydroelectricity is the primary purpose (95) or the secondary purpose (65). For the additional 100 reservoirs (see the previous section for details), we identified 49 plants that had the production of hydroelectricity as their primary purpose. Data on the location, dam height, reservoir surface area, reservoir capacity, and electricity generation were obtained from the GRanD database^33^ (see Appendix I and Appendix II).

For plants with multiple functions, we allocated the total WF among the different functions according to their economic values using an allocation coefficient η. The annual hydroelectric power WF of a specific reservoir is calculated as follows:




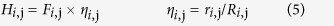




where *H* is the hydroelectric power WF (m^3^ yr^−1^) of reservoir i in river basin j, η is the allocation coefficient, defined as the ratio of the annual revenue generated from hydroelectric power (*r*, RMB yr^−1^) to the total annual revenue (*R*, RMB yr^−1^) generated by the hydroelectric power plant. *r* is calculated by multiplying the hydroelectric power that was generated (kWh yr^−1^) by the electricity price (RMB kwh^−1^). Data on electricity prices were obtained from the State Electricity Regulatory Commission^37^. We derived additional data on economic revenues from literature (see Table S7). Economic data were available for 26 hydroelectric power plants (including 13 with hydroelectric power as their main purpose), which were responsible for generating half of the hydroelectric power from the 209 representative hydroelectric power plants. We calculated η for 26 power plants that produce half of the hydroelectricity from the 209 representative plants (Table S8). For hydroelectric power plants for which no economic data were available, we used the weighted average values of η for the two categories of plants (i.e., those with hydroelectricity as their primary or secondary purpose), which were calculated based on the values for the 26 hydroelectric power plants. Here we defined two categories of hydropower plants: plants with electricity generation as their main purpose (e.g., the Three Gorges Dam in the Yangtze river, with annual electricity generation of 84.37 × 10^3^ GWh) and plants with electricity generation as a secondary purpose (e.g., the Xiaolangdi Dam in the Yellow river, with annual electricity generation of 5.196 × 10^3^ GWh). For reservoirs with electricity generation as the main purpose, the weighted average η was 0.48 (Table S8). For reservoirs with electricity generation as the secondary purpose, the weighted average η was 0.15. For the remaining 183 power plants, we use the corresponding weighted average allocation coefficients.

Following Hoekstra *et al*.^16^, we calculated the product WF (*f*) of hydroelectric power, which equaled the total annual amount of water that evaporated from a reservoir that is used primarily or secondarily to generate hydroelectric power, expressed per unit of electricity generated (m^3^ GJ^−1^). *f* was calculated by dividing the annual hydroelectric power water footprint *H* of a reservoir by the annual amount of electricity generated (*G*; GJ yr^−1^):




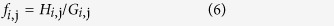




For each basin, we calculated the proportion of the total hydropower accounted for by the representative power stations. We used this proportion, combined with the WF of the representative hydroelectric power reservoirs in the river basin, to calculate the total hydroelectric WF for the river basin. The total hydroelectric WF in China was calculated by summing up the total hydroelectric WF of the 10 major river basins:




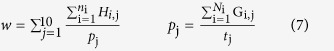




where *w* is the total hydroelectric WF (m^3^ yr^−1^) in China, 10 is the number of total river basins in China, *p* is the proportion of the total hydropower accounted for by the representative power stations in river basin j, *t* is the annual total amount of electricity generated (GJ yr^−1^) in a river basin j.

### Annual and monthly water scarcity

We quantified the annual and monthly water scarcity for the river basins with and without considering the reservoir WF. The severity of the water scarcity equaled the ratio of the WF to the water availability, which represented the difference between the natural runoff and the environmental flow requirements^38^:




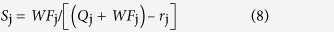




where *S* is the water scarcity indicator of river basin j, *WF* is the total blue water WF, *Q* is the actual runoff in the river basin, and the sum of *Q* and *WF* equals the natural flow^7^. *r* is the environmental flow requirements. *WF*, *Q* and *r* have a unit of m^3^·month^−1^ (for monthly water scarcity calculation) or m^3^·yr^−1^(for annual water scarcity calculation).

In the above equation, we follow Hoekstra *et al*.^38^ and define water scarcity as the ratio of blue water footprint to the blue water availability. The water footprint reflects human’s water appropriation and is related to human water demand, while water availability accounts for environmental water demands by subtracting the presumed flow requirement for ecological health from the total natural runoff. Here, we follow Hoekstra *et al*.^38^ and assume that 80% of the natural runoff should be maintained for presumed environmental flow requirements. This 80 per cent rule is proposed by water resource experts as a general precautionary guideline^38^. This means that the presumptive standard could be met as long as the human appropriation of water resources remains below 20% of the natural flow^38^.

The annual and monthly data on blue water WFs for the agricultural, industrial, and domestic sectors are available at a 30 arc-min resolution from 1996 to 2005^38^. The annual and monthly data on actual runoff at a 30 by 30 arc-min resolution were obtained from the Composite Runoff V1.0 database^39^. The annual and monthly blue water WFs of the three sectors and the actual runoff at the river basin level were calculated by aggregating the high-resolution data within a river basin. Previous calculations only considered the WF of the agriculture, industry, and domestic sectors^38^, and ignored hydroelectric power and reservoir evaporation. In our assessment of water scarcity, we calculated the results for two situations: including and excluding the reservoir WF in the total blue WF (*WF*) in a river basin. To calculate the monthly reservoir WF, we allocated the annual WF to each month based on the ratio of evaporation in that month to annual evaporation. These ratios were calculated using the monthly reservoir evaporation data^36^.

Water scarcity *S* for each basin is classified into four categories^38^: low blue water scarcity (<100%), in which the blue water WF is less than 20% of the natural flow and does not exceed the blue water availability, river runoff is unmodified or only slightly modified, and environmental flow requirements are met; moderate blue water scarcity (100% to 150%), in which the blue water WF is between 20% and 30% of natural flow, runoff is moderately modified, and environmental flow requirements are not met; significant blue water scarcity (150% to 200%), in which the blue water WF is between 30% and 40% of natural flow, runoff is significantly modified, and environmental flow requirements are not met; and severe water scarcity (>200%), in which the monthly blue water WF exceeds 40% of natural runoff, runoff is seriously modified, and environmental flow requirements are not met.

## Additional Information

**How to cite this article**: Liu, J. *et al*. China’s rising hydropower demand challenges water sector. *Sci. Rep*. **5**, 11446; doi: 10.1038/srep11446 (2015).

## Supplementary Material

Supplementary Information

## Figures and Tables

**Table 1:** Water footprint (WF) of the reservoirs in China’s 10 river basins.

Basin no.	Basin name	Reservoir WF (×10^6^ m^3^)												
		Jan.	Feb.	Mar.	Apr.	May	June	July	Aug.	Sept.	Oct.	Nov.	Dec.	Annual
1	Songhuajiang	40	55	100	190	256	249	223	209	184	132	77	47	1763
2	Liaohe	40	56	107	195	266	265	233	219	194	135	76	47	1835
3	Haihe	39	52	87	141	182	196	165	148	127	93	61	42	1332
4	Yellow River	67	91	143	214	267	299	277	255	195	141	98	71	2118
5	Huaihe	147	179	267	386	492	556	522	509	412	313	222	164	4169
6	Yangtze River	301	355	496	684	835	932	1101	1078	832	605	436	336	7991
7	Southeast rivers	91	97	122	163	191	214	279	278	220	173	132	103	2064
8	Zhujiang	252	259	319	401	479	509	593	553	502	426	337	276	4905
9	Southwest rivers	59	78	100	116	116	102	98	97	90	78	65	57	1057
10	Northwest rivers	8	13	29	66	94	109	111	101	76	43	19	9	679
	China	1044	1234	1771	2554	3178	3430	3602	3450	2834	2139	1524	1152	27912

Locations of the basins and their water footprints are shown in Figure 1.

**Table 2:** Comparison of hydroelectric product water footprints (PWF) estimated in the present study with previous values of Bakken *et al*.^17^

Study area	Hydroelectric PWF [m^3^ GJ^−1^]	Hydroelectric PWF [m^3^ MWh^−1^]
United States average^40^	4.7	17
United States average −120 largest plants^25^	19	68
Arizona, United States^26^	31.6	113.9
California, United States^41^,^42^	Min: 0.01 Median: 1.5 Max.: 58	Min: 0.04 Median: 5.4 Max.: 209
California^29^	Mean: 1.5	Mean: 5.4
	Median: 7.2	Median: 26
“All plants” in Northern New Zealand^7^	6.1	21.8
Norway^43^	1–1.2	3.8–4.4
Ethiopia Omo-Ghibe River^44^	Min.: 9.4	Min.: 34
	Max: 22.7	Max: 82
Ethiopia (Blue Nile)^45^	Min: 3.1	Min: 11
	Mean: 27.5	
	Max.: 38	
Sudan Roseires and Sennar irrigation reservoirs^46^	Min.: 381 Mean: 411 Max.: 978	Min.: 1371 Max.: 3521
Austria, Ethiopia, Turkey, Ghana, Egypt and PDR Laos^46^	Max.:1736	Max.: 6250
Global average^4^	22	80
Worldwide, 35 plants^5^	Min.: 0.3	Min.: 1.08
	Mean: 68	Mean: 244.8
	Max.: 846	Max.: 3045.6
China from this study	Min.: 0.001	Min.: 0.0036
	Mean: 3.6	Mean: 13
	Max.: 4234	Max.: 15244

Note: In this table, the hydroelectric PWF is presented based on the same definition (i.e., the evaporative water consumption for each unit of hydropower generation). Values in m^3^ MWh^−1^ were calculated by multiplying the values in m^3^ GJ^−1^ by 3.6 (the conversion factor).

**Table 3:** Comparison of blue water footprints of different energy carriers.

Energy carrier	Process	Blue water footprint (m^3^/10^12^ J)
Wind energy	Construction, erection and operation of the turbines	0.0^a^
Coal	Surface mining	2–5^a^
	Deep mining	3–20^a^
	Benefication	4^b^
	Slurry pipeline	40–85^b^
	Other plant operation	90^b^
	*Mining, benefication, slurry pipeline and other plant operations*	*136*–*199*
Oil	Onshore oil extraction and production	3–8^a^
	Oil refining	25–65^a^
	Other plant operations	70^a^
	*Onshore oil extraction and production, oil refining and other plant operations*	*98*–*143*
Natural gas	Processing	6^a^
	Pipeline operation	3^a^
	Plant operation	100^a^
	*Gas processing, pipeline operation and plant operations*	109
Coal-powered electricity	Cooling	0.87–15.9^b^
Nuclear powered electricity	Cooling	1.91–12.2^b^
Oil/gas powered electricity	Cooling	1.23–11.2^b^
Solar thermal energy	Cooling and mirror washing	27–1111^a^
Hydropower from China	Evaporation	3600^c^

^a^Gleick^29^

^b^Fthenakis and Kim^30^,

^c^This study (average number).
