# Supplementary material for: China’s rising hydropower demand challenges water sector
Source: Sci Rep. 2015 Jul 9;5:11446. doi: 10.1038/srep11446 (PMC4648423; doi:10.1038/srep11446)
Supplement: Supplementary Information [file srep11446-s1.doc]

**Supplementary Information**

**for**

**China’s rising hydropower demand challenges water sector**

Junguo Liu^1,*^, Dandan Zhao^1^, P.W. Gerbens-Leenes^2^, Dabo Guan^3^

**^1^**School of Nature Conservation, Beijing Forestry University, Qinghua East Road 35, Haidian District, 100083, Beijing, China;

^2^Department of Water Engineering and Management，University of Twente,

P.O. Box 217, 7500 AE, Enschede, The Netherlands

^3^Water Security Research Centre, School of International Development, University of East Anglia, Norwich NR4 7TJ, United Kingdom

*Corresponding author. Correspondence and requests for materials should be addressed to J.L. (E-mail: [junguo.liu@gmail.com](mailto:junguo.liu@gmail.com), [water21water@yahoo.com](mailto:water21water@yahoo.com), Tel.: +86-10-6233-6761, Fax: +86-10-6233-6761)

This Supplementary Information includes **78 pages**, **1 figures** and **8 tables** are included.

**
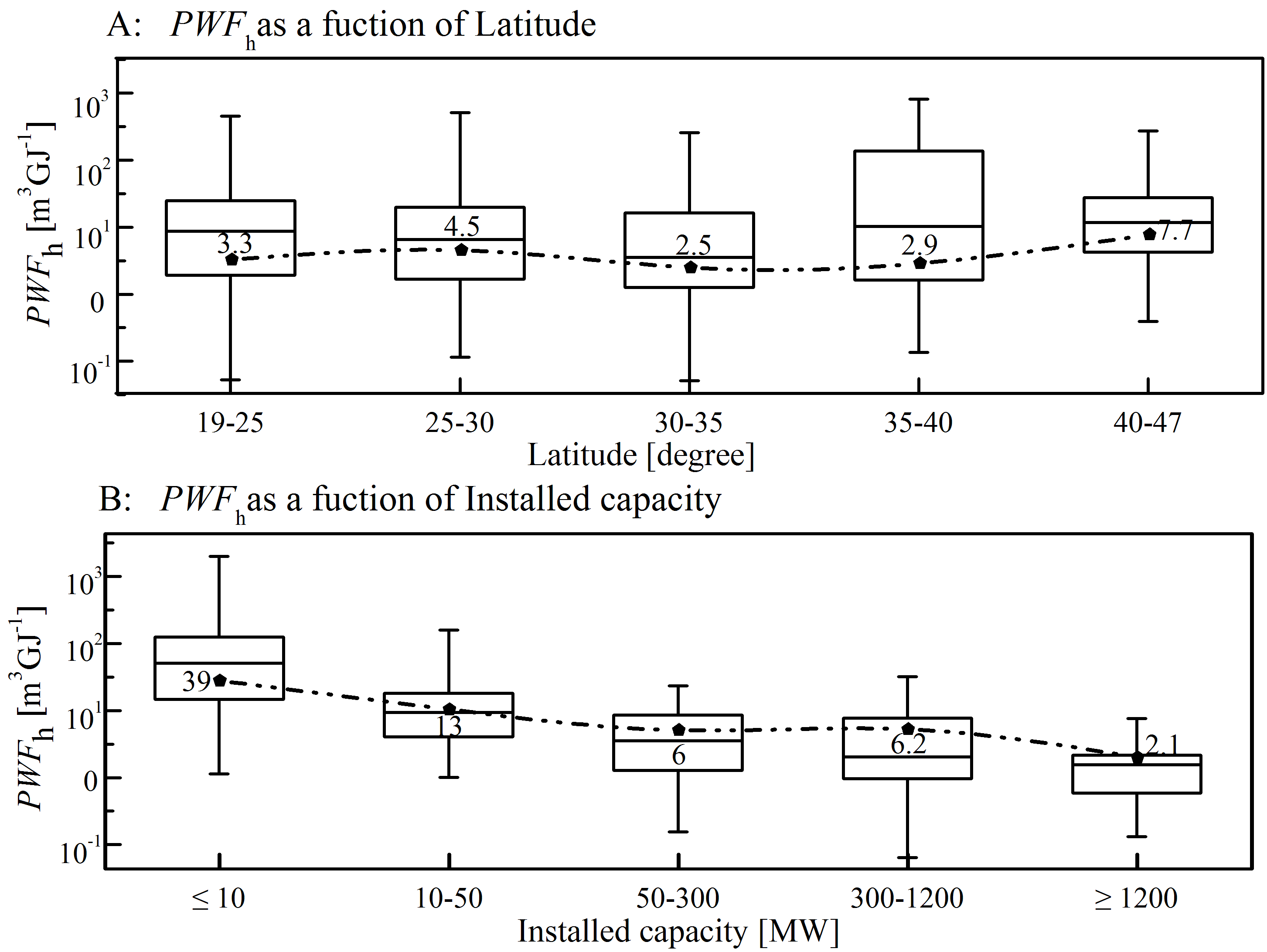
**

**Figure S1. Relationship between the hydroelectric product water footprint (*PWF*_h_) and (A) latitude and (B) installed hydroelectric capacity.**

Table S1. Monthly and annual blue water footprints (WFs) of the 10 Chinese river basins including agricultural, industrial, domestic, and reservoir WFs.

| Basin ID | Basin name | Blue water WF (×10^6^ m^3^/month) | | | | | | | | | | | | |
| --- | --- | --- | --- | --- | --- | --- | --- | --- | --- | --- | --- | --- | --- | --- |
|  |  | Jan. | Feb. | Mar. | Apr. | May | June | July | Aug. | Sept. | Oct. | Nov. | Dec. | Annual |
| 1 | Songhuajiang | 80.4 | 95.1 | 146.6 | 514.1 | 1385.7 | 1982.8 | 1151.4 | 765.3 | 571.0 | 190.6 | 123.0 | 90.0 | 7096.0 |
| 2 | Liaohe | 71.3 | 89.5 | 157.9 | 590.1 | 1546.2 | 2021.5 | 1223.7 | 810.6 | 649.3 | 191.8 | 115.6 | 83.4 | 7550.9 |
| 3 | Haihe | 154.6 | 706.7 | 2451.9 | 4166.2 | 4064.3 | 2109.2 | 2212.3 | 2112.6 | 1264.9 | 532.3 | 172.2 | 146.4 | 20093.5 |
| 4 | Yellow River | 203.6 | 588.4 | 1711.3 | 2926.0 | 2901.8 | 2326.2 | 2367.3 | 1569.7 | 723.7 | 446.7 | 223.8 | 188.1 | 16176.6 |
| 5 | Huaihe | 264.0 | 483.7 | 1190.1 | 2652.7 | 3475.6 | 2947.4 | 2686.7 | 2095.0 | 1916.7 | 628.8 | 333.5 | 271.8 | 18946.1 |
| 6 | Yangtze River | 660.6 | 910.0 | 1369.9 | 2298.2 | 3401.8 | 2876.5 | 4198.9 | 4178.6 | 3667.6 | 1022.9 | 720.3 | 632.0 | 25937.3 |
| 7 | Southeast rivers | 130.8 | 134.9 | 159.7 | 218.5 | 293.7 | 319.8 | 680.8 | 596.6 | 498.0 | 238.1 | 176.8 | 145.3 | 3593.1 |
| 8 | Zhujiang | 391.4 | 425.5 | 518.7 | 844.5 | 1148.3 | 970.2 | 1130.2 | 994.6 | 1135.4 | 552.1 | 454.8 | 397.7 | 8963.4 |
| 9 | Southwest rivers | 133.1 | 166.1 | 232.4 | 443.4 | 466.3 | 248.3 | 156.0 | 144.7 | 162.6 | 98.2 | 89.7 | 98.4 | 2439.4 |
| 10 | Northwest rivers | 82.2 | 168.1 | 540.9 | 1375.2 | 2448.4 | 2438.9 | 2566.5 | 2229.5 | 1553.5 | 391.9 | 145.9 | 66.0 | 14007.1 |
| Total | China | 2172 | 3768 | 8479 | 16029 | 21132 | 18241 | 18374 | 15497 | 12143 | 4294 | 2556 | 2119 | 124803 |

**Table S2. Contribution of reservoir WF to the total blue water WF (*TBWF*) of the 10 Chinese river basins.**

| Basin no. | Basin name | Contribution of WF of reservoirs to the total blue water WF (%) | | | | | | | | | | | | |
| --- | --- | --- | --- | --- | --- | --- | --- | --- | --- | --- | --- | --- | --- | --- |
|  |  | Jan. | Feb. | Mar. | Apr. | May | June | July | Aug. | Sept. | Oct. | Nov. | Dec. | Annual mean |
| 1 | Songhuajiang | 50 | 58 | 68 | 37 | 18 | 13 | 19 | 27 | 32 | 69 | 63 | 52 | 25 |
| 2 | Liaohe | 56 | 63 | 68 | 33 | 17 | 13 | 19 | 27 | 30 | 71 | 66 | 57 | 24 |
| 3 | Haihe | 25 | 7 | 4 | 3 | 4 | 9 | 7 | 7 | 10 | 17 | 35 | 28 | 7 |
| 4 | Yellow River | 33 | 15 | 8 | 7 | 9 | 13 | 12 | 16 | 27 | 32 | 44 | 38 | 13 |
| 5 | Huaihe | 56 | 37 | 22 | 15 | 14 | 19 | 19 | 24 | 22 | 50 | 67 | 60 | 22 |
| 6 | Yangtze River | 46 | 39 | 36 | 30 | 25 | 32 | 26 | 26 | 23 | 59 | 61 | 53 | 31 |
| 7 | Southeast rivers | 70 | 72 | 77 | 74 | 65 | 67 | 41 | 47 | 44 | 73 | 75 | 71 | 57 |
| 8 | Zhujiang | 64 | 61 | 62 | 47 | 42 | 52 | 52 | 56 | 44 | 77 | 74 | 69 | 55 |
| 9 | Southwest rivers | 44 | 47 | 43 | 26 | 25 | 41 | 63 | 67 | 55 | 80 | 73 | 58 | 43 |
| 10 | Northwest rivers | 10 | 8 | 5 | 5 | 4 | 4 | 4 | 5 | 5 | 11 | 13 | 14 | 5 |

Table S3 Monthly water scarcity in the ten Chinese river basins assessed based on WFs (including reservoir WF) and water availability

| Basin no. | Basin name | Water scarcity (%) | | | | | | | | | | | | |
| --- | --- | --- | --- | --- | --- | --- | --- | --- | --- | --- | --- | --- | --- | --- |
|  |  | Jan. | Feb. | Mar. | Apr. | May | June | July | Aug. | Sept. | Oct. | Nov. | Dec. | Annual Mean |
| 1 | Songhuajiang | 9.2 | 498.1 | 171.6 | 17.0 | 49.1 | 88.4 | 37.5 | 20.7 | 18.1 | 9.9 | 12.7 | 15.6 | 31.6 |
| 2 | Liaohe | 30.9 | 500.0 | 148.9 | 121.6 | 234.8 | 336.0 | 163.7 | 60.6 | 74.7 | 40.1 | 42.4 | 52.7 | 126.6 |
| 3 | Haihe | 171.9 | 500.0 | 496.2 | 498.2 | 488.5 | 455.8 | 298.6 | 238.1 | 277.7 | 247.4 | 174.7 | 215.4 | 377.6 |
| 4 | Yellow River | 31.1 | 446.9 | 338.9 | 258.0 | 165.4 | 119.1 | 106.2 | 72.6 | 31.5 | 30.3 | 29.9 | 42.6 | 104.4 |
| 5 | Huaihe | 72.9 | 150.7 | 165.7 | 199.8 | 237.4 | 213.2 | 191.3 | 178.3 | 174.1 | 111.5 | 96.5 | 109.7 | 181.9 |
| 6 | Yangtze River | 7.9 | 20.7 | 13.5 | 12.9 | 14.2 | 10.1 | 16.9 | 17.7 | 17.7 | 7.4 | 8.5 | 12.0 | 13.7 |
| 7 | Southeast rivers | 8.0 | 9.2 | 4.8 | 6.5 | 5.6 | 4.8 | 16.1 | 15.7 | 15.6 | 11.3 | 13.0 | 15.7 | 9.6 |
| 8 | Zhujiang | 16.8 | 78.5 | 39.0 | 24.4 | 14.7 | 7.8 | 10.4 | 8.7 | 15.0 | 12.8 | 17.8 | 25.6 | 13.5 |
| 9 | Southwest rivers | 2.5 | 104.2 | 34.4 | 15.2 | 6.7 | 1.6 | 0.7 | 0.6 | 0.9 | 0.9 | 1.5 | 2.8 | 2.1 |
| 10 | Northwest rivers | 10.3 | 499.2 | 430.7 | 263.0 | 174.8 | 64.2 | 49.5 | 43.1 | 46.4 | 26.0 | 16.6 | 12.5 | 60.1 |

Table S4. Number of months per year when a basin faces a low, moderate, significant, or severe water scarcity, calculated including and excluding the reservoir water footprint (WF).

| Basin no. | Basin name | Number of months per year with a low, moderate, significant, or severe water scarcity | | | | | | | |
| --- | --- | --- | --- | --- | --- | --- | --- | --- | --- |
|  |  | Low | | Moderate | | Significant | | Severe | |
|  |  | Excluding | Including | Excluding | Including | Excluding | Including | Excluding | Including |
| 1 | Songhuajiang | 11 | 10 | 0 | 0 | 0 | 1 | 1 | 1 |
| 2 | Liaohe | 8 | 6 | 1 | 2 | 0 | 1 | 3 | 3 |
| 3 | Haihe | 0 | 0 | 2 | 0 | 1 | 2 | 9 | 10 |
| 4 | Yellow River | 7 | 6 | 1 | 2 | 1 | 1 | 3 | 3 |
| 5 | Huaihe | 4 | 2 | 4 | 2 | 3 | 6 | 1 | 2 |
| 6 | Yangtze River | 12 | 12 | 0 | 0 | 0 | 0 | 0 | 0 |
| 7 | Southeast rivers | 12 | 12 | 0 | 0 | 0 | 0 | 0 | 0 |
| 8 | Zhujiang | 12 | 12 | 0 | 0 | 0 | 0 | 0 | 0 |
| 9 | Southwest rivers | 12 | 11 | 0 | 1 | 0 | 0 | 0 | 0 |
| 10 | Northwest rivers | 8 | 8 | 0 | 0 | 1 | 1 | 3 | 3 |

**Table S5. Product water footprint (PWF) of the 209 selected hydroelectric power plants**.

| Basin no. | Basin name | Reservoir name | Evaporation | Surface area | *PWF* | Hydroelectric power generation | η | *PWF*_h_ |
| --- | --- | --- | --- | --- | --- | --- | --- | --- |
|  |  |  | [mm yr^-1^] | [km^2^] | [Gm^3^ yr^-1^] | [GJ yr^-1^] |  | [m^3^GJ^-1^] |
| 1 | Songhuajiang | Baishan | 968 | 84.8 | 0.082 | 12528000 | 0.48 | 3.1 |
| 1 | Songhuajiang | Fengman | 1300 | 193.3 | 0.251 | 8226000 | 0.48 | 14.7 |
| 1 | Songhuajiang | Huanren | 1014 | 89.5 | 0.091 | 3801600 | 0.48 | 11.5 |
| 1 | Songhuajiang | Laohushao | 1001 | 30.5 | 0.031 | 4320000 | 0.48 | 3.4 |
| 1 | Songhuajiang | Shuifeng | 1004 | 284.5 | 0.286 | 12528000 | 0.48 | 10.9 |
| 1 | Songhuajiang | Taipingshao | 1017 | 17.1 | 0.017 | 2059200 | 0.48 | 4.1 |
| 1 | Songhuajiang | Taipingwan | 1000 | 31.7 | 0.032 | 2592000 | 0.48 | 5.9 |
| 1 | Songhuajiang | Yunfeng | 1006 | 45.9 | 0.046 | 6300000 | 0.48 | 3.5 |
| 2 | Liaohe | Chaihe | 1073 | 20.8 | 0.022 | 57600 | 0.15 | 58.1 |
| 2 | Liaohe | Erlongshan | 797 | 98.2 | 0.078 | 626400 | 0.09 | 10.7 |
| 2 | Liaohe | Hongshan | 1054 | 59.4 | 0.063 | 36000 | 0.15 | 260.9 |
| 2 | Liaohe | Nanchengzi | 1081 | 13.3 | 0.014 | 9720 | 0.15 | 221.8 |
| 2 | Liaohe | Shandaohe | 1062 | 3.0 | 0.003 | 3600 | 0.15 | 132.8 |
| 2 | Liaohe | Shangying | 1056 | 2.3 | 0.002 | 336312 | 0.15 | 1.1 |
| 2 | Liaohe | Shenwo | 1059 | 31.9 | 0.034 | 288000 | 0.15 | 17.6 |
| 2 | Liaohe | Tumenzi | 995 | 7.2 | 0.007 | 23760 | 0.15 | 45.2 |
| 3 | Haihe | Gangnan | 1171 | 39.2 | 0.046 | 306000 | 0.09 | 12.9 |
| 3 | Haihe | Guanting | 1111 | 90.1 | 0.100 | 324000 | 0.09 | 26.5 |
| 3 | Haihe | Hengshanling | 1194 | 7.0 | 0.008 | 5976 | 0.15 | 209.8 |
| 3 | Haihe | Koutou | 1211 | 4.3 | 0.005 | 1224 | 0.15 | 638.3 |
| 3 | Haihe | Miyun | 1316 | 121.7 | 0.160 | 414000 | 0.06 | 21.6 |
| 3 | Haihe | Panjiakou | 1233 | 18.0 | 0.022 | 226800 | 0.15 | 14.7 |
| 3 | Haihe | Xidayang | 983 | 32.4 | 0.032 | 144000 | 0.09 | 19.0 |
| 3 | Haihe | Yanghe | 929 | 13.9 | 0.013 | 14760 | 0.15 | 131.2 |
| 3 | Haihe | Yuecheng | 1226 | 15.2 | 0.019 | 162000 | 0.09 | 9.9 |
| 4 | Yellow River | Baijiazui | 1000 | 11.6 | 0.012 | 14400 | 0.15 | 120.8 |
| 4 | Yellow River | Bapanxia | 969 | 4.9 | 0.005 | 3754800 | 0.74 | 0.9 |
| 4 | Yellow River | Daxia | 982 | 2.8 | 0.003 | 5274000 | 0.50 | 0.3 |
| 4 | Yellow River | Fenhe | 1000 | 21.1 | 0.021 | 40680 | 0.51 | 263.5 |
| 4 | Yellow River | Gongboxia | 975 | 17.0 | 0.017 | 21258000 | 0.48 | 0.4 |
| 4 | Yellow River | Jinpen | 968 | 2.2 | 0.002 | 263088 | 0.15 | 1.2 |
| 4 | Yellow River | Laxiwa | 1100 | 11.8 | 0.013 | 34801200 | 0.48 | 0.2 |
| 4 | Yellow River | Liujiaxia | 1000 | 115.5 | 0.116 | 20088000 | 0.57 | 3.3 |
| 4 | Yellow River | Longyangxia | 1100 | 284.7 | 0.313 | 24894000 | 0.74 | 9.3 |
| 4 | Yellow River | Luhun | 990 | 57.3 | 0.057 | 34560 | 0.15 | 246.2 |
| 4 | Yellow River | Muyu | 1120 | 2.8 | 0.003 | 11207 | 0.15 | 42.0 |
| 4 | Yellow River | Sanmenxia | 1200 | 254.8 | 0.306 | 4716000 | 0.15 | 9.7 |
| 4 | Yellow River | Taihe | 1275 | 6.1 | 0.008 | 2412000 | 0.48 | 1.5 |
| 4 | Yellow River | Wangyao | 900 | 9.7 | 0.009 | 5400 | 0.48 | 776.0 |
| 4 | Yellow River | Wanjiazhai | 1120 | 22.1 | 0.025 | 9900000 | 0.88 | 2.2 |
| 4 | Yellow River | Xiaolangdi | 1560 | 61.6 | 0.096 | 18705600 | 0.32 | 1.6 |
| 4 | Yellow River | Xueye | 1283 | 6.1 | 0.008 | 8928 | 0.15 | 131.4 |
| 4 | Yellow River | Yanguoxia | 1100 | 17.3 | 0.019 | 8586000 | 0.74 | 1.6 |
| 4 | Yellow River | Zhaikou | 1000 | 6.0 | 0.006 | 51120 | 0.15 | 17.6 |
| 4 | Yellow River | Zhiganglaka | 977 | 0.7 | 0.001 | 2534400 | 0.48 | 0.1 |
| 5 | Huaihe | Baiguishan | 1176 | 33.7 | 0.040 | 29340 | 0.15 | 202.7 |
| 5 | Huaihe | Nanwan | 1137 | 39.3 | 0.045 | 54000 | 0.15 | 124.1 |
| 6 | Yangtze River | Ankang | 748 | 57.3 | 0.043 | 10285200 | 0.48 | 2.0 |
| 6 | Yangtze River | Bailianhe | 1135 | 32.8 | 0.037 | 302400 | 0.48 | 59.1 |
| 6 | Yangtze River | Baiyun | 948 | 7.6 | 0.007 | 420480 | 0.48 | 8.2 |
| 6 | Yangtze River | Baiyunshan 1 | 1171 | 1.3 | 0.002 | 242280 | 0.48 | 3.0 |
| 6 | Yangtze River | Baiyutan | 1086 | 21.9 | 0.024 | 3202416 | 0.48 | 3.6 |
| 6 | Yangtze River | Baozhusi | 954 | 66.3 | 0.063 | 4320000 | 0.48 | 7.0 |
| 6 | Yangtze River | Bikou | 966 | 7.2 | 0.007 | 5266800 | 0.48 | 0.6 |
| 6 | Yangtze River | Cheba 1 | 979 | 2.1 | 0.002 | 307080 | 0.15 | 1.0 |
| 6 | Yangtze River | Chongjianghe | 1016 | 0.0 | 0.000 | 694800 | 0.48 | 0.0 |
| 6 | Yangtze River | Daduan | 1102 | 7.2 | 0.008 | 155880 | 0.48 | 24.4 |
| 6 | Yangtze River | Dahonghe | 965 | 13.0 | 0.013 | 39852 | 0.48 | 151.1 |
| 6 | Yangtze River | Dahongshan | 1114 | 11.0 | 0.012 | 1608516 | 0.15 | 1.1 |
| 6 | Yangtze River | Dahua | 954 | 7.7 | 0.007 | 2199600 | 0.48 | 1.6 |
| 6 | Yangtze River | Danjiangkou | 1000 | 286.3 | 0.286 | 13788000 | 0.75 | 15.6 |
| 6 | Yangtze River | Dongjiang | 1086 | 130.6 | 0.142 | 4752000 | 0.48 | 14.3 |
| 6 | Yangtze River | Dongjin | 1108 | 25.3 | 0.028 | 419040 | 0.48 | 32.1 |
| 6 | Yangtze River | Doulingzi | 1021 | 17.8 | 0.018 | 874800 | 0.48 | 10.0 |
| 6 | Yangtze River | Ertan | 1270 | 65.5 | 0.083 | 56829600 | 0.91 | 1.4 |
| 6 | Yangtze River | Fangtuan | 1180 | 2.2 | 0.003 | 307080 | 0.48 | 4.1 |
| 6 | Yangtze River | Fengdu | 1068 | 2.1 | 0.002 | 145584 | 0.48 | 7.4 |
| 6 | Yangtze River | Fengtan | 1003 | 15.6 | 0.016 | 7354800 | 0.48 | 1.0 |
| 6 | Yangtze River | Fushui | 1136 | 56.8 | 0.065 | 432000 | 0.15 | 22.4 |
| 6 | Yangtze River | Gaofang | 1159 | 4.7 | 0.005 | 10800 | 0.15 | 75.7 |
| 6 | Yangtze River | Gaoyan | 1049 | 2.1 | 0.002 | 43200 | 0.27 | 13.5 |
| 6 | Yangtze River | Geheyan | 994 | 41.0 | 0.041 | 10944000 | 0.48 | 1.8 |
| 6 | Yangtze River | Gezhouba | 753 | 53.2 | 0.040 | 58467600 | 0.92 | 0.6 |
| 6 | Yangtze River | Gongzui | 919 | 12.0 | 0.011 | 23029200 | 0.48 | 0.2 |
| 6 | Yangtze River | Goupitan | 930 | 74.9 | 0.070 | 20102400 | 0.48 | 1.7 |
| 6 | Yangtze River | Guanli | 1174 | 0.8 | 0.001 | 482544 | 0.15 | 0.3 |
| 6 | Yangtze River | Gudongkou | 1031 | 3.0 | 0.003 | 439200 | 0.48 | 3.4 |
| 6 | Yangtze River | Hongfeng | 993 | 43.4 | 0.043 | 2106000 | 0.48 | 9.8 |
| 6 | Yangtze River | Hongjiang | 1017 | 11.6 | 0.012 | 2854800 | 0.48 | 2.0 |
| 6 | Yangtze River | Hongmen | 1170 | 34.9 | 0.041 | 432000 | 0.15 | 14.2 |
| 6 | Yangtze River | Hongyi | 986 | 0.0 | 0.000 | 1328400 | 0.48 | 0.0 |
| 6 | Yangtze River | Huamuqiao | 1093 | 2.0 | 0.002 | 513216 | 0.48 | 2.0 |
| 6 | Yangtze River | Huangcai | 1019 | 8.9 | 0.009 | 46368 | 0.48 | 93.9 |
| 6 | Yangtze River | Huanglongtan | 1049 | 12.7 | 0.013 | 2732400 | 0.48 | 2.3 |
| 6 | Yangtze River | Jiangya | 989 | 26.5 | 0.026 | 2721600 | 0.15 | 1.4 |
| 6 | Yangtze River | Jiaoyuan | 1166 | 2.0 | 0.002 | 292464 | 0.48 | 3.8 |
| 6 | Yangtze River | Jiufujiang | 1069 | 13.2 | 0.014 | 111234 | 0.15 | 19.0 |
| 6 | Yangtze River | Juntan | 1170 | 3.2 | 0.004 | 93600 | 0.48 | 19.2 |
| 6 | Yangtze River | Laoyingyan | 917 | 3.0 | 0.003 | 8424000 | 0.15 | 0.0 |
| 6 | Yangtze River | Lianbu | 1066 | 0.1 | 0.000 | 2138400 | 0.48 | 0.0 |
| 6 | Yangtze River | Liangcha | 965 | 3.4 | 0.003 | 33336 | 0.15 | 14.8 |
| 6 | Yangtze River | Linjintan | 1019 | 23.6 | 0.024 | 4183200 | 0.48 | 2.8 |
| 6 | Yangtze River | Liujiaping | 994 | 2.8 | 0.003 | 194400 | 0.48 | 6.9 |
| 6 | Yangtze River | Lugu | 1057 | 1.2 | 0.001 | 1080000 | 0.48 | 0.6 |
| 6 | Yangtze River | Lushui | 936 | 33.0 | 0.031 | 45864000 | 0.48 | 0.3 |
| 6 | Yangtze River | Majitang | 1026 | 7.3 | 0.007 | 694800 | 0.48 | 5.2 |
| 6 | Yangtze River | Maojiacun | 1132 | 14.4 | 0.016 | 262800 | 0.48 | 29.8 |
| 6 | Yangtze River | Maojianshan | 1094 | 2.0 | 0.002 | 144000 | 0.48 | 7.3 |
| 6 | Yangtze River | Mingyangguan | 1167 | 2.6 | 0.003 | 45000 | 0.48 | 32.4 |
| 6 | Yangtze River | Nanhe | 1052 | 4.5 | 0.005 | 385200 | 0.48 | 5.9 |
| 6 | Yangtze River | Pengshui | 942 | 45.7 | 0.043 | 17521200 | 0.48 | 1.2 |
| 6 | Yangtze River | Pubugou | 952 | 220.6 | 0.210 | 36644400 | 0.48 | 2.8 |
| 6 | Yangtze River | Puding | 1018 | 8.2 | 0.008 | 1224000 | 0.48 | 3.3 |
| 6 | Yangtze River | Qingshan | 1196 | 9.4 | 0.011 | 284544 | 0.15 | 5.9 |
| 6 | Yangtze River | Sanbanxi | 989 | 165.5 | 0.164 | 4683600 | 0.48 | 16.8 |
| 6 | Yangtze River | Sanjiangkou | 1037 | 24.1 | 0.025 | 450000 | 0.48 | 26.6 |
| 6 | Yangtze River | Shangyoujiang | 1503 | 19.4 | 0.029 | 831600 | 0.48 | 16.8 |
| 6 | Yangtze River | Shawan | 938 | 1.6 | 0.002 | 6519600 | 0.48 | 0.1 |
| 6 | Yangtze River | Shibikeng | 1218 | 5.7 | 0.007 | 31442 | 0.48 | 106.0 |
| 6 | Yangtze River | Shiquan | 943 | 21.9 | 0.021 | 2815200 | 0.48 | 3.5 |
| 6 | Yangtze River | Shiziping | 989 | 4.6 | 0.005 | 489600 | 0.48 | 4.5 |
| 6 | Yangtze River | Shizitan | 966 | 29.2 | 0.028 | 741600 | 0.48 | 18.3 |
| 6 | Yangtze River | Shuangpai | 1066 | 40.3 | 0.043 | 2106000 | 0.48 | 9.8 |
| 6 | Yangtze River | Shuhe | 1002 | 5.7 | 0.006 | 1191600 | 0.48 | 2.3 |
| 6 | Yangtze River | Shuifumiao | 1042 | 21.0 | 0.022 | 392400 | 0.48 | 26.8 |
| 6 | Yangtze River | Shuiniujia | 961 | 3.1 | 0.003 | 756000 | 0.48 | 1.9 |
| 6 | Yangtze River | Silin | 944 | 38.4 | 0.036 | 9021600 | 0.48 | 1.9 |
| 6 | Yangtze River | Suofengying | 950 | 4.2 | 0.004 | 4687200 | 0.48 | 0.4 |
| 6 | Yangtze River | Three Gorges Dam | 978 | 852.9 | 0.834 | 303732000 | 0.72 | 2.0 |
| 6 | Yangtze River | Tieshan | 1087 | 51.2 | 0.056 | 57600 | 0.48 | 463.6 |
| 6 | Yangtze River | Tongjiezi | 932 | 7.7 | 0.007 | 11556000 | 0.74 | 0.5 |
| 6 | Yangtze River | Tongtou | 946 | 0.7 | 0.001 | 1684800 | 0.48 | 0.2 |
| 6 | Yangtze River | Tuanjie | 1157 | 14.8 | 0.017 | 10584 | 0.15 | 242.6 |
| 6 | Yangtze River | Wanan | 1172 | 67.6 | 0.079 | 5745600 | 0.48 | 6.6 |
| 6 | Yangtze River | Wujiangdu | 959 | 32.3 | 0.031 | 9237600 | 0.48 | 1.6 |
| 6 | Yangtze River | Wumu | 975 | 9.5 | 0.009 | 1389168 | 0.15 | 1.0 |
| 6 | Yangtze River | Wuqiangxi | 1007 | 113.8 | 0.115 | 19929600 | 0.07 | 0.4 |
| 6 | Yangtze River | Xinqiao | 970 | 0.9 | 0.001 | 11268 | 0.48 | 37.2 |
| 6 | Yangtze River | Xuecheng | 984 | 90.5 | 0.089 | 2106000 | 0.48 | 20.3 |
| 6 | Yangtze River | Yahekou | 1136 | 42.1 | 0.048 | 72000 | 0.15 | 99.6 |
| 6 | Yangtze River | Yilihe | 1136 | 17.7 | 0.020 | 3157200 | 0.48 | 3.1 |
| 6 | Yangtze River | Yinzidu | 965 | 19.6 | 0.019 | 2592000 | 0.48 | 3.5 |
| 6 | Yangtze River | Yudong | 1043 | 7.1 | 0.007 | 3240000 | 0.15 | 0.3 |
| 6 | Yangtze River | Yutang | 949 | 3.7 | 0.004 | 820800 | 0.48 | 2.1 |
| 6 | Yangtze River | Zhanggang | 1203 | 13.2 | 0.016 | 1800 | 0.48 | 4234.3 |
| 6 | Yangtze River | Zhelin | 1143 | 201.6 | 0.230 | 2268000 | 0.15 | 15.2 |
| 6 | Yangtze River | Zhexi | 993 | 48.8 | 0.048 | 7826400 | 0.48 | 3.0 |
| 6 | Yangtze River | Zhushuqiao | 1074 | 8.8 | 0.009 | 261360 | 0.48 | 17.4 |
| 6 | Yangtze River | Zhuxikou | 1006 | 1.1 | 0.001 | 896400 | 0.48 | 0.6 |
| 6 | Yangtze River | Zilanba | 946 | 1.5 | 0.001 | 979200 | 0.48 | 0.7 |
| 6 | Yangtze River | Ziyunshan | 1164 | 12.1 | 0.014 | 4320 | 0.15 | 488.9 |
| 7 | Southeast rivers | Ansha | 900 | 34.3 | 0.031 | 2304000 | 0.48 | 6.4 |
| 7 | Southeast rivers | Changtan | 1129 | 23.2 | 0.026 | 612000 | 0.15 | 6.4 |
| 7 | Southeast rivers | Chitan | 817 | 57.9 | 0.047 | 1972800 | 0.48 | 11.5 |
| 7 | Southeast rivers | Dongzhang | 1187 | 10.1 | 0.012 | 4680 | 0.48 | 1229.1 |
| 7 | Southeast rivers | Dongzhen | 1547 | 12.5 | 0.019 | 106178 | 0.53 | 96.2 |
| 7 | Southeast rivers | Fuchunjiang | 1111 | 45.2 | 0.050 | 4215600 | 0.15 | 1.8 |
| 7 | Southeast rivers | Hengjin | 1320 | 9.8 | 0.013 | 72720 | 0.15 | 26.7 |
| 7 | Southeast rivers | Hunanzhen | 1136 | 44.0 | 0.050 | 1944000 | 0.48 | 12.3 |
| 7 | Southeast rivers | Lishimen | 1076 | 9.2 | 0.010 | 89280 | 0.15 | 16.6 |
| 7 | Southeast rivers | Niutoushan | 993 | 13.0 | 0.013 | 1008000 | 0.48 | 6.1 |
| 7 | Southeast rivers | Shanzi | 1154 | 5.3 | 0.006 | 622800 | 0.48 | 4.7 |
| 7 | Southeast rivers | Shaxikou | 1176 | 14.3 | 0.017 | 4255200 | 0.48 | 1.9 |
| 7 | Southeast rivers | Shuikou | 1000 | 56.2 | 0.056 | 25106400 | 0.48 | 1.1 |
| 7 | Southeast rivers | Taihu | 988 | 3.3 | 0.003 | 117000 | 0.15 | 4.2 |
| 7 | Southeast rivers | Wananxi | 1000 | 10.6 | 0.011 | 488520 | 0.15 | 3.3 |
| 7 | Southeast rivers | Xinan Jiang | 1215 | 424.3 | 0.516 | 8578800 | 0.48 | 28.9 |
| 7 | Southeast rivers | Zhaokou | 1177 | 2.0 | 0.002 | 896400 | 0.48 | 1.3 |
| 8 | Zhujiang | Bailongtan | 1176 | 12.4 | 0.015 | 2995200 | 0.48 | 2.3 |
| 8 | Zhujiang | Chaishitan | 1178 | 4.0 | 0.005 | 658800 | 0.15 | 1.1 |
| 8 | Zhujiang | Changhu | 1235 | 8.2 | 0.010 | 1224000 | 0.48 | 4.0 |
| 8 | Zhujiang | Chengbihe | 1218 | 25.0 | 0.030 | 410400 | 0.48 | 35.6 |
| 8 | Zhujiang | Dakai | 1184 | 14.9 | 0.018 | 918612 | 0.15 | 2.9 |
| 8 | Zhujiang | Dalongdong | 1247 | 14.1 | 0.018 | 13245 | 0.15 | 199.2 |
| 8 | Zhujiang | Dawangtan | 1224 | 16.1 | 0.020 | 16668 | 0.06 | 69.2 |
| 8 | Zhujiang | Dongjing | 1078 | 17.2 | 0.019 | 9306000 | 0.48 | 1.0 |
| 8 | Zhujiang | Dongluo | 1131 | 2.2 | 0.002 | 54000 | 0.48 | 22.1 |
| 8 | Zhujiang | Etan | 1167 | 36.0 | 0.042 | 9550800 | 0.48 | 2.1 |
| 8 | Zhujiang | Fucao | 1231 | 2.4 | 0.003 | 158400 | 0.48 | 9.0 |
| 8 | Zhujiang | Gaozhou | 1259 | 21.4 | 0.027 | 364140 | 0.15 | 11.1 |
| 8 | Zhujiang | Guangxichangzhou | 1225 | 313.3 | 0.384 | 9684000 | 0.48 | 19.0 |
| 8 | Zhujiang | Guangzhao | 1076 | 125.3 | 0.135 | 6886800 | 0.48 | 9.4 |
| 8 | Zhujiang | Guangzhou pump storage | 1257 | 2.4 | 0.003 | 13521600 | 0.48 | 0.1 |
| 8 | Zhujiang | Guishi | 1104 | 34.1 | 0.038 | 237600 | 0.15 | 23.8 |
| 8 | Zhujiang | Hemianshi | 1141 | 9.9 | 0.011 | 1296000 | 0.15 | 1.3 |
| 8 | Zhujiang | Hongchaojiang | 1238 | 25.2 | 0.031 | 10836 | 0.15 | 431.7 |
| 8 | Zhujiang | Hongjiadu | 1004 | 201.7 | 0.202 | 2134800 | 0.48 | 45.5 |
| 8 | Zhujiang | Huanglongdai | 1246 | 7.5 | 0.009 | 91080 | 0.48 | 49.2 |
| 8 | Zhujiang | Huashan | 1191 | 3.5 | 0.004 | 4860 | 0.48 | 411.7 |
| 8 | Zhujiang | Jinjiang | 1232 | 23.9 | 0.029 | 173315 | 0.48 | 81.5 |
| 8 | Zhujiang | Jinjitan | 1229 | 13.0 | 0.016 | 914400 | 0.48 | 8.4 |
| 8 | Zhujiang | Liuchen | 1218 | 10.0 | 0.012 | 33120 | 0.15 | 55.1 |
| 8 | Zhujiang | Liuxihe | 1247 | 9.5 | 0.012 | 554400 | 0.48 | 10.3 |
| 8 | Zhujiang | Longtan | 1139 | 696.4 | 0.793 | 41306400 | 0.48 | 9.2 |
| 8 | Zhujiang | Lubuge | 1040 | 4.1 | 0.004 | 8488800 | 0.08 | 0.1 |
| 8 | Zhujiang | Mashi | 1053 | 20.8 | 0.022 | 1638000 | 0.48 | 6.4 |
| 8 | Zhujiang | Niululing | 1412 | 25.3 | 0.036 | 1011600 | 0.48 | 17.0 |
| 8 | Zhujiang | Pingban | 1130 | 10.0 | 0.011 | 3686400 | 0.48 | 1.5 |
| 8 | Zhujiang | Quanshui | 1129 | 2.2 | 0.002 | 410400 | 0.48 | 2.9 |
| 8 | Zhujiang | Shanxiu | 1236 | 22.5 | 0.028 | 1144800 | 0.48 | 11.7 |
| 8 | Zhujiang | Shuangdao | 1188 | 4.3 | 0.005 | 196524 | 0.48 | 12.5 |
| 8 | Zhujiang | Tangxi | 1457 | 10.8 | 0.016 | 82800 | 0.48 | 91.2 |
| 8 | Zhujiang | Tianshengqiao 1 | 1085 | 98.3 | 0.107 | 18720000 | 0.29 | 1.7 |
| 8 | Zhujiang | Tuanpo | 1054 | 0.0 | 0.000 | 828000 | 0.48 | 0.0 |
| 8 | Zhujiang | Xiaojiang | 1218 | 29.5 | 0.036 | 2577600 | 0.48 | 6.7 |
| 8 | Zhujiang | Xijin | 1208 | 66.5 | 0.080 | 3420000 | 0.48 | 11.3 |
| 8 | Zhujiang | Xinfengjiang | 852 | 264.3 | 0.225 | 2566800 | 0.26 | 22.8 |
| 8 | Zhujiang | Yantan | 1646 | 85.1 | 0.140 | 21736800 | 0.48 | 3.1 |
| 9 | Southwest rivers | Dachaoshan | 1184 | 20.2 | 0.024 | 22410000 | 0.48 | 0.5 |
| 9 | Southwest rivers | Dayingjiang | 1118 | 0.0 | 0.000 | 1710000 | 0.48 | 0.0 |
| 9 | Southwest rivers | Gelantan | 1214 | 9.1 | 0.011 | 5796000 | 0.48 | 0.9 |
| 9 | Southwest rivers | Jinghong | 1162 | 43.5 | 0.051 | 22233600 | 0.48 | 1.1 |
| 9 | Southwest rivers | Malutang | 1220 | 9.2 | 0.011 | 1616400 | 0.48 | 3.3 |
| 9 | Southwest rivers | Qiezishan Reservoir | 1157 | 3.1 | 0.004 | 238464 | 0.48 | 7.2 |
| 9 | Southwest rivers | Xiaowan | 1196 | 166.4 | 0.199 | 51764400 | 0.48 | 1.8 |
| 9 | Southwest rivers | Yamzho Yumco | 1048 | 566.9 | 0.594 | 330480 | 0.48 | 862.7 |
| 10 | Northwest rivers | Chahanwusu | 1327 | 2.6 | 0.003 | 4442400 | 0.48 | 0.4 |
| 10 | Northwest rivers | Jilintai 1 | 1002 | 100.1 | 0.100 | 5569200 | 0.48 | 8.6 |
| 10 | Northwest rivers | Kalangguer | 1023 | 2.0 | 0.002 | 36000 | 0.15 | 8.5 |

**Table S6 Basic data, evaporation (*E*), and water footprint (WF) data for the 875 representative reservoirs in China. Basin numbers are shown in Figures 1 and S1.**

| Basin number | Basin name | Reservoir id | Reservoir name | Longitude | Latitude | Volume | Area | Use for hydroelectricity | Installed hydroelectric capacity | Hydroelectric generation | E | Reservoir WF |
| --- | --- | --- | --- | --- | --- | --- | --- | --- | --- | --- | --- | --- |
|  |  |  |  | (°E) | (°N) | (×10^6^ m^3^) | (km^2^) |  | (MW) | (GWh) | (mm yr^-1^) | (×10^6^ m^3^) |
| 1 | Songhuajiang | 96 | Baishan | 127.22 | 42.73 | 5320 | 84.8 | Main | 1700^b^ | 3480^b^ | 968 | 82 |
| 1 | Songhuajiang | 808 | Daxin | 129.40 | 42.64 | 19 | 0.9 | - | - | - | 908 | 0.82 |
| 1 | Songhuajiang | 97 | Fengman | 126.69 | 43.72 | 9170 | 193.3 | Main | 862.5^b^ | 2285^b^ | 1300^a^ | 251 |
| 1 | Songhuajiang | 778 | Guanmenshan | 124.14 | 41.13 | 81 | 2.2 | - | - | - | 1031 | 2 |
| 1 | Songhuajiang | 816 | Hongshi | 127.13 | 42.95 | 149 | 7.7 | - | - | - | 969 | 7 |
| 1 | Songhuajiang | 94 | Huanren | 125.40 | 41.29 | 3450 | 89.5 | Main | 294.5^b^ | 1056^b^ | 1014 | 91 |
| 1 | Songhuajiang | 821 | Huashouchuan | 129.67 | 44.10 | 119 | 5.1 | - | - | - | 896 | 5 |
| 1 | Songhuajiang | 776 | Huilongshan | 125.31 | 41.09 | 123 | 11.7 | - | - | - | 1016 | 12 |
| 1 | Songhuajiang | 92 | Laohushao | 125.97 | 40.90 | 630 | 30.5 | Main | 65 | 1200 | 1001 | 31 |
| 1 | Songhuajiang | 820 | Shitoukoumen | 125.75 | 43.96 | 1502 | 58.1 | - | - | - | 1026 | 60 |
| 1 | Songhuajiang | 91 | Shuifeng | 124.97 | 40.46 | 14700 | 284.5 | Main | 630 | 3480 | 1004 | 286 |
| 1 | Songhuajiang | 830 | Songjianghe | 127.27 | 42.36 | 535 | 19.8 | - | 510^b^ | 949^b^ | 974 | 19 |
| 1 | Songhuajiang | 93 | Taipingshao | 125.27 | 40.96 | 289 | 17.1 | Main | 161^b^ | 572^b^ | 1017 | 17 |
| 1 | Songhuajiang | 90 | Taipingwan | 124.73 | 40.35 | 270 | 31.7 | Main | 190 | 720 | 1000 | 32 |
| 1 | Songhuajiang | 812 | Yadong | 129.09 | 42.73 | 41 | 2.1 | - | - | - | 906 | 2 |
| 1 | Songhuajiang | 95 | Yunfeng | 126.51 | 41.38 | 3911 | 45.9 | Main | 400^b^ | 1750^b^ | 1006 | 46 |
| 2 | Liaohe | 795 | Babaohai | 122.23 | 42.15 | 20 | 2.4 | - | - | - | 1099 | 3 |
| 2 | Liaohe | 785 | Baishan | 119.45 | 41.74 | 30 | 2.3 | - | 300^b^ | 87^b^ | 1178 | 3 |
| 2 | Liaohe | 782 | Baishi | 121.00 | 41.68 | 1645 | 10.1 | - | - | - | 1156 | 12 |
| 2 | Liaohe | 755 | Biliuhe | 122.50 | 39.82 | 930 | 32.2 | - | - | - | 1033 | 33 |
| 2 | Liaohe | 205 | Chaihe | 123.99 | 42.27 | 650 | 20.8 | Sec | 7 | 16 | 1073 | 22 |
| 2 | Liaohe | 764 | Dafengkou | 119.95 | 40.24 | 104 | 4.0 | - | - | - | 1155 | 5 |
| 2 | Liaohe | 789 | Dahuofang | 124.10 | 41.89 | 2187 | 51.1 | - | - | 46 | 843^a^ | 43 |
| 2 | Liaohe | 809 | Daqinggou | 122.21 | 42.68 | 10 | 0.6 | - | - | - | 1101 | 0.66 |
| 2 | Liaohe | 208 | Erlongshan | 124.79 | 43.19 | 1800 | 98.2 | Sec | 51 | 174 | 796.6^a^ | 78 |
| 2 | Liaohe | 773 | Gedalou | 122.14 | 40.94 | 40 | 15.1 | - | - | - | 1083 | 16 |
| 2 | Liaohe | 770 | Gongshanzui | 119.76 | 40.74 | 129 | 3.0 | - | - | - | 1177 | 4 |
| 2 | Liaohe | 779 | Guanyinge | 124.15 | 41.32 | 2170 | 34.8 | - | - | - | 1042 | 36 |
| 2 | Liaohe | 814 | Hongdingshan | 124.04 | 42.83 | 10 | 2.2 | - | - | - | 1089 | 2 |
| 2 | Liaohe | 207 | Hongshan 1 | 119.70 | 42.75 | 2560 | 59.4 | Sec | 7 | 10 | 1054^a^ | 63 |
| 2 | Liaohe | 815 | Hongshan 2 | 124.12 | 42.89 | 10 | 2.5 | - | - | - | 1087 | 3 |
| 2 | Liaohe | 783 | Hongsheng | 125.14 | 41.70 | 40 | 3.3 | - | - | - | 1035 | 3 |
| 2 | Liaohe | 792 | Houlou | 125.13 | 41.95 | 15 | 2.4 | - | - | - | 1039 | 2 |
| 2 | Liaohe | 799 | Huanzidong | 122.98 | 42.37 | 40 | 18.3 | - | - | - | 1095 | 20 |
| 2 | Liaohe | 797 | Jianguo | 121.91 | 42.28 | 10 | 1.3 | - | - | - | 1108 | 1.44 |
| 2 | Liaohe | 804 | Jianshanzi | 123.00 | 42.52 | 10 | 4.6 | - | - | - | 1090 | 5 |
| 2 | Liaohe | 806 | Julonghu | 122.76 | 42.59 | 10 | 4.4 | - | - | - | 1085 | 5 |
| 2 | Liaohe | 801 | Lamazhang | 122.96 | 42.47 | 10 | 3.3 | - | - | - | 1091 | 4 |
| 2 | Liaohe | 786 | Liushan | 123.79 | 41.78 | 9 | 0.6 | - | - | - | 1085 | 0.65 |
| 2 | Liaohe | 787 | Longtan | 120.44 | 41.81 | 44 | 1.4 | - | - | - | 1173 | 2 |
| 2 | Liaohe | 788 | Longwan | 122.11 | 41.86 | 70 | 4.3 | - | - | - | 1115 | 5 |
| 2 | Liaohe | 760 | Luoquanbei | 123.49 | 40.04 | 51 | 1.6 | - | - | - | 1007 | 2 |
| 2 | Liaohe | 817 | Morin Sum | 121.82 | 43.54 | 200 | 21.2 | - | - | - | 1122 | 24 |
| 2 | Liaohe | 206 | Nanchengzi | 124.30 | 42.74 | 206 | 13.3 | Sec | - | 3 | 1081 | 14 |
| 2 | Liaohe | 810 | Naodehai | 122.17 | 42.68 | 193 | 7.8 | - | - | - | 1101 | 9 |
| 2 | Liaohe | 798 | Niuqibao | 123.28 | 42.30 | 10 | 2.2 | - | - | - | 1098 | 2 |
| 2 | Liaohe | 803 | Paoziyan | 123.55 | 42.51 | 10 | 6.1 | - | - | - | 1095 | 7 |
| 2 | Liaohe | 805 | Qinghe | 124.17 | 42.55 | 971 | 37.1 | - | - | - | 1073 | 40 |
| 2 | Liaohe | 791 | Qipanshan | 123.64 | 41.94 | 10 | 3.7 | - | - | - | 1093 | 4 |
| 2 | Liaohe | 822 | Rongxing | 122.00 | 40.80 | 10 | 7.4 | - | - | - | 1079 | 8 |
| 2 | Liaohe | 802 | Sanhecheng | 122.91 | 42.49 | 10 | 2.3 | - | - | - | 1090 | 3 |
| 2 | Liaohe | 807 | Santaizi | 123.35 | 42.63 | 30 | 15.0 | - | - | - | 1093 | 16 |
| 2 | Liaohe | 813 | Selihu | 120.59 | 42.76 | 120 | 21.6 | - | - | - | 1160 | 25 |
| 2 | Liaohe | 204 | Shandaohe | 123.94 | 41.49 | 30 | 3.0 | Sec | - | 1 | 1062 | 3 |
| 2 | Liaohe | 790 | Shangsi | 123.79 | 41.93 | 7 | 0.7 | - | - | - | 1088 | 0.76 |
| 2 | Liaohe | 800 | Shangtun | 123.36 | 42.38 | 10 | 8.9 | - | - | - | 1097 | 10 |
| 2 | Liaohe | 202 | Shangying | 122.93 | 40.86 | 32 | 2.3 | Sec | - | 93 | 1056 | 2 |
| 2 | Liaohe | 203 | Shenwo | 123.51 | 41.23 | 791 | 31.9 | Sec | - | 80 | 1059 | 34 |
| 2 | Liaohe | 768 | Shimen | 122.72 | 40.41 | 185 | 2.1 | - | - | - | 1035 | 2 |
| 2 | Liaohe | 777 | Tanghe | 123.37 | 41.11 | 723 | 15.3 | - | - | - | 1052 | 16 |
| 2 | Liaohe | 818 | Tuerjishan | 122.86 | 43.63 | 120 | 18.2 | - | - | - | 1087 | 20 |
| 2 | Liaohe | 198 | Tumenzi | 123.91 | 40.16 | 190 | 7.2 | Sec | 3 | 7 | 995 | 7 |
| 2 | Liaohe | 771 | Wangjiakan | 122.74 | 40.76 | 20 | 2.3 | - | - | - | 1058 | 2 |
| 2 | Liaohe | 811 | Wolonghu | 123.30 | 42.73 | 100 | 58.1 | - | - | - | 1091 | 63 |
| 2 | Liaohe | 774 | Wujintang | 120.73 | 41.04 | 318 | 4.5 | - | - | - | 1133 | 5 |
| 2 | Liaohe | 796 | Xiaogujia | 125.01 | 42.16 | 21 | 2.1 | - | - | - | 1045 | 2 |
| 2 | Liaohe | 819 | Xiaoshan | 123.90 | 43.82 | 105 | 2.9 | - | - | - | 1078 | 3 |
| 2 | Liaohe | 781 | Yaobao | 124.23 | 41.66 | 20 | 1.0 | - | - | - | 1061 | 1.06 |
| 2 | Liaohe | 780 | Yingshou | 123.90 | 41.66 | 12 | 1.0 | - | - | - | 1076 | 1.08 |
| 2 | Liaohe | 793 | Youling | 122.05 | 42.03 | 40 | 2.8 | - | - | - | 1110 | 3 |
| 2 | Liaohe | 794 | Zhenziling | 124.23 | 42.14 | 210 | 11.2 | - | - | - | 1059 | 12 |
| 3 | Haihe | 753 | Angezhuang | 115.23 | 39.28 | 309 | 17.2 | - | - | - | 1181 | 20 |
| 3 | Haihe | 745 | Baicaoping | 114.21 | 37.63 | 47 | 1.2 | - | - | - | 1188 | 1.43 |
| 3 | Haihe | 767 | Beitaishan | 116.67 | 40.39 | 38 | 1.5 | - | - | - | 1229 | 2 |
| 3 | Haihe | 756 | Cetian | 113.81 | 39.97 | 580 | 47.1 | - | - | - | 1120 | 53 |
| 3 | Haihe | 738 | Chegu | 113.83 | 36.84 | 37 | 0.4 | - | - | - | 1151 | 0.46 |
| 3 | Haihe | 763 | Daheiding | 118.30 | 40.20 | 337 | 19.3 | - | - | - | 1227 | 24 |
| 3 | Haihe | 732 | Dongsiwu | 114.30 | 36.40 | 181 | 11.8 | - | - | - | 1233 | 15 |
| 3 | Haihe | 192 | Gangnan | 114.00 | 38.32 | 1571 | 39.2 | Sec | 123 | 85 | 1171 | 46 |
| 3 | Haihe | 721 | Gongshang | 113.67 | 35.94 | 35 | 0.5 | - | - | - | 1163 | 0.58 |
| 3 | Haihe | 737 | Guanhe | 112.89 | 36.84 | 140 | 2.4 | - | - | - | 1152 | 3 |
| 3 | Haihe | 199 | Guanting | 115.60 | 40.23 | 2270 | 90.1 | Sec | 30 | 90 | 1111.3^a^ | 100 |
| 3 | Haihe | 762 | Haizi | 117.30 | 40.18 | 121 | 6.7 | - | - | - | 1219 | 8 |
| 3 | Haihe | 193 | Hengshanling | 114.18 | 38.57 | 243 | 7.0 | Sec | - | 2 | 1194 | 8 |
| 3 | Haihe | 735 | Houwuan | 112.82 | 36.56 | 146 | 4.9 | - | - | - | 1152 | 6 |
| 3 | Haihe | 766 | Huairou | 116.61 | 40.31 | 115 | 6.4 | - | - | - | 1228 | 8 |
| 3 | Haihe | 748 | Huangbizhuang | 114.30 | 38.25 | 1210 | 36.3 | - | 16 | 0 | 1239^a^ | 45 |
| 3 | Haihe | 194 | Koutou | 114.37 | 38.62 | 105 | 4.3 | Sec | 1 | 0 | 1211 | 5 |
| 3 | Haihe | 742 | Lincheng | 114.39 | 37.44 | 180 | 4.3 | - | - | - | 1007.9^a^ | 4 |
| 3 | Haihe | 752 | Longmen | 115.27 | 39.11 | 118 | 1.4 | - | - | - | 1197 | 2 |
| 3 | Haihe | 708 | Ma'amshi | 113.35 | 35.44 | 10 | 0.3 | - | - | - | 1184 | 0.36 |
| 3 | Haihe | 784 | Miaogong | 117.84 | 41.72 | 183 | 2.0 | - | - | - | 1113 | 2 |
| 3 | Haihe | 201 | Miyun | 116.84 | 40.49 | 4375 | 121.7 | Sec | 96 | 115 | 1316.1^a^ | 160 |
| 3 | Haihe | 728 | Nangudong | 113.75 | 36.23 | 83 | 0.9 | - | - | - | 1166 | 1.05 |
| 3 | Haihe | 200 | Panjiakou | 118.28 | 40.39 | 2930 | 18.0 | Sec | 270^b^ | 63^b^ | 1233 | 22 |
| 3 | Haihe | 757 | Qiuzhuang | 118.15 | 40.00 | 156 | 4.6 | - | - | - | 1000^a^ | 5 |
| 3 | Haihe | 759 | Shihe | 119.70 | 40.04 | 70 | 1.7 | - | - | - | 1158 | 2 |
| 3 | Haihe | 867 | Shisanling pump storage | 116.27 | 40.25 | 4 | 0.1 | - | 800^b^ | 404^b^ | 1208 | 0.12 |
| 3 | Haihe | 769 | Shuihutong | 118.96 | 40.47 | 40 | 0.9 | - | - | - | 1211 | 1.09 |
| 3 | Haihe | 761 | Taolinkou | 119.05 | 40.14 | 860 | 18.5 | - | 20 | 63 | 1019.1^a^ | 19 |
| 3 | Haihe | 730 | Tunjiang | 112.70 | 36.31 | 60 | 2.8 | - | - | - | 1145 | 3 |
| 3 | Haihe | 750 | Wangkuai | 114.51 | 38.74 | 1389 | 33.8 | - | - | 14 | 1265^a^ | 43 |
| 3 | Haihe | 195 | Xidayang | 114.78 | 38.75 | 1071 | 32.4 | Sec | 12 | 40 | 983.4^a^ | 32 |
| 3 | Haihe | 197 | Yanghe | 119.20 | 39.98 | 353 | 13.9 | Sec | 2 | 4 | 928.7^a^ | 13 |
| 3 | Haihe | 740 | Yegoumen | 114.10 | 37.20 | 50 | 0.6 | - | - | - | 1182 | 0.71 |
| 3 | Haihe | 772 | Youyi | 114.05 | 40.86 | 116 | 4.2 | - | - | - | 1100^a^ | 5 |
| 3 | Haihe | 188 | Yuecheng | 114.20 | 36.28 | 1090 | 15.2 | Sec | 17 | 45 | 1226 | 19 |
| 3 | Haihe | 775 | Yunzhou | 115.76 | 41.07 | 112 | 7.4 | - | - | - | 1134 | 8 |
| 3 | Haihe | 758 | Yuqiao | 117.44 | 40.03 | 1559 | 118.6 | - | - | - | 1229 | 146 |
| 3 | Haihe | 731 | Zhangzhe | 113.06 | 36.32 | 200 | 22.6 | - | - | - | 1149 | 26 |
| 3 | Haihe | 754 | Zhenziliang | 113.28 | 39.57 | 50 | 2.2 | - | - | - | 1123 | 2 |
| 3 | Haihe | 739 | Zhuzhuang | 114.19 | 37.01 | 436 | 5.6 | - | - | - | 1133.3^a^ | 6 |
| 4 | Yellow River | 186 | Baijiazui | 107.50 | 35.69 | 496 | 11.6 | Sec | 2 | 4 | 1000^a^ | 12 |
| 4 | Yellow River | 87 | Bapanxia | 103.40 | 36.14 | 50 | 4.9 | Main | 220^b^ | 1043^b^ | 969 | 5 |
| 4 | Yellow River | 741 | Changshantou | 105.69 | 37.33 | 57 | 30.5 | - | - | - | 1054 | 32 |
| 4 | Yellow River | 734 | Dananchuan | 101.60 | 36.49 | 13 | 0.4 | - | - | - | 1004 | 0.40 |
| 4 | Yellow River | 187 | Daxia | 104.02 | 36.19 | 90 | 2.8 | Sec | 300 | 1465 | 982 | 3 |
| 4 | Yellow River | 710 | Dongfeng | 112.20 | 35.48 | 23 | 0.4 | - | - | - | 1147 | 0.46 |
| 4 | Yellow River | 693 | Fengjiashan | 107.19 | 34.54 | 390 | 12.0 | - | - | 8 | 800^a^ | 10 |
| 4 | Yellow River | 191 | Fenhe | 111.91 | 38.05 | 733 | 21.1 | Sec | 13 | 11 | 1000^a^ | 21 |
| 4 | Yellow River | 747 | Fenhe 2 | 112.37 | 37.98 | 133 | 3.0 | - | - | - | 1119 | 3 |
| 4 | Yellow River | 692 | Ganhe | 108.43 | 34.52 | 54 | 2.0 | - | - | - | 1015 | 2 |
| 4 | Yellow River | 82 | Gongboxia | 102.23 | 35.88 | 692 | 17.0 | Main | 1500^b^ | 5905^b^ | 975 | 17 |
| 4 | Yellow River | 719 | Guangming | 117.60 | 35.88 | 100 | 9.4 | - | - | - | 1276 | 12 |
| 4 | Yellow River | 687 | Guxian | 111.28 | 34.24 | 1175 | 19.5 | - | 60 | 97 | 950^a^ | 19 |
| 4 | Yellow River | 749 | Hekou | 109.37 | 38.60 | 67 | 11.6 | - | - | - | 1129 | 13 |
| 4 | Yellow River | 181 | Jinpen | 108.20 | 34.05 | 200 | 2.2 | Sec | 20 | 73 | 968 | 2 |
| 4 | Yellow River | 712 | Kuaihe | 111.56 | 35.64 | 77 | 7.8 | - | - | - | 1152 | 9 |
| 4 | Yellow River | 85 | Laxiwa | 101.18 | 36.07 | 1080 | 11.8 | Main | 3500^b^ | 9667^b^ | 1100^a^ | 13 |
| 4 | Yellow River | 726 | Lijiaxia | 101.81 | 36.12 | 1650 | 31.9 | - | 1600^b^ | 6679^b^ | 979 | 31 |
| 4 | Yellow River | 83 | Liujiaxia | 103.34 | 35.93 | 6120 | 115.5 | Main | 570 | 5580 | 1000^a^ | 116 |
| 4 | Yellow River | 695 | Longbo | 111.65 | 34.61 | 53 | 3.6 | - | - | - | 1137 | 4 |
| 4 | Yellow River | 86 | Longyangxia | 100.92 | 36.12 | 27630 | 284.7 | Main | 1280^b^ | 6915^b^ | 1100^a^ | 313 |
| 4 | Yellow River | 182 | Luhun | 112.18 | 34.20 | 1320 | 57.3 | Sec | 10 | 10 | 989.9^a^ | 57 |
| 4 | Yellow River | 190 | Muyu | 120.74 | 37.06 | 227 | 2.8 | Sec | 5 | 3 | 1120 | 3 |
| 4 | Yellow River | 724 | Nina | 101.27 | 36.06 | 26 | 1.4 | - | - | - | 989 | 1.38 |
| 4 | Yellow River | 746 | Qingtongxia | 105.99 | 37.89 | 735 | 54.1 | - | 302^b^ | 1204^b^ | 1300^a^ | 70 |
| 4 | Yellow River | 723 | Quhe | 111.65 | 36.06 | 30 | 2.0 | - | - | - | 1158 | 2 |
| 4 | Yellow River | 711 | Renzhuang | 112.98 | 35.60 | 83 | 1.7 | - | - | - | 1161 | 2 |
| 4 | Yellow River | 184 | Sanmenxia | 111.34 | 34.83 | 9640 | 254.8 | Sec | 250 | 1310 | 1200^a^ | 306 |
| 4 | Yellow River | 765 | Sanshenggong | 107.03 | 40.30 | 80 | 30.1 | - | - | - | 1183 | 36 |
| 4 | Yellow River | 722 | Shagou | 118.63 | 36.05 | 102 | 4.5 | - | - | - | 1212 | 5 |
| 4 | Yellow River | 725 | Shenjiahe | 106.27 | 36.10 | 27 | 2.2 | - | - | - | 972 | 2 |
| 4 | Yellow River | 686 | Shitouhe | 107.65 | 34.17 | 147 | 1.9 | - | 50 | 51 | 800a | 2 |
| 4 | Yellow River | 736 | Shixiakou | 105.91 | 36.83 | 76 | 7.0 | - | - | - | 1300^a^ | 9 |
| 4 | Yellow River | 729 | Sikousi | 105.98 | 36.27 | 56 | 3.2 | - | - | - | 970 | 3 |
| 4 | Yellow River | 831 | Suzhi | 102.34 | 35.87 | 46 | 1.5 | - | 225^b^ | 960^b^ | 974 | 1.46 |
| 4 | Yellow River | 88 | Taihe | 118.13 | 36.54 | 183 | 6.1 | Main | 138 | 670 | 1275 | 8 |
| 4 | Yellow River | 751 | Tianqiao | 111.05 | 39.02 | 74 | 5.3 | - | - | - | 1132 | 6 |
| 4 | Yellow River | 690 | Wangjiawan | 107.25 | 34.43 | 21 | 7.4 | - | - | - | 940 | 7 |
| 4 | Yellow River | 89 | Wangyao | 109.09 | 36.90 | 203 | 9.7 | Main | 1 | 2 | 900^a^ | 9 |
| 4 | Yellow River | 196 | Wanjiazhai | 111.43 | 39.58 | 900 | 22.1 | Sec | 1080 | 2750 | 1120 | 25 |
| 4 | Yellow River | 743 | Wenyuhe | 112.02 | 37.52 | 110 | 2.2 | - | 3 | 8 | 900^a^ | 2 |
| 4 | Yellow River | 733 | Wohushan | 116.96 | 36.49 | 110 | 4.0 | - | - | - | 1304 | 5 |
| 4 | Yellow River | 185 | Xiaolangdi | 112.36 | 34.92 | 12650 | 61.6 | Sec | 1800^b^ | 5196^b^ | 1560^a^ | 96 |
| 4 | Yellow River | 864 | Xixiayuan | 112.52 | 34.88 | 162 | 18.7 | - | 140^b^ | 488^b^ | 1211 | 23 |
| 4 | Yellow River | 189 | Xueye | 117.58 | 36.41 | 221 | 6.1 | Sec | 1 | 2 | 1283 | 8 |
| 4 | Yellow River | 694 | Yangmaowan | 108.05 | 34.54 | 130 | 6.6 | - | - | - | 800^a^ | 5 |
| 4 | Yellow River | 84 | Yanguoxia | 103.27 | 36.06 | 280 | 17.3 | Main | 471.2^b^ | 2385^b^ | 1100^a^ | 19 |
| 4 | Yellow River | 691 | Youhe | 109.52 | 34.46 | 25 | 1.4 | - | - | - | 1059 | 1.48 |
| 4 | Yellow River | 183 | Zhaikou | 110.78 | 34.38 | 185 | 6.0 | Sec | 5 | 14 | 1000^a^ | 6 |
| 4 | Yellow River | 718 | Zhangjiazuitou | 105.85 | 35.83 | 34 | 3.5 | - | - | - | 951 | 3 |
| 4 | Yellow River | 716 | Zhejiahe | 109.63 | 35.76 | 30 | 2.1 | - | - | - | 1095 | 2 |
| 4 | Yellow River | 122 | Zhiganglaka | 101.88 | 36.11 | 15 | 0.7 | Main | 152^b^ | 704^b^ | 977 | 0.68 |
| 4 | Yellow River | 744 | Zinqiao | 108.44 | 37.52 | 200 | 11.1 | - | - | - | 1085 | 12 |
| 5 | Huaihe | 714 | Andi | 118.13 | 35.69 | 782 | 25.6 | - | - | - | 1251 | 32 |
| 5 | Huaihe | 689 | Anfengshan | 118.72 | 34.38 | 100 | 18.9 | - | - | - | 1172 | 22 |
| 5 | Huaihe | 180 | Baiguishan | 113.24 | 33.71 | 731 | 33.7 | Sec | 3 | 8 | 1176 | 40 |
| 5 | Huaihe | 688 | Baisha | 113.25 | 34.34 | 295 | 6.2 | - | 70^b^ | 224^b^ | 1181 | 7 |
| 5 | Huaihe | 677 | Banqiao | 113.63 | 32.98 | 675 | 37.5 | - | - | - | 1152 | 43 |
| 5 | Huaihe | 720 | Bashan | 118.53 | 35.90 | 509 | 15.7 | - | - | - | 1206 | 19 |
| 5 | Huaihe | 673 | Boshan | 113.94 | 32.65 | 620 | 9.2 | - | - | - | 1146 | 11 |
| 5 | Huaihe | 698 | Changzhuang | 113.56 | 34.72 | 17 | 0.6 | - | - | - | 1212 | 0.73 |
| 5 | Huaihe | 697 | Dindian | 113.38 | 34.71 | 59 | 0.5 | - | - | - | 1199 | 0.60 |
| 5 | Huaihe | 705 | Doushan | 118.85 | 35.33 | 300 | 13.4 | - | - | - | 1140 | 15 |
| 5 | Huaihe | 637 | Feishanhe | 113.83 | 31.85 | 82 | 1.9 | - | - | - | 1134 | 2 |
| 5 | Huaihe | 611 | Foziling | 116.27 | 31.35 | 496 | 12.2 | - | - | - | 1117 | 14 |
| 5 | Huaihe | 684 | Gushitan | 113.09 | 33.49 | 157 | 8.7 | - | - | - | 1160 | 10 |
| 5 | Huaihe | 649 | Heidongwan | 113.75 | 32.02 | 34 | 1.0 | - | - | - | 1130 | 1.13 |
| 5 | Huaihe | 713 | Hezhuang | 117.54 | 35.64 | 83 | 3.3 | - | - | - | 1270 | 4 |
| 5 | Huaihe | 643 | Huashan | 113.79 | 31.90 | 173 | 2.2 | - | - | - | 1132 | 2 |
| 5 | Huaihe | 700 | Huibaoling | 117.83 | 34.90 | 200 | 17.5 | - | - | - | 1237 | 22 |
| 5 | Huaihe | 696 | Jiangang | 113.57 | 34.69 | 68 | 1.2 | - | - | - | 1211 | 1.45 |
| 5 | Huaihe | 674 | Lindong | 118.13 | 32.66 | 10 | 3.1 | - | - | - | 1158 | 4 |
| 5 | Huaihe | 703 | Mahe | 117.21 | 35.21 | 138 | 7.8 | - | - | - | 1248 | 10 |
| 5 | Huaihe | 627 | Meishan | 115.88 | 31.67 | 2275 | 24.9 | - | 84^b^ | 413^b^ | 1115 | 28 |
| 5 | Huaihe | 600 | Mozitan | 116.35 | 31.24 | 337 | 4.3 | - | - | - | 1112 | 5 |
| 5 | Huaihe | 178 | Nanwan | 114.00 | 32.12 | 1630 | 39.3 | Sec | 6 | 15 | 1137 | 45 |
| 5 | Huaihe | 633 | Nianyushan | 115.36 | 31.79 | 916 | 19.1 | - | - | - | 1142 | 22 |
| 5 | Huaihe | 709 | Nishan | 117.19 | 35.48 | 119 | 7.6 | - | - | - | 1260 | 10 |
| 5 | Huaihe | 631 | Pohe | 114.92 | 31.79 | 214 | 11.4 | - | - | - | 1149 | 13 |
| 5 | Huaihe | 717 | Qingfengling | 118.86 | 35.80 | 410 | 19.0 | - | - | - | 1160 | 22 |
| 5 | Huaihe | 679 | Sanhezha | 118.73 | 33.09 | 13500 | 1374.3 | - | - | - | 1162 | 1597 |
| 5 | Huaihe | 699 | Shilianghe | 118.86 | 34.77 | 300 | 38.9 | - | 1 | 1 | 1682.9^a^ | 65 |
| 5 | Huaihe | 683 | Shimantan | 113.55 | 33.28 | 120 | 8.2 | - | - | - | 1163 | 10 |
| 5 | Huaihe | 650 | Shishankou | 114.39 | 32.02 | 372 | 42.4 | - | - | - | 1145 | 49 |
| 5 | Huaihe | 707 | Tangcun | 117.55 | 35.43 | 200 | 9.5 | - | - | - | 1254 | 12 |
| 5 | Huaihe | 727 | Tianzhuang | 118.11 | 36.17 | 136 | 7.9 | - | - | - | 1262 | 10 |
| 5 | Huaihe | 667 | Wangtun | 113.98 | 32.37 | 3 | 1.2 | - | - | - | 1143 | 1.37 |
| 5 | Huaihe | 638 | Wuyue | 114.65 | 31.86 | 118 | 7.7 | - | - | - | 1147 | 9 |
| 5 | Huaihe | 623 | Xianghongdian | 116.15 | 31.56 | 2632 | 38.2 | - | 80 | 132 | 1127 | 43 |
| 5 | Huaihe | 625 | Xiangshan | 114.90 | 31.59 | 84 | 4.0 | - | - | - | 1151 | 5 |
| 5 | Huaihe | 715 | Xiaoshiyang | 118.98 | 35.74 | 125 | 8.2 | - | - | - | 1136 | 9 |
| 5 | Huaihe | 701 | Xiaotashan | 118.96 | 34.94 | 300 | 13.3 | - | - | - | 1145 | 15 |
| 5 | Huaihe | 706 | Xiwei | 117.03 | 35.40 | 107 | 5.1 | - | - | - | 1259 | 6 |
| 5 | Huaihe | 702 | Xujiaya | 117.87 | 35.20 | 292 | 9.1 | - | - | - | 1238 | 11 |
| 5 | Huaihe | 676 | Xuyahu | 114.28 | 32.96 | 1656 | 77.8 | - | - | - | 1175 | 91 |
| 5 | Huaihe | 704 | Yanma | 117.27 | 35.22 | 104 | 1.9 | - | - | - | 1246 | 2 |
| 5 | Huaihe | 685 | Zhaopingtai | 112.77 | 33.73 | 727 | 25.3 | - | - | - | 1150 | 29 |
| 6 | Yangtze River | 78 | Ankang | 108.88 | 32.60 | 2580 | 57.3 | Main | 800 | 2857 | 748.4^a^ | 43 |
| 6 | Yangtze River | 594 | Baertan | 106.04 | 31.16 | 17 | 1.3 | - | - | - | 947 | 1.23 |
| 6 | Yangtze River | 535 | Baihe | 106.08 | 30.24 | 25 | 2.6 | - | - | - | 949 | 2 |
| 6 | Yangtze River | 349 | Baihua | 106.54 | 26.69 | 221 | 11.8 | - | - | - | 969 | 11 |
| 6 | Yangtze River | 365 | Bailian | 113.03 | 27.22 | 33 | 2.0 | - | - | - | 1067 | 2 |
| 6 | Yangtze River | 70 | Bailianhe | 115.45 | 30.59 | 1250 | 32.8 | Main | 1200^b^ | 84^b^ | 1135 | 37 |
| 6 | Yangtze River | 593 | Bailin | 106.97 | 31.15 | 21 | 3.2 | - | - | - | 950 | 3 |
| 6 | Yangtze River | 487 | Bailinsi | 105.32 | 29.40 | 10 | 0.6 | - | - | - | 972 | 0.58 |
| 6 | Yangtze River | 399 | Baimiao | 117.12 | 28.09 | 19 | 0.4 | - | - | - | 1175 | 0.47 |
| 6 | Yangtze River | 621 | Baishuihe | 104.27 | 31.53 | 17 | 2.1 | - | - | - | 950 | 2 |
| 6 | Yangtze River | 32 | Baiyun | 110.33 | 26.33 | 545 | 7.6 | Main | 54 | 117 | 948 | 7 |
| 6 | Yangtze River | 41 | Baiyunshan 1 | 115.32 | 26.81 | 114 | 1.3 | Main | 21 | 67 | 1171 | 2 |
| 6 | Yangtze River | 38 | Baiyutan | 112.87 | 26.58 | 300 | 21.9 | Main | 22 | 890 | 1086 | 24 |
| 6 | Yangtze River | 532 | Baizhang | 103.23 | 30.19 | 21 | 1.8 | - | - | - | 944 | 2 |
| 6 | Yangtze River | 379 | Banshan | 111.10 | 27.62 | 10 | 0.2 | - | - | - | 995 | 0.20 |
| 6 | Yangtze River | 587 | Baoshi | 107.98 | 31.06 | 68 | 3.7 | - | - | - | 985 | 4 |
| 6 | Yangtze River | 298 | Baoxianghe | 102.93 | 25.04 | 21 | 1.3 | - | - | - | 1173 | 2 |
| 6 | Yangtze River | 834 | Baoxing | 102.81 | 30.36 | 1 | 0.0 | - | 195^b^ | 827^b^ | 953 | 0.02 |
| 6 | Yangtze River | 76 | Baozhusi | 105.61 | 32.52 | 2550 | 66.3 | Main | 700^b^ | 1200^b^ | 954 | 63 |
| 6 | Yangtze River | 590 | Bazimen | 113.14 | 31.11 | 101 | 2.3 | - | - | - | 1085 | 2 |
| 6 | Yangtze River | 528 | Beihe | 111.59 | 30.14 | 56 | 3.6 | - | - | - | 1033 | 4 |
| 6 | Yangtze River | 598 | Beishan 1 | 112.41 | 31.20 | 38 | 1.1 | - | - | - | 1084 | 1.19 |
| 6 | Yangtze River | 656 | Beishan 2 | 119.18 | 32.08 | 100 | 3.1 | - | - | - | 1120 | 3 |
| 6 | Yangtze River | 80 | Bikou | 105.22 | 32.76 | 521 | 7.2 | Main | 300 | 1463 | 966 | 7 |
| 6 | Yangtze River | 470 | Bintian | 116.90 | 29.21 | 115 | 8.3 | - | - | - | 1199 | 10 |
| 6 | Yangtze River | 438 | Cangshan | 111.63 | 28.67 | 100 | 4.2 | - | - | - | 1020 | 4 |
| 6 | Yangtze River | 304 | Centianhe | 111.67 | 25.15 | 105 | 2.2 | - | - | - | 1082 | 2 |
| 6 | Yangtze River | 432 | Chaijiaowan | 117.42 | 28.55 | 30 | 2.0 | - | - | - | 1188 | 2 |
| 6 | Yangtze River | 341 | Changgang | 115.45 | 26.33 | 365 | 21.7 | - | - | - | 1190 | 26 |
| 6 | Yangtze River | 505 | Changshaba | 104.65 | 29.71 | 45 | 3.1 | - | - | - | 946 | 3 |
| 6 | Yangtze River | 392 | Changtangang | 109.70 | 27.88 | 100 | 4.0 | - | - | - | 1015 | 4 |
| 6 | Yangtze River | 384 | Changtianwan | 110.20 | 27.76 | 23 | 1.0 | - | - | - | 1006 | 1.01 |
| 6 | Yangtze River | 175 | Cheba 1 | 109.30 | 30.29 | 62 | 2.1 | Sec | 20 | 85 | 979 | 2 |
| 6 | Yangtze River | 548 | Chencun | 118.18 | 30.48 | 2641 | 57.5 | - | 184^b^ | 355^b^ | 1060 | 61 |
| 6 | Yangtze River | 390 | Chetianjiang | 111.60 | 27.86 | 138 | 7.5 | - | - | - | 1006 | 8 |
| 6 | Yangtze River | 143 | Chongjianghe | 99.80 | 27.55 | 0 | 0.0 | Main | 70.3^b^ | 193^b^ | 1015.8^a^ | 0.01 |
| 6 | Yangtze River | 529 | Chongkan | 105.61 | 30.15 | 100 | 18.6 | - | - | - | 939 | 17 |
| 6 | Yangtze River | 554 | Cuntangkou | 105.18 | 30.55 | 14 | 2.3 | - | - | - | 924 | 2 |
| 6 | Yangtze River | 53 | Daduan | 114.55 | 28.65 | 115 | 7.2 | Main | 13 | 43 | 1102 | 8 |
| 6 | Yangtze River | 436 | Dagangqiao | 117.14 | 28.64 | 48 | 1.6 | - | - | - | 1197 | 2 |
| 6 | Yangtze River | 557 | Dagaotan | 106.28 | 30.61 | 38 | 1.2 | - | - | - | 950 | 1.14 |
| 6 | Yangtze River | 565 | Dahekou | 119.52 | 30.75 | 10 | 0.4 | - | - | - | 1082 | 0.43 |
| 6 | Yangtze River | 68 | Dahonghe | 106.96 | 30.01 | 368 | 13.0 | Main | 2 | 11 | 965 | 13 |
| 6 | Yangtze River | 177 | Dahongshan | 112.94 | 31.65 | 126 | 11.0 | Sec | 2 | 447 | 1114 | 12 |
| 6 | Yangtze River | 126 | Dahua | 107.26 | 26.82 | 218 | 7.7 | Main | 200^b^ | 611^b^ | 954 | 7 |
| 6 | Yangtze River | 454 | Dajiangdong | 113.78 | 28.90 | 36 | 0.8 | - | - | - | 1079 | 0.86 |
| 6 | Yangtze River | 846 | Dajinping | 102.34 | 29.23 | 1 | 0.1 | - | 129^b^ | 606^b^ | 986 | 0.10 |
| 6 | Yangtze River | 77 | Danjiangkou | 111.49 | 32.56 | 20890 | 286.3 | Main | 900 | 3830 | 1000^a^ | 286 |
| 6 | Yangtze River | 575 | Daoguanhe | 114.99 | 30.88 | 110 | 5.0 | - | - | - | 1163 | 6 |
| 6 | Yangtze River | 439 | Daqiao | 102.20 | 28.67 | 700 | 24.5 | - | - | - | 1025 | 25 |
| 6 | Yangtze River | 417 | Dashan | 116.77 | 28.39 | 20 | 2.0 | - | - | - | 1186 | 2 |
| 6 | Yangtze River | 567 | Dashiqiao | 105.69 | 30.79 | 35 | 6.4 | - | - | - | 939 | 6 |
| 6 | Yangtze River | 350 | Dashuijiang | 110.88 | 26.75 | 33 | 1.1 | - | - | - | 1048 | 1.15 |
| 6 | Yangtze River | 549 | Datong | 115.79 | 30.50 | 270 | 8.6 | - | - | - | 1115 | 10 |
| 6 | Yangtze River | 614 | Daxi | 119.36 | 31.39 | 10 | 11.1 | - | - | - | 1101 | 12 |
| 6 | Yangtze River | 861 | Daxing | 103.00 | 29.99 | 19 | 0.6 | - | 75^b^ | 373^b^ | 940 | 0.56 |
| 6 | Yangtze River | 342 | Dazhen | 110.63 | 26.44 | 71 | 1.6 | - | - | - | 992 | 2 |
| 6 | Yangtze River | 541 | Diaoyutai | 116.00 | 30.35 | 94 | 2.5 | - | - | - | 1122 | 3 |
| 6 | Yangtze River | 353 | Dongfeng | 106.15 | 26.86 | 1020 | 20.9 | - | 695^b^ | 1632^b^ | 963 | 20 |
| 6 | Yangtze River | 27 | Dongjiang | 113.31 | 25.87 | 9200 | 130.6 | Main | 500 | 1320 | 1086 | 142 |
| 6 | Yangtze River | 58 | Dongjin | 114.33 | 28.98 | 800 | 25.3 | Main | 60 | 116 | 1108 | 28 |
| 6 | Yangtze River | 873 | Dongping | 111.20 | 28.37 | 20 | 0.6 | - | 72^b^ | 239^b^ | 1003 | 0.60 |
| 6 | Yangtze River | 642 | Dongpu | 117.20 | 31.89 | 200 | 13.9 | - | - | - | 1167 | 16 |
| 6 | Yangtze River | 837 | Dongxiguan | 106.13 | 30.48 | 165 | 5.8 | - | 180^b^ | 898^b^ | 948 | 5 |
| 6 | Yangtze River | 141 | Doulingzi | 110.02 | 32.88 | 484 | 17.8 | Main | 70.5^b^ | 243^b^ | 1021 | 18 |
| 6 | Yangtze River | 495 | Duanshen | 118.01 | 29.49 | 100 | 2.1 | - | - | - | 1106 | 2 |
| 6 | Yangtze River | 551 | Duihekou | 119.89 | 30.53 | 116 | 5.6 | - | - | - | 1087 | 6 |
| 6 | Yangtze River | 426 | Duitouling | 117.48 | 28.51 | 16 | 0.4 | - | - | - | 1185 | 0 |
| 6 | Yangtze River | 640 | Ershengqiao | 119.19 | 31.87 | 100 | 5.8 | - | - | - | 1114 | 6 |
| 6 | Yangtze River | 42 | Ertan | 101.78 | 26.82 | 6200 | 65.5 | Main | 3300^b^ | 15786^b^ | 1270^a^ | 83 |
| 6 | Yangtze River | 628 | Fangbian | 119.12 | 31.71 | 10 | 5.2 | - | - | - | 1114 | 6 |
| 6 | Yangtze River | 47 | Fangtuan | 117.39 | 28.15 | 31 | 2.2 | Main | - | 85 | 1180 | 3 |
| 6 | Yangtze River | 395 | Feijiantan | 114.11 | 27.92 | 114 | 10.4 | - | - | - | 1088 | 11 |
| 6 | Yangtze River | 43 | Fengdu | 114.08 | 26.93 | 100 | 2.1 | Main | 9 | 40 | 1068 | 2 |
| 6 | Yangtze River | 648 | Fengjiangkou | 113.34 | 31.98 | 202 | 8.7 | - | - | - | 1117 | 10 |
| 6 | Yangtze River | 346 | Fengling | 115.00 | 26.59 | 25 | 2.0 | - | - | - | 1174 | 2 |
| 6 | Yangtze River | 430 | Fenglinwan | 117.25 | 28.53 | 30 | 2.1 | - | - | - | 1196 | 3 |
| 6 | Yangtze River | 55 | Fengtan | 110.27 | 28.72 | 1544 | 15.6 | Main | 400 | 2043 | 1003 | 16 |
| 6 | Yangtze River | 363 | Fuhuashan | 114.75 | 27.15 | 31 | 4.2 | - | - | - | 1155 | 5 |
| 6 | Yangtze River | 359 | Fukuo | 105.98 | 27.09 | 100 | 5.1 | - | - | - | 939 | 5 |
| 6 | Yangtze River | 596 | Fuqiaohe | 114.88 | 31.17 | 580 | 20.7 | - | - | - | 1162 | 24 |
| 6 | Yangtze River | 559 | Fushi | 119.50 | 30.63 | 218 | 10.2 | - | - | - | 1074 | 11 |
| 6 | Yangtze River | 171 | Fushui | 114.88 | 29.69 | 1730 | 56.8 | Sec | 34 | 120 | 1136 | 65 |
| 6 | Yangtze River | 393 | Ganghe | 115.99 | 27.88 | 30 | 2.3 | - | - | - | 1166 | 3 |
| 6 | Yangtze River | 162 | Gaofang | 116.85 | 27.94 | 62 | 4.7 | Sec | 7 | 3 | 1159 | 5 |
| 6 | Yangtze River | 602 | Gaoguang | 113.19 | 31.27 | 212 | 15.1 | - | - | - | 1088 | 16 |
| 6 | Yangtze River | 156 | Gaoyan | 111.15 | 26.45 | 50 | 2.1 | Sec | - | 12 | 1049 | 2 |
| 6 | Yangtze River | 69 | Geheyan | 111.14 | 30.47 | 3400 | 41.0 | Main | 1511 | 3040 | 994 | 41 |
| 6 | Yangtze River | 847 | Geliqiao | 107.20 | 27.37 | 70 | 2.3 | - | 150^b^ | 414^b^ | 953 | 2 |
| 6 | Yangtze River | 71 | Gezhouba | 111.26 | 30.74 | 1580 | 53.2 | Main | 2735^b^ | 16241^b^ | 753.4^a^ | 40 |
| 6 | Yangtze River | 466 | Gongchanzhuyi | 117.38 | 29.14 | 200 | 7.3 | - | - | - | 1177 | 9 |
| 6 | Yangtze River | 580 | Gonghe | 111.72 | 30.96 | 173 | 13.1 | - | - | - | 1054 | 14 |
| 6 | Yangtze River | 62 | Gongzui | 103.48 | 29.31 | 400 | 12.0 | Main | 1330^b^ | 6397^b^ | 919 | 11 |
| 6 | Yangtze River | 102 | Goupitan | 107.65 | 27.37 | 5564 | 74.9 | Main | 3000^b^ | 5584^b^ | 930 | 70 |
| 6 | Yangtze River | 571 | Guandaohe | 111.66 | 30.83 | 16 | 0.3 | - | - | - | 1051 | 0.32 |
| 6 | Yangtze River | 163 | Guanli | 118.20 | 28.30 | 28 | 0.8 | Sec | - | 134 | 1174 | 0.94 |
| 6 | Yangtze River | 672 | Guanshan | 110.96 | 32.50 | 23 | 0.5 | - | - | - | 1064 | 0.53 |
| 6 | Yangtze River | 508 | Guanting | 111.54 | 29.71 | 10 | 4.2 | - | - | - | 1041 | 4 |
| 6 | Yangtze River | 364 | Guanxi | 114.91 | 27.19 | 20 | 2.3 | - | - | - | 1169 | 3 |
| 6 | Yangtze River | 610 | Guanyinyan | 114.13 | 31.34 | 100 | 2.6 | - | - | - | 1137 | 3 |
| 6 | Yangtze River | 394 | Guanzhuang 1 | 113.43 | 27.88 | 128 | 7.4 | - | - | - | 1091 | 8 |
| 6 | Yangtze River | 568 | Guanzhuang 2 | 111.43 | 30.80 | 16 | 0.3 | - | - | - | 1044 | 0.31 |
| 6 | Yangtze River | 849 | Gucheng | 104.62 | 32.38 | 3 | 0.1 | - | 112^b^ | 51^b^ | 958 | 0.10 |
| 6 | Yangtze River | 74 | Gudongkou | 110.75 | 31.37 | 138 | 3.0 | Main | 45 | 122 | 1031 | 3 |
| 6 | Yangtze River | 368 | Gukou | 114.55 | 27.29 | 33 | 1.1 | - | - | - | 1128 | 1.24 |
| 6 | Yangtze River | 581 | Guniubei | 116.84 | 31.02 | 77 | 2.2 | - | - | - | 1132 | 2 |
| 6 | Yangtze River | 479 | Guyumiao | 105.23 | 29.33 | 56 | 5.6 | - | - | - | 974 | 5 |
| 6 | Yangtze River | 325 | Haishao | 100.65 | 25.69 | 100 | 5.8 | - | - | - | 1204 | 7 |
| 6 | Yangtze River | 429 | Haojiacun | 104.63 | 28.52 | 18 | 2.3 | - | - | - | 981 | 2 |
| 6 | Yangtze River | 525 | Heilongtan | 104.05 | 30.04 | 356 | 30.3 | - | - | - | 931 | 28 |
| 6 | Yangtze River | 639 | Heiwuwan | 113.13 | 31.87 | 179 | 3.4 | - | - | - | 1123 | 4 |
| 6 | Yangtze River | 414 | Hengshan 1 | 116.68 | 28.33 | 30 | 2.1 | - | - | - | 1181 | 2 |
| 6 | Yangtze River | 601 | Hengshan 2 | 119.56 | 31.24 | 10 | 4.6 | - | - | - | 1091 | 5 |
| 6 | Yangtze River | 566 | Heping | 119.91 | 30.76 | 12 | 0.4 | - | - | - | 1088 | 0.44 |
| 6 | Yangtze River | 406 | Hexi | 109.81 | 28.22 | 52 | 4.3 | - | - | - | 997 | 4 |
| 6 | Yangtze River | 37 | Hongfeng | 106.43 | 26.53 | 660 | 43.4 | Main | 267^b^ | 585^b^ | 993 | 43 |
| 6 | Yangtze River | 404 | Honghu | 116.91 | 28.20 | 13 | 2.1 | - | - | - | 1180 | 2 |
| 6 | Yangtze River | 133 | Hongjiang | 109.96 | 27.13 | 320 | 11.6 | Main | 270^b^ | 793^b^ | 1017 | 12 |
| 6 | Yangtze River | 160 | Hongmen | 116.72 | 27.48 | 1230 | 34.9 | Sec | 42 | 120 | 1170 | 41 |
| 6 | Yangtze River | 352 | Hongqi | 112.15 | 26.83 | 21 | 2.0 | - | - | - | 1064 | 2 |
| 6 | Yangtze River | 607 | Hongqiyan | 105.16 | 31.34 | 32 | 2.4 | - | - | - | 941 | 2 |
| 6 | Yangtze River | 664 | Hongshuihe | 111.86 | 32.28 | 200 | 6.7 | - | - | - | 1095 | 7 |
| 6 | Yangtze River | 354 | Hongyan | 106.42 | 26.87 | 30 | 0.9 | - | - | - | 957 | 0.86 |
| 6 | Yangtze River | 868 | Hongye 2 | 103.37 | 31.57 | 0 | 0.0 | - | 90^b^ | 385^b^ | 970 | 0.004 |
| 6 | Yangtze River | 134 | Hongyi | 102.33 | 29.24 | 0 | 0.0 | Main | 80^b^ | 369^b^ | 986 | 0.003 |
| 6 | Yangtze River | 644 | Huacheng | 106.90 | 31.91 | 63 | 6.7 | - | - | - | 938 | 6 |
| 6 | Yangtze River | 547 | Hualiangting | 116.25 | 30.47 | 2408 | 47.8 | - | - | - | 1109 | 53 |
| 6 | Yangtze River | 511 | Hualong | 105.76 | 29.77 | 22 | 1.1 | - | - | - | 957 | 1.05 |
| 6 | Yangtze River | 23 | Huamuqiao | 113.80 | 25.69 | 31 | 2.0 | Main | 54 | 143 | 1093 | 2 |
| 6 | Yangtze River | 48 | Huangcai | 112.08 | 28.16 | 149 | 8.9 | Main | 5 | 13 | 1019 | 9 |
| 6 | Yangtze River | 497 | Huanghezhen | 104.91 | 29.53 | 13 | 3.5 | - | - | - | 961 | 3 |
| 6 | Yangtze River | 361 | Huangjiaba | 111.62 | 27.10 | 10 | 2.0 | - | - | - | 1051 | 2 |
| 6 | Yangtze River | 662 | Huanglishu | 118.10 | 32.20 | 299 | 12.8 | - | - | - | 1150 | 15 |
| 6 | Yangtze River | 79 | Huanglongtan | 110.52 | 32.68 | 1163 | 12.7 | Main | 150 | 759 | 1049 | 13 |
| 6 | Yangtze River | 615 | Huangpo | 112.66 | 31.45 | 119 | 14.6 | - | - | - | 1097 | 16 |
| 6 | Yangtze River | 469 | Huangshi | 111.18 | 29.20 | 612 | 15.5 | - | - | - | 1015 | 16 |
| 6 | Yangtze River | 282 | Huangyun | 114.35 | 24.65 | 50 | 2.4 | - | - | - | 1197 | 3 |
| 6 | Yangtze River | 425 | Huangzhuang | 116.93 | 28.49 | 13 | 0.5 | - | - | - | 1193 | 0.60 |
| 6 | Yangtze River | 343 | Huaxi | 106.64 | 26.44 | 26 | 2.1 | - | - | - | 987 | 2 |
| 6 | Yangtze River | 653 | Huayanghe | 112.92 | 32.07 | 123 | 6.8 | - | - | - | 1128 | 8 |
| 6 | Yangtze River | 546 | Huayuan | 115.66 | 30.45 | 108 | 3.7 | - | - | - | 1127 | 4 |
| 6 | Yangtze River | 563 | Huilong | 106.55 | 30.72 | 20 | 2.6 | - | 120^b^ | 125^b^ | 951 | 2 |
| 6 | Yangtze River | 582 | Huiting | 113.08 | 31.02 | 314 | 18.1 | - | - | - | 1084 | 20 |
| 6 | Yangtze River | 502 | Hulukou | 104.61 | 29.60 | 76 | 2.6 | - | - | - | 950 | 2 |
| 6 | Yangtze River | 661 | Huohe | 110.24 | 32.18 | 109 | 4.0 | - | - | - | 1023 | 4 |
| 6 | Yangtze River | 374 | Jiangkou 1 | 114.81 | 27.41 | 22 | 2.5 | - | 300^b^ | 1004^b^ | 1161 | 3 |
| 6 | Yangtze River | 383 | Jiangkou 2 | 114.83 | 27.73 | 890 | 32.5 | - | - | - | 1154 | 38 |
| 6 | Yangtze River | 170 | Jiangya | 110.74 | 29.54 | 1741 | 26.5 | Sec | 300 | 756 | 989 | 26 |
| 6 | Yangtze River | 36 | Jiaoyuan | 114.88 | 26.49 | 30 | 2.0 | Main | 1 | 81 | 1166 | 2 |
| 6 | Yangtze River | 573 | Jiguang | 104.84 | 30.85 | 98 | 5.7 | - | - | - | 924 | 5 |
| 6 | Yangtze River | 427 | Jinbei | 117.06 | 28.51 | 40 | 2.1 | - | - | - | 1195 | 3 |
| 6 | Yangtze River | 357 | Jinjiang 1 | 111.42 | 26.88 | 15 | 1.0 | - | - | - | 1059 | 1.06 |
| 6 | Yangtze River | 518 | Jinjiang 2 | 112.29 | 29.90 | 15 | 13.5 | - | - | - | 1054 | 14 |
| 6 | Yangtze River | 670 | Jinniushan | 118.97 | 32.47 | 100 | 8.9 | - | - | - | 1132 | 10 |
| 6 | Yangtze River | 603 | Jinshahe | 114.59 | 31.29 | 179 | 10.5 | - | - | - | 1155 | 12 |
| 6 | Yangtze River | 533 | Jiudaohe | 111.47 | 30.20 | 14 | 0.4 | - | - | - | 1027 | 0.41 |
| 6 | Yangtze River | 158 | Jiufujiang | 113.57 | 27.20 | 300 | 13.2 | Sec | 9 | 31 | 1069 | 14 |
| 6 | Yangtze River | 307 | Jiulongdian | 101.41 | 25.22 | 100 | 3.1 | - | - | - | 1208 | 4 |
| 6 | Yangtze River | 500 | Junmin | 116.91 | 29.59 | 216 | 7.5 | - | - | - | 1160 | 9 |
| 6 | Yangtze River | 50 | Juntan | 118.27 | 28.26 | 53 | 3.2 | Main | 9 | 26 | 1170 | 4 |
| 6 | Yangtze River | 647 | Jurong | 119.20 | 31.97 | 30 | 3.0 | - | - | - | 1117 | 3 |
| 6 | Yangtze River | 435 | Kapeng | 109.28 | 28.64 | 27 | 0.4 | - | - | - | 969 | 0.39 |
| 6 | Yangtze River | 552 | Laoshikan | 119.47 | 30.54 | 115 | 5.6 | - | - | - | 1069 | 6 |
| 6 | Yangtze River | 347 | Laoyingpan | 115.14 | 26.60 | 107 | 7.4 | - | - | - | 1176 | 9 |
| 6 | Yangtze River | 174 | Laoyingyan | 104.52 | 30.19 | 37 | 3.0 | Sec | 500 | 2340 | 917 | 3 |
| 6 | Yangtze River | 833 | Lengzhuguan | 101.96 | 30.05 | 1 | 0.0 | - | 180^b^ | 984^b^ | 974 | 0.03 |
| 6 | Yangtze River | 127 | Lianbu | 102.81 | 27.70 | 2 | 0.1 | Main | 130^b^ | 594^b^ | 1066 | 0.11 |
| 6 | Yangtze River | 173 | Liangcha | 106.79 | 29.94 | 37 | 3.4 | Sec | 3 | 9 | 965 | 3 |
| 6 | Yangtze River | 387 | Liangwan | 109.28 | 27.81 | 10 | 0.2 | - | - | - | 999 | 0.20 |
| 6 | Yangtze River | 366 | Liaoyuan | 116.91 | 27.24 | 31 | 4.1 | - | - | - | 1170 | 5 |
| 6 | Yangtze River | 290 | Liheng | 115.00 | 24.80 | 39 | 1.9 | - | - | - | 1229 | 2 |
| 6 | Yangtze River | 120 | Linjintan | 111.21 | 28.76 | 634 | 23.6 | Main | 270^b^ | 1162^b^ | 1019 | 24 |
| 6 | Yangtze River | 655 | Lintan | 119.39 | 32.07 | 15 | 1.6 | - | - | - | 1116 | 2 |
| 6 | Yangtze River | 372 | Liuduzhai | 110.92 | 27.37 | 121 | 5.9 | - | - | - | 1004 | 6 |
| 6 | Yangtze River | 45 | Liujiaping | 110.64 | 27.49 | 33 | 2.8 | Main | 17 | 54 | 994 | 3 |
| 6 | Yangtze River | 844 | Liuping | 103.82 | 31.66 | 1 | 0.1 | - | 120^b^ | 540^b^ | 971 | 0.10 |
| 6 | Yangtze River | 369 | Lixikou | 109.47 | 27.31 | 32 | 2.3 | - | - | - | 1014 | 2 |
| 6 | Yangtze River | 852 | Lizipin | 102.37 | 29.22 | 276 | 9.9 | - | 132^b^ | 312^b^ | 985 | 10 |
| 6 | Yangtze River | 604 | Longhekou | 116.76 | 31.31 | 848 | 39.0 | - | - | - | 1128 | 44 |
| 6 | Yangtze River | 520 | Longjiang | 105.02 | 29.92 | 24 | 4.8 | - | - | - | 943 | 5 |
| 6 | Yangtze River | 351 | Longjiangqiao | 111.63 | 26.78 | 13 | 0.5 | - | - | - | 1066 | 0.53 |
| 6 | Yangtze River | 314 | Longjin | 115.16 | 25.31 | 15 | 0.3 | - | - | - | 1221 | 0.37 |
| 6 | Yangtze River | 534 | Longping | 115.98 | 30.23 | 132 | 2.2 | - | - | - | 1133 | 2 |
| 6 | Yangtze River | 329 | Longshan 1 | 116.08 | 26.01 | 30 | 2.5 | - | - | - | 1207 | 3 |
| 6 | Yangtze River | 654 | Longshan 2 | 119.25 | 32.07 | 28 | 1.9 | - | - | - | 1117 | 2 |
| 6 | Yangtze River | 498 | Longshuihu | 105.80 | 29.54 | 16 | 1.7 | - | - | - | 965 | 2 |
| 6 | Yangtze River | 340 | Longtan | 113.31 | 26.31 | 60 | 5.0 | - | - | 3 | 1105 | 6 |
| 6 | Yangtze River | 284 | Longxing | 114.52 | 24.70 | 24 | 0.4 | - | - | - | 1199 | 0.48 |
| 6 | Yangtze River | 355 | Longxiqiao | 112.98 | 26.87 | 40 | 2.5 | - | - | - | 1080 | 3 |
| 6 | Yangtze River | 576 | Luban | 105.00 | 30.89 | 294 | 19.3 | - | - | - | 926 | 18 |
| 6 | Yangtze River | 569 | Lucun | 119.43 | 30.81 | 72 | 2.2 | - | - | - | 1081 | 2 |
| 6 | Yangtze River | 128 | Lugu | 103.04 | 27.58 | 37 | 1.2 | Main | 110^b^ | 300^b^ | 1057 | 1.27 |
| 6 | Yangtze River | 870 | Luosiwan | 99.74 | 27.83 | 150 | 5.2 | - | 60^b^ | 236^b^ | 1015.8^a^ | 5 |
| 6 | Yangtze River | 457 | Luowan | 115.13 | 28.99 | 77 | 1.7 | - | - | - | 1132 | 2 |
| 6 | Yangtze River | 380 | Luozishan | 110.44 | 27.68 | 18 | 0.2 | - | - | - | 996 | 0.20 |
| 6 | Yangtze River | 65 | Lushui | 113.89 | 29.68 | 706 | 33.0 | Main | 240 | 12740 | 936.1^a^ | 31 |
| 6 | Yangtze River | 421 | Mahu | 103.79 | 28.43 | 32 | 9.2 | - | - | - | 899 | 8 |
| 6 | Yangtze River | 396 | Majie | 116.83 | 27.92 | 40 | 3.1 | - | - | - | 1159 | 4 |
| 6 | Yangtze River | 144 | Majitang | 111.78 | 28.69 | 88 | 7.3 | Main | 55.5^b^ | 193^b^ | 1026 | 7 |
| 6 | Yangtze River | 333 | Malishu | 101.67 | 26.12 | 23 | 2.4 | - | - | - | 1247 | 3 |
| 6 | Yangtze River | 442 | Mamiao | 105.31 | 28.71 | 15 | 1.8 | - | - | - | 970 | 2 |
| 6 | Yangtze River | 510 | Maoba | 103.96 | 29.76 | 16 | 2.7 | - | - | - | 952 | 3 |
| 6 | Yangtze River | 33 | Maojiacun | 103.27 | 26.35 | 553 | 14.4 | Main | 16 | 73 | 1132 | 16 |
| 6 | Yangtze River | 73 | Maojianshan | 116.40 | 30.83 | 54 | 2.0 | Main | 25 | 40 | 1094 | 2 |
| 6 | Yangtze River | 636 | Maoshan | 119.25 | 31.82 | 24 | 2.7 | - | - | - | 1111 | 3 |
| 6 | Yangtze River | 472 | Maoxi | 110.32 | 29.22 | 60 | 2.3 | - | - | - | 996 | 2 |
| 6 | Yangtze River | 875 | Mayandong | 108.17 | 29.75 | 30 | 0.9 | - | 66^b^ | 200^b^ | 930 | 0.84 |
| 6 | Yangtze River | 378 | Mayuan | 116.58 | 27.57 | 30 | 2.1 | - | - | - | 1160 | 2 |
| 6 | Yangtze River | 543 | Mazitan | 105.25 | 30.42 | 77 | 4.9 | - | - | - | 926 | 5 |
| 6 | Yangtze River | 530 | Meichuan | 115.62 | 30.16 | 42 | 2.6 | - | - | - | 1138 | 3 |
| 6 | Yangtze River | 589 | Meidian | 114.31 | 31.10 | 200 | 9.8 | - | - | - | 1142 | 11 |
| 6 | Yangtze River | 398 | Meijiang | 107.57 | 27.98 | 13 | 0.7 | - | - | - | 919 | 0.64 |
| 6 | Yangtze River | 671 | Mengqiaochuan | 111.70 | 32.50 | 125 | 10.9 | - | - | - | 1112 | 12 |
| 6 | Yangtze River | 488 | Mengquan | 111.38 | 29.42 | 68 | 5.4 | - | - | - | 1033 | 6 |
| 6 | Yangtze River | 584 | Mingshan | 115.07 | 31.03 | 169 | 7.7 | - | - | - | 1157 | 9 |
| 6 | Yangtze River | 574 | Mingxing | 107.62 | 30.88 | 20 | 1.4 | - | - | - | 986 | 1.38 |
| 6 | Yangtze River | 52 | Mingyangguan | 117.74 | 28.58 | 42 | 2.6 | Main | 4 | 13 | 1167 | 3 |
| 6 | Yangtze River | 515 | Motanhe | 105.49 | 29.84 | 33 | 2.3 | - | - | - | 953 | 2 |
| 6 | Yangtze River | 641 | Mudong | 119.31 | 31.89 | 12 | 1.2 | - | - | - | 1112 | 1.33 |
| 6 | Yangtze River | 371 | Muguashan | 110.80 | 27.37 | 52 | 2.9 | - | - | - | 1001 | 3 |
| 6 | Yangtze River | 462 | Muqiaogou | 104.73 | 29.10 | 30 | 2.2 | - | - | - | 979 | 2 |
| 6 | Yangtze River | 437 | Muxi | 116.80 | 28.66 | 100 | 8.0 | - | - | - | 1195 | 10 |
| 6 | Yangtze River | 509 | Nanchuan | 114.41 | 29.72 | 110 | 4.0 | - | - | - | 1134 | 5 |
| 6 | Yangtze River | 75 | Nanhe 1 | 111.48 | 32.16 | 70 | 4.5 | Main | 23 | 107 | 1052 | 5 |
| 6 | Yangtze River | 327 | Nanhe 2 | 114.50 | 25.80 | 100 | 7.3 | - | - | - | 1154 | 8 |
| 6 | Yangtze River | 612 | Nanhe 3 | 112.03 | 31.39 | 35 | 1.0 | - | - | - | 1074 | 1.07 |
| 6 | Yangtze River | 678 | Nanshahe | 107.23 | 33.03 | 43 | 1.5 | - | - | - | 914 | 1.37 |
| 6 | Yangtze River | 564 | Niuchehe | 115.05 | 30.73 | 102 | 6.3 | - | - | - | 1164 | 7 |
| 6 | Yangtze River | 358 | Niuxingshan | 112.22 | 26.92 | 34 | 3.8 | - | - | - | 1060 | 4 |
| 6 | Yangtze River | 332 | Ouyanghai | 112.69 | 26.11 | 424 | 28.1 | - | - | - | 1089 | 31 |
| 6 | Yangtze River | 538 | Panlonghe | 105.20 | 30.28 | 40 | 1.8 | - | - | - | 927 | 2 |
| 6 | Yangtze River | 397 | Panqiao | 115.98 | 27.94 | 120 | 11.2 | - | - | - | 1167 | 13 |
| 6 | Yangtze River | 561 | Paoma | 111.74 | 30.70 | 42 | 1.7 | - | - | - | 1053 | 2 |
| 6 | Yangtze River | 540 | Paomatan | 105.34 | 30.32 | 32 | 4.8 | - | - | - | 929 | 4 |
| 6 | Yangtze River | 104 | Pengshui | 108.14 | 29.31 | 1465 | 45.7 | Main | 1750^b^ | 4867^b^ | 942 | 43 |
| 6 | Yangtze River | 103 | Pubugou | 102.83 | 29.23 | 5390 | 220.6 | Main | 3930^b^ | 10179^b^ | 952 | 210 |
| 6 | Yangtze River | 34 | Puding | 105.80 | 26.38 | 400 | 8.2 | Main | 75 | 340 | 1018 | 8 |
| 6 | Yangtze River | 452 | Qianjiapai | 118.31 | 28.87 | 21 | 0.5 | - | - | - | 1141 | 0.57 |
| 6 | Yangtze River | 386 | Qianjin 1 | 116.55 | 27.81 | 11 | 2.0 | - | - | - | 1157 | 2 |
| 6 | Yangtze River | 657 | Qianjin 2 | 111.66 | 32.10 | 17 | 0.3 | - | - | - | 1066 | 0.32 |
| 6 | Yangtze River | 835 | Qiaoqi | 102.81 | 30.39 | 212 | 4.0 | - | 240^b^ | 851^b^ | 954 | 4 |
| 6 | Yangtze River | 862 | Qingju | 106.11 | 30.70 | 117 | 4.0 | - | 136^b^ | 472^b^ | 947 | 4 |
| 6 | Yangtze River | 169 | Qingshan 1 | 114.02 | 29.43 | 429 | 9.4 | Sec | 7 | 79 | 1196.1^a^ | 11 |
| 6 | Yangtze River | 536 | Qingshan 2 | 119.80 | 30.25 | 220 | 12.3 | - | - | - | 1075 | 13 |
| 6 | Yangtze River | 339 | Qingshanlong | 113.41 | 26.29 | 123 | 5.4 | - | - | - | 1096 | 6 |
| 6 | Yangtze River | 356 | Qingtong | 116.28 | 26.87 | 24 | 2.1 | - | - | - | 1159 | 2 |
| 6 | Yangtze River | 445 | Qinjian | 117.19 | 28.81 | 20 | 0.3 | - | - | - | 1196 | 0.36 |
| 6 | Yangtze River | 632 | Qiushan | 119.11 | 31.79 | 13 | 1.3 | - | - | - | 1117 | 1.45 |
| 6 | Yangtze River | 446 | Qiyi 1 | 118.26 | 28.82 | 249 | 4.5 | - | - | - | 1149 | 5 |
| 6 | Yangtze River | 562 | Qiyi 2 | 106.62 | 30.71 | 25 | 0.7 | - | - | - | 952 | 0.67 |
| 6 | Yangtze River | 555 | Quanmin | 106.56 | 30.56 | 89 | 12.0 | - | - | - | 951 | 11 |
| 6 | Yangtze River | 480 | Qunying | 111.75 | 29.35 | 10 | 3.0 | - | - | - | 1042 | 3 |
| 6 | Yangtze River | 331 | Ridong | 116.21 | 26.03 | 100 | 7.6 | - | - | - | 1202 | 9 |
| 6 | Yangtze River | 106 | Sanbanxi | 109.05 | 26.61 | 4094 | 165.5 | Main | 1000^b^ | 1301^b^ | 989 | 164 |
| 6 | Yangtze River | 539 | Sancha | 104.27 | 30.32 | 229 | 35.5 | - | - | - | 917 | 33 |
| 6 | Yangtze River | 630 | Sandaohe | 111.81 | 31.76 | 161 | 3.8 | - | - | - | 1059 | 4 |
| 6 | Yangtze River | 608 | Sanhekou | 115.25 | 31.34 | 165 | 8.4 | - | - | - | 1142 | 10 |
| 6 | Yangtze River | 521 | Sanhulianjiang | 113.89 | 29.92 | 105 | 11.0 | - | - | - | 1125 | 12 |
| 6 | Yangtze River | 64 | Sanjiangkou | 111.50 | 29.61 | 220 | 24.1 | Main | 99^b^ | 125^b^ | 1037 | 25 |
| 6 | Yangtze River | 459 | Sanlixi | 111.28 | 29.00 | 10 | 1.4 | - | - | - | 1023 | 1.43 |
| 6 | Yangtze River | 468 | Sanxikou | 105.66 | 29.16 | 25 | 7.5 | - | - | - | 974 | 7 |
| 6 | Yangtze River | 843 | Seergu | 102.99 | 32.06 | 4 | 0.2 | - | 150^b^ | 610^b^ | 1014 | 0.20 |
| 6 | Yangtze River | 606 | Shahe | 119.44 | 31.32 | 10 | 9.1 | - | - | - | 1098 | 10 |
| 6 | Yangtze River | 415 | Shangtan | 117.98 | 28.33 | 20 | 2.0 | - | - | - | 1179 | 2 |
| 6 | Yangtze River | 381 | Shangyou 1 | 116.36 | 27.72 | 40 | 5.2 | - | - | - | 1158 | 6 |
| 6 | Yangtze River | 428 | Shangyou 2 | 115.09 | 28.52 | 200 | 9.9 | - | - | - | 1137 | 11 |
| 6 | Yangtze River | 513 | Shangyou 3 | 105.63 | 29.79 | 27 | 2.8 | - | - | - | 955 | 3 |
| 6 | Yangtze River | 617 | Shangyou 4 | 104.50 | 31.48 | 12 | 1.1 | - | - | - | 948 | 1.04 |
| 6 | Yangtze River | 25 | Shangyoujiang | 114.40 | 25.84 | 822 | 19.4 | Main | 72^b^ | 231^b^ | 1503.2^a^ | 29 |
| 6 | Yangtze River | 517 | Shanmen | 111.51 | 29.89 | 10 | 3.6 | - | - | - | 1037 | 4 |
| 6 | Yangtze River | 473 | Shantian | 117.07 | 29.26 | 22 | 5.9 | - | - | - | 1190 | 7 |
| 6 | Yangtze River | 119 | Shawan | 103.61 | 29.33 | 49 | 1.6 | Main | 480^b^ | 1811^b^ | 938 | 2 |
| 6 | Yangtze River | 622 | Shengzhong | 105.74 | 31.53 | 1339 | 40.8 | - | - | - | 951 | 39 |
| 6 | Yangtze River | 403 | Shenzihu | 110.75 | 28.17 | 100 | 4.1 | - | - | - | 991 | 4 |
| 6 | Yangtze River | 373 | Sheshang | 114.27 | 27.38 | 214 | 14.3 | - | - | - | 1092 | 16 |
| 6 | Yangtze River | 22 | Shibikeng | 115.81 | 25.57 | 58 | 5.7 | Main | 0 | 9 | 1218 | 7 |
| 6 | Yangtze River | 578 | Shilong | 112.88 | 30.95 | 100 | 4.2 | - | - | - | 1083 | 5 |
| 6 | Yangtze River | 391 | Shilu | 116.15 | 27.87 | 12 | 2.8 | - | - | - | 1163 | 3 |
| 6 | Yangtze River | 592 | Shimen 1 | 112.75 | 31.14 | 152 | 6.9 | - | - | - | 1083 | 7 |
| 6 | Yangtze River | 682 | Shimen 2 | 106.96 | 33.21 | 110 | 4.2 | - | - | - | 918 | 4 |
| 6 | Yangtze River | 645 | Shimenji | 111.78 | 31.91 | 162 | 8.1 | - | - | - | 1061 | 9 |
| 6 | Yangtze River | 550 | Shipan | 104.42 | 30.52 | 74 | 7.0 | - | - | - | 919 | 6 |
| 6 | Yangtze River | 81 | Shiquan | 108.23 | 33.04 | 412 | 21.9 | Main | 225^b^ | 782^b^ | 943 | 21 |
| 6 | Yangtze River | 663 | Shiti | 113.01 | 32.25 | 58 | 2.7 | - | - | - | 1124 | 3 |
| 6 | Yangtze River | 130 | Shiziping | 103.13 | 31.56 | 133 | 4.6 | Main | 130^b^ | 136^b^ | 989 | 5 |
| 6 | Yangtze River | 67 | Shizitan | 107.25 | 29.91 | 1027 | 29.2 | Main | 48 | 206 | 966 | 28 |
| 6 | Yangtze River | 531 | Shuanghe | 106.42 | 30.17 | 46 | 2.8 | - | 120^b^ | 375^b^ | 954 | 3 |
| 6 | Yangtze River | 330 | Shuanghua | 102.46 | 26.02 | 45 | 2.8 | - | - | - | 1198 | 3 |
| 6 | Yangtze River | 28 | Shuangpai | 111.68 | 25.95 | 690 | 40.3 | Main | 135 | 585 | 1066 | 43 |
| 6 | Yangtze River | 451 | Shuangxi | 117.70 | 28.85 | 69 | 4.3 | - | - | - | 1159 | 5 |
| 6 | Yangtze River | 537 | Shufangba | 105.28 | 30.26 | 70 | 7.1 | - | - | - | 930 | 7 |
| 6 | Yangtze River | 123 | Shuhe | 109.70 | 32.93 | 176 | 5.7 | Main | 270^b^ | 331^b^ | 1002 | 6 |
| 6 | Yangtze River | 411 | Shuiduili | 117.35 | 28.31 | 15 | 2.0 | - | - | - | 1191 | 2 |
| 6 | Yangtze River | 46 | Shuifumiao | 112.23 | 27.68 | 560 | 21.0 | Main | 30 | 109 | 1042 | 22 |
| 6 | Yangtze River | 871 | Shuijinguan | 103.09 | 29.97 | 6 | 0.2 | - | 63^b^ | 268^b^ | 938 | 0.19 |
| 6 | Yangtze River | 448 | Shuilanguang | 115.14 | 28.83 | 67 | 0.6 | - | - | - | 1129 | 0.68 |
| 6 | Yangtze River | 132 | Shuiniujia | 104.53 | 32.43 | 144 | 3.1 | Main | 70^b^ | 210^b^ | 961 | 3 |
| 6 | Yangtze River | 577 | Sian | 119.61 | 30.91 | 60 | 3.1 | - | - | - | 1086 | 3 |
| 6 | Yangtze River | 618 | Side | 106.71 | 31.49 | 42 | 2.1 | - | - | - | 945 | 2 |
| 6 | Yangtze River | 101 | Silin | 108.19 | 27.80 | 1654 | 38.4 | Main | 1050^b^ | 2506^b^ | 944 | 36 |
| 6 | Yangtze River | 301 | Songhuaba | 102.78 | 25.14 | 220 | 8.2 | - | - | - | 1172 | 10 |
| 6 | Yangtze River | 675 | Songjiachang | 113.49 | 32.76 | 132 | 14.1 | - | - | - | 1143 | 16 |
| 6 | Yangtze River | 507 | Songlin | 105.11 | 29.71 | 15 | 2.0 | - | - | - | 957 | 2 |
| 6 | Yangtze River | 108 | Suofengying | 106.37 | 26.97 | 201 | 4.2 | Main | 600^b^ | 1302^b^ | 950 | 4 |
| 6 | Yangtze River | 542 | Taihugang | 112.02 | 30.42 | 122 | 1.9 | - | - | - | 1051 | 2 |
| 6 | Yangtze River | 828 | Taipingyi | 103.48 | 31.10 | 1 | 0.0 | - | 260^b^ | 1606^b^ | 971 | 0.02 |
| 6 | Yangtze River | 652 | Taishan | 119.65 | 32.05 | 5 | 0.7 | - | - | - | 1111 | 0.78 |
| 6 | Yangtze River | 572 | Tangduhe | 111.34 | 30.84 | 34 | 0.7 | - | - | - | 1040 | 0.73 |
| 6 | Yangtze River | 362 | Tanhu | 116.67 | 27.13 | 100 | 2.3 | - | - | - | 1164 | 3 |
| 6 | Yangtze River | 668 | Tanjiahe | 110.04 | 32.40 | 17 | 0.2 | - | - | - | 1017 | 0.20 |
| 6 | Yangtze River | 619 | Tanshugang | 114.76 | 31.50 | 72 | 2.3 | - | - | - | 1153 | 3 |
| 6 | Yangtze River | 681 | Taohe | 110.90 | 33.14 | 72 | 1.0 | - | - | - | 1061 | 1.06 |
| 6 | Yangtze River | 412 | Taohuajiang | 112.10 | 28.32 | 100 | 2.2 | - | - | - | 1022 | 2 |
| 6 | Yangtze River | 388 | Taolin | 112.22 | 27.83 | 15 | 0.8 | - | - | - | 1041 | 0.83 |
| 6 | Yangtze River | 72 | Three Gorges Dam | 111.00 | 30.83 | 39300 | 852.9 | Main | 18300^b^ | 84370^b^ | 977.8^a^ | 834 |
| 6 | Yangtze River | 599 | Tianfumiao | 111.36 | 31.22 | 64 | 2.7 | - | - | - | 1025 | 3 |
| 6 | Yangtze River | 659 | Tianhekou | 113.47 | 32.14 | 117 | 7.4 | - | - | - | 1113 | 8 |
| 6 | Yangtze River | 825 | Tianhuangping pump storage | 119.62 | 30.53 | 18 | 0.6 | - | 1800^b^ | 1752^b^ | 1075 | 0.65 |
| 6 | Yangtze River | 588 | Tiantang | 115.62 | 31.09 | 140 | 6.1 | - | - | - | 1105 | 7 |
| 6 | Yangtze River | 570 | Tianzigang | 119.60 | 30.82 | 20 | 2.3 | - | - | - | 1085 | 2 |
| 6 | Yangtze River | 60 | Tieshan | 113.51 | 29.17 | 635 | 51.2 | Main | 4 | 16 | 1087 | 56 |
| 6 | Yangtze River | 61 | Tongjiezi | 103.63 | 29.26 | 210 | 7.7 | Main | 600 | 3210 | 932 | 7 |
| 6 | Yangtze River | 135 | Tongtou | 102.93 | 30.11 | 23 | 0.7 | Main | 80^b^ | 468^b^ | 946 | 0.66 |
| 6 | Yangtze River | 157 | Tuanjie | 116.08 | 26.89 | 200 | 14.8 | Sec | 1 | 3 | 1157 | 17 |
| 6 | Yangtze River | 591 | Tuanjie | 104.80 | 31.13 | 22 | 1.2 | - | - | - | 937 | 1.12 |
| 6 | Yangtze River | 478 | Tuxi | 106.97 | 29.33 | 18 | 3.3 | - | - | - | 948 | 3 |
| 6 | Yangtze River | 35 | Wanan | 114.80 | 26.45 | 2370 | 67.6 | Main | 533^b^ | 1596^b^ | 1172 | 79 |
| 6 | Yangtze River | 512 | Wangjiachang | 111.52 | 29.77 | 331 | 8.9 | - | 4 | 0 | 1074.6^a^ | 10 |
| 6 | Yangtze River | 514 | Wangying | 114.90 | 29.79 | 600 | 25.7 | - | - | - | 1137 | 29 |
| 6 | Yangtze River | 441 | Wangzhai | 118.10 | 28.69 | 35 | 2.1 | - | - | - | 1160 | 2 |
| 6 | Yangtze River | 560 | Wanjiagou | 106.25 | 30.70 | 10 | 0.3 | - | - | - | 949 | 0 |
| 6 | Yangtze River | 838 | Wanmipo | 109.51 | 28.80 | 328 | 11.9 | - | 240^b^ | 792^b^ | 981 | 12 |
| 6 | Yangtze River | 848 | Wawushan | 103.04 | 29.67 | 584 | 21.7 | - | 260^b^ | 699^b^ | 930 | 20 |
| 6 | Yangtze River | 595 | Weidoushan | 114.74 | 31.17 | 111 | 5.1 | - | - | - | 1162 | 6 |
| 6 | Yangtze River | 523 | Weishui | 111.57 | 29.96 | 576 | 24.8 | - | - | - | 1036 | 26 |
| 6 | Yangtze River | 348 | Weixi | 110.59 | 26.67 | 33 | 2.2 | - | - | - | 1026 | 2 |
| 6 | Yangtze River | 526 | Wenjiahe | 111.52 | 30.04 | 13 | 0.6 | - | - | - | 1033 | 0.62 |
| 6 | Yangtze River | 613 | Wenxiakou | 112.76 | 31.39 | 549 | 34.1 | - | - | - | 1094 | 37 |
| 6 | Yangtze River | 402 | Wuchuan | 113.38 | 28.13 | 16 | 0.3 | - | - | - | 1088 | 0.33 |
| 6 | Yangtze River | 407 | Wuhu | 116.96 | 28.22 | 22 | 6.6 | - | - | - | 1182 | 8 |
| 6 | Yangtze River | 44 | Wujiangdu | 106.76 | 27.32 | 2300 | 32.3 | Main | 1250^b^ | 2566^b^ | 959 | 31 |
| 6 | Yangtze River | 176 | Wumu | 107.27 | 30.73 | 53 | 9.5 | Sec | - | 386 | 975 | 9 |
| 6 | Yangtze River | 545 | Wupaishui | 106.38 | 30.45 | 50 | 2.3 | - | - | - | 950 | 2 |
| 6 | Yangtze River | 56 | Wuqiangxi | 110.93 | 28.78 | 4200 | 113.8 | Main | 1200^b^ | 5536^b^ | 1007 | 115 |
| 6 | Yangtze River | 660 | Wushan | 113.10 | 32.15 | 189 | 8.5 | - | - | - | 1117 | 9 |
| 6 | Yangtze River | 440 | Wuxi | 111.53 | 28.68 | 30 | 2.3 | - | - | - | 1019 | 2 |
| 6 | Yangtze River | 845 | Wuyiqiao | 101.54 | 28.93 | 1 | 0.0 | - | 120^b^ | 565^b^ | 1048 | 0.02 |
| 6 | Yangtze River | 586 | Xiajiasi | 114.46 | 31.06 | 300 | 18.2 | - | - | - | 1151 | 21 |
| 6 | Yangtze River | 424 | Xiakou | 118.54 | 28.47 | 64 | 4.2 | - | - | - | 1137 | 5 |
| 6 | Yangtze River | 605 | Xianghe | 112.10 | 31.31 | 15 | 0.3 | - | - | - | 1076 | 0.32 |
| 6 | Yangtze River | 556 | Xiangshuitan | 106.43 | 30.58 | 19 | 1.4 | - | - | - | 951 | 1.33 |
| 6 | Yangtze River | 634 | Xianjuemiao | 113.53 | 31.80 | 275 | 8.0 | - | - | - | 1130 | 9 |
| 6 | Yangtze River | 836 | Xiaoguanzi | 102.80 | 30.35 | 1 | 0.1 | - | 160^b^ | 836^b^ | 952 | 0.10 |
| 6 | Yangtze River | 503 | Xiaonanhai | 108.75 | 29.65 | 70 | 3.2 | - | - | - | 938 | 3 |
| 6 | Yangtze River | 616 | Xiaonanhe | 111.98 | 31.46 | 32 | 0.5 | - | - | - | 1072 | 0.54 |
| 6 | Yangtze River | 829 | Xiaotiandu | 102.17 | 30.08 | 1 | 0.0 | - | 240^b^ | 1076^b^ | 963 | 0.02 |
| 6 | Yangtze River | 377 | Xiayuan | 111.27 | 27.51 | 21 | 0.4 | - | - | - | 1011 | 0.40 |
| 6 | Yangtze River | 585 | Xibeikou | 111.35 | 31.05 | 210 | 6.6 | - | - | - | 1029 | 7 |
| 6 | Yangtze River | 321 | Xincun | 102.17 | 25.50 | 23 | 0.9 | - | - | - | 1199 | 1.08 |
| 6 | Yangtze River | 484 | Xindian | 104.07 | 29.39 | 26 | 2.0 | - | - | - | 961 | 2 |
| 6 | Yangtze River | 410 | Xingfu 1 | 116.60 | 28.28 | 100 | 7.3 | - | - | - | 1178 | 9 |
| 6 | Yangtze River | 597 | Xingfu 2 | 106.56 | 31.20 | 38 | 4.2 | - | - | - | 946 | 4 |
| 6 | Yangtze River | 66 | Xinqiao | 106.71 | 29.80 | 14 | 0.9 | Main | 0 | 3 | 970 | 1 |
| 6 | Yangtze River | 646 | Xionghe | 112.65 | 31.93 | 254 | 20.1 | - | - | - | 1125 | 23 |
| 6 | Yangtze River | 666 | Xipaizi | 111.94 | 32.37 | 282 | 31.7 | - | - | - | 1115 | 35 |
| 6 | Yangtze River | 129 | Xuecheng | 103.15 | 31.42 | 5390 | 90.5 | Main | 138^b^ | 585^b^ | 984 | 89 |
| 6 | Yangtze River | 620 | Xujiahe | 113.58 | 31.52 | 778 | 40.1 | - | - | - | 1129 | 45 |
| 6 | Yangtze River | 179 | Yahekou | 112.62 | 33.30 | 1316 | 42.1 | Sec | 9 | 20 | 1136 | 48 |
| 6 | Yangtze River | 337 | Yangquan | 112.27 | 26.27 | 43 | 3.1 | - | - | - | 1028 | 3 |
| 6 | Yangtze River | 579 | Yangshuhe | 111.82 | 30.96 | 10 | 0.8 | - | - | - | 1057 | 0.85 |
| 6 | Yangtze River | 413 | Yangtang | 116.50 | 28.33 | 18 | 0.5 | - | - | - | 1178 | 0.59 |
| 6 | Yangtze River | 558 | Yanjingkou | 107.53 | 30.62 | 20 | 0.6 | - | - | - | 987 | 0.59 |
| 6 | Yangtze River | 420 | Yanwutan | 110.48 | 28.43 | 103 | 5.1 | - | - | - | 1004 | 5 |
| 6 | Yangtze River | 853 | Yaoheba | 102.38 | 29.21 | 2 | 0.1 | - | 132^b^ | 625^b^ | 985 | 0.10 |
| 6 | Yangtze River | 854 | Yele | 102.38 | 29.20 | 298 | 5.8 | - | 240^b^ | 568^b^ | 985 | 6 |
| 6 | Yangtze River | 116 | Yilihe | 103.26 | 26.35 | 553 | 17.7 | Main | 321.5^b^ | 877^b^ | 1136 | 20 |
| 6 | Yangtze River | 629 | Yinghe | 112.60 | 31.74 | 119 | 11.7 | - | - | - | 1115 | 13 |
| 6 | Yangtze River | 422 | Yingshiling | 117.04 | 28.43 | 100 | 9.0 | - | - | - | 1193 | 11 |
| 6 | Yangtze River | 850 | Yinping | 104.53 | 32.43 | 1 | 0.0 | - | 100^b^ | 416^b^ | 961 | 0.02 |
| 6 | Yangtze River | 367 | Yinwanqiao | 114.84 | 27.24 | 30 | 2.1 | - | - | - | 1163 | 2 |
| 6 | Yangtze River | 118 | Yinzidu | 106.11 | 26.78 | 531 | 19.6 | Main | 360^b^ | 720^b^ | 965 | 19 |
| 6 | Yangtze River | 826 | Yixing pump storage | 119.75 | 31.31 | 5 | 0.1 | - | 1000^b^ | 1093^b^ | 1086 | 0.11 |
| 6 | Yangtze River | 317 | Youluokou | 114.30 | 25.38 | 116 | 9.2 | - | - | - | 1167 | 11 |
| 6 | Yangtze River | 553 | Yuanxing | 104.99 | 30.54 | 12 | 1.0 | - | - | - | 919 | 0.92 |
| 6 | Yangtze River | 872 | Yucheng | 103.08 | 29.98 | 11 | 0.6 | - | 60^b^ | 252^b^ | 938 | 0.56 |
| 6 | Yangtze River | 159 | Yudong | 103.55 | 27.40 | 360 | 7.1 | Sec | 150 | 900 | 1043 | 7 |
| 6 | Yangtze River | 382 | Yuejin | 116.51 | 27.73 | 23 | 2.5 | - | - | - | 1157 | 3 |
| 6 | Yangtze River | 669 | Yuetang | 119.09 | 32.41 | 20 | 2.5 | - | - | - | 1130 | 3 |
| 6 | Yangtze River | 624 | Yuntaishan | 111.89 | 31.57 | 134 | 7.1 | - | - | - | 1063 | 8 |
| 6 | Yangtze River | 137 | Yutang | 107.68 | 28.72 | 122 | 3.7 | Main | 75^b^ | 228^b^ | 949 | 4 |
| 6 | Yangtze River | 658 | Yutang | 106.66 | 32.10 | 21 | 1.0 | - | - | - | 923 | 0.92 |
| 6 | Yangtze River | 481 | Yutian | 117.37 | 29.35 | 20 | 1.0 | - | - | - | 1162 | 1.16 |
| 6 | Yangtze River | 26 | Zhanggang | 114.93 | 25.87 | 360 | 13.2 | Main | 0 | 1 | 1203 | 16 |
| 6 | Yangtze River | 583 | Zhanghe | 111.98 | 31.02 | 2030 | 55.7 | - | - | - | 1067 | 59 |
| 6 | Yangtze River | 544 | Zhangjiayan | 104.31 | 30.44 | 14 | 0.9 | - | - | - | 919 | 0.83 |
| 6 | Yangtze River | 360 | Zhangkeng | 114.63 | 27.10 | 10 | 0.2 | - | - | - | 1138 | 0.23 |
| 6 | Yangtze River | 434 | Zhangshuling | 115.20 | 28.61 | 21 | 0.8 | - | - | - | 1138 | 0.91 |
| 6 | Yangtze River | 680 | Zhaowan | 112.15 | 33.12 | 97 | 3.7 | - | - | - | 1129 | 4 |
| 6 | Yangtze River | 527 | Zhaoyang | 105.18 | 30.08 | 27 | 3.3 | - | - | - | 937 | 3 |
| 6 | Yangtze River | 167 | Zhelin | 115.50 | 29.21 | 7920 | 201.6 | Sec | 180 | 630 | 1143 | 230 |
| 6 | Yangtze River | 609 | Zhengjiahe | 113.29 | 31.34 | 193 | 9.0 | - | - | - | 1098 | 10 |
| 6 | Yangtze River | 635 | Zhenzhuya | 105.11 | 31.81 | 22 | 0.8 | - | - | - | 952 | 0.76 |
| 6 | Yangtze River | 51 | Zhexi | 111.13 | 28.33 | 3656 | 48.8 | Main | 447 | 2174 | 993 | 48 |
| 6 | Yangtze River | 409 | Zhongling 1 | 108.95 | 28.28 | 32 | 0.5 | - | - | - | 963 | 0.48 |
| 6 | Yangtze River | 418 | Zhongling 2 | 116.53 | 28.42 | 20 | 2.1 | - | - | - | 1183 | 2 |
| 6 | Yangtze River | 626 | Zhongshan | 119.06 | 31.64 | 10 | 4.0 | - | - | - | 1116 | 4 |
| 6 | Yangtze River | 651 | Zhongxing | 117.43 | 32.02 | 100 | 10.1 | - | - | - | 1163 | 12 |
| 6 | Yangtze River | 344 | Zhukeng | 115.97 | 26.50 | 24 | 2.2 | - | - | - | 1180 | 3 |
| 6 | Yangtze River | 49 | Zhushuqiao | 113.85 | 28.18 | 122 | 8.8 | Main | 24 | 73 | 1074 | 9 |
| 6 | Yangtze River | 665 | Zhuxihe | 109.65 | 32.34 | 23 | 0.2 | - | - | - | 995 | 0.20 |
| 6 | Yangtze River | 140 | Zhuxikou | 111.36 | 28.39 | 33 | 1.1 | Main | 74^b^ | 249^b^ | 1006 | 1.11 |
| 6 | Yangtze River | 443 | Zhuyuan | 111.14 | 28.78 | 144 | 2.4 | - | - | - | 1015 | 2 |
| 6 | Yangtze River | 131 | Zilanba | 105.69 | 32.40 | 35 | 1.5 | Main | 102^b^ | 272^b^ | 946 | 1.42 |
| 6 | Yangtze River | 851 | Ziyili | 104.53 | 32.42 | 1 | 0.0 | - | 130^b^ | 565^b^ | 961 | 0.01 |
| 6 | Yangtze RIVER | 161 | Ziyunshan | 115.81 | 27.83 | 116 | 12.1 | Sec | 1 | 1 | 1164 | 14 |
| 6 | Yangtze River | 522 | Zonggangshan | 103.23 | 29.95 | 32 | 3.4 | - | - | - | 933 | 3 |
| 7 | Southeast rivers | 456 | Andi | 119.62 | 28.96 | 70 | 1.3 | - | - | - | 1140 | 1.48 |
| 7 | Southeast rivers | 29 | Ansha | 117.11 | 26.03 | 740 | 34.3 | Main | 115^b^ | 640^b^ | 900^a^ | 31 |
| 7 | Southeast rivers | 463 | Baifeng | 120.04 | 29.13 | 26 | 0.8 | - | - | - | 1122 | 0.90 |
| 7 | Southeast rivers | 400 | Baishi | 120.85 | 28.11 | 14 | 0.4 | - | - | - | 1029 | 0.41 |
| 7 | Southeast rivers | 389 | Baizhanggji | 120.01 | 27.85 | 61 | 0.7 | - | - | - | 1091 | 0.76 |
| 7 | Southeast rivers | 165 | Changtan | 121.06 | 28.61 | 691 | 23.2 | Sec | 60^b^ | 170^b^ | 1129.1^a^ | 26 |
| 7 | Southeast rivers | 477 | Changyan | 119.96 | 29.31 | 12 | 0.6 | - | - | - | 1120 | 0.70 |
| 7 | Southeast rivers | 492 | Changzhao | 120.99 | 29.44 | 164 | 9.6 | - | - | - | 1066 | 10 |
| 7 | Southeast rivers | 501 | Chengcai | 120.39 | 29.59 | 116 | 10.0 | - | - | - | 1109 | 11 |
| 7 | Southeast rivers | 431 | Chengping 1 | 119.22 | 28.54 | 52 | 2.4 | - | - | - | 1124 | 3 |
| 7 | Southeast rivers | 433 | Chengping 2 | 119.25 | 28.56 | 9 | 0.9 | - | - | - | 1122 | 1.01 |
| 7 | Southeast rivers | 483 | Chengtou | 119.85 | 29.37 | 16 | 0.4 | - | - | - | 1116 | 0.45 |
| 7 | Southeast rivers | 40 | Chitan | 117.12 | 26.71 | 870 | 57.9 | Main | 100^b^ | 548^b^ | 817^a^ | 47 |
| 7 | Southeast rivers | 491 | Dongfanghong | 120.48 | 29.43 | 14 | 0.3 | - | - | - | 1110 | 0.33 |
| 7 | Southeast rivers | 311 | Dongxi 1 | 118.75 | 25.29 | 2 | 0.6 | - | - | - | 1248 | 0.75 |
| 7 | Southeast rivers | 385 | Dongxi 2 | 118.08 | 27.79 | 101 | 2.1 | - | - | - | 1137 | 2 |
| 7 | Southeast rivers | 24 | Dongzhang | 119.28 | 25.70 | 199 | 10.1 | Main | 1 | 1 | 1187 | 12 |
| 7 | Southeast rivers | 155 | Dongzhen | 118.98 | 25.48 | 440 | 12.5 | Sec | 6 | 29 | 1547.1^a^ | 19 |
| 7 | Southeast rivers | 519 | Fengle | 118.25 | 29.90 | 84 | 1.5 | - | - | - | 1063 | 2 |
| 7 | Southeast rivers | 172 | Fuchunjiang | 119.65 | 29.72 | 874 | 45.2 | Sec | 357.2^b^ | 1171^b^ | 1111 | 50 |
| 7 | Southeast rivers | 423 | Fuxi | 121.04 | 28.45 | 23 | 0.7 | - | - | - | 1006 | 0.70 |
| 7 | Southeast rivers | 464 | Gaochao | 119.47 | 29.13 | 12 | 2.2 | - | - | - | 1139 | 3 |
| 7 | Southeast rivers | 269 | Guguan | 121.02 | 24.21 | 18 | 0.3 | - | - | - | 1071 | 0.32 |
| 7 | Southeast rivers | 345 | Gutian | 118.80 | 26.57 | 641 | 10.8 | - | 186^b^ | 915^b^ | 950.8^a^ | 10 |
| 7 | Southeast rivers | 315 | Guyang | 118.68 | 25.31 | 20 | 0.9 | - | - | - | 1236 | 1.11 |
| 7 | Southeast rivers | 168 | Hengjin | 120.46 | 29.25 | 281 | 9.8 | Sec | 6 | 20 | 1319.7^a^ | 13 |
| 7 | Southeast rivers | 499 | Hengshan | 121.35 | 29.57 | 50 | 1.5 | - | - | - | 1050 | 2 |
| 7 | Southeast rivers | 506 | Hengxi | 121.59 | 29.71 | 40 | 1.6 | - | - | - | 1059 | 2 |
| 7 | Southeast rivers | 293 | Houqiao | 118.36 | 24.89 | 40 | 2.4 | - | - | - | 1271 | 3 |
| 7 | Southeast rivers | 866 | Huaan | 117.51 | 24.95 | 6 | 0.2 | - | 60^b^ | 424^b^ | 1195 | 0.24 |
| 7 | Southeast rivers | 447 | Huangtankou | 118.90 | 28.83 | 104 | 7.5 | - | - | - | 1144 | 9 |
| 7 | Southeast rivers | 416 | Human | 121.42 | 28.37 | 40 | 4.0 | - | - | - | 978 | 4 |
| 7 | Southeast rivers | 54 | Hunanzhen | 118.84 | 28.69 | 2060 | 44.0 | Main | 270 | 540 | 1136 | 50 |
| 7 | Southeast rivers | 474 | Huolushan | 119.33 | 29.29 | 20 | 2.4 | - | - | - | 1134 | 3 |
| 7 | Southeast rivers | 324 | Jianxin | 119.24 | 25.61 | 39 | 1.1 | - | - | - | 1187 | 1.31 |
| 7 | Southeast rivers | 516 | Jiaokou | 121.27 | 29.84 | 110 | 2.6 | - | - | - | 1070 | 3 |
| 7 | Southeast rivers | 865 | Jiemian | 118.05 | 25.92 | 1824 | 41.3 | - | 300^b^ | 410^b^ | 1172 | 48 |
| 7 | Southeast rivers | 419 | Jinkeng | 120.21 | 28.42 | 24 | 0.4 | - | - | - | 1088 | 0.44 |
| 7 | Southeast rivers | 496 | Jinkengling | 119.88 | 29.50 | 22 | 0.6 | - | - | - | 1107 | 0.66 |
| 7 | Southeast rivers | 458 | Jinlan | 119.48 | 28.99 | 95 | 6.0 | - | - | - | 1136 | 7 |
| 7 | Southeast rivers | 476 | Jinshantou | 119.34 | 29.30 | 22 | 3.5 | - | - | - | 1133 | 4 |
| 7 | Southeast rivers | 405 | Jinshuitan | 119.54 | 28.21 | 1393 | 42.0 | - | - | - | 1130 | 47 |
| 7 | Southeast rivers | 303 | Lingxi | 118.80 | 25.15 | 31 | 1.2 | - | - | - | 1254 | 2 |
| 7 | Southeast rivers | 166 | Lishimen | 120.77 | 29.07 | 200 | 9.2 | Sec | 6 | 25 | 1076 | 10 |
| 7 | Southeast rivers | 336 | Liutang | 117.92 | 26.21 | 44 | 3.6 | - | - | - | 1178 | 4 |
| 7 | Southeast rivers | 320 | Longmentan | 118.35 | 25.50 | 53 | 3.1 | - | - | - | 1195 | 4 |
| 7 | Southeast rivers | 278 | Mingteh | 120.89 | 24.58 | 18 | 0.8 | - | - | - | 1079 | 0.86 |
| 7 | Southeast rivers | 467 | Nanjiang | 120.43 | 29.14 | 117 | 7.5 | - | - | - | 1103 | 8 |
| 7 | Southeast rivers | 490 | Nanshan | 120.59 | 29.42 | 105 | 3.5 | - | - | - | 1108 | 4 |
| 7 | Southeast rivers | 370 | Nanxi | 120.13 | 27.36 | 67 | 0.6 | - | - | - | 1090 | 0.65 |
| 7 | Southeast rivers | 57 | Niutoushan | 121.27 | 28.84 | 300 | 13.0 | Main | 100^b^ | 280^b^ | 993 | 13 |
| 7 | Southeast rivers | 376 | Qiaodun | 120.30 | 27.48 | 66 | 1.1 | - | - | - | 1090 | 1.20 |
| 7 | Southeast rivers | 489 | Qiaoxi | 120.18 | 29.42 | 14 | 0.4 | - | - | - | 1114 | 0.45 |
| 7 | Southeast rivers | 486 | Qiaoying | 121.16 | 29.40 | 100 | 0.6 | - | - | - | 1047 | 0.63 |
| 7 | Southeast rivers | 449 | Qingxikou | 119.89 | 28.84 | 15 | 0.6 | - | - | - | 1138 | 0.68 |
| 7 | Southeast rivers | 328 | Qinyuan | 116.90 | 25.88 | 15 | 0.2 | - | - | - | 1164 | 0.23 |
| 7 | Southeast rivers | 485 | Qixi | 118.35 | 29.40 | 60 | 3.6 | - | - | - | 1085 | 4 |
| 7 | Southeast rivers | 460 | Sanduxi | 120.07 | 29.03 | 11 | 0.1 | - | - | - | 1119 | 0.11 |
| 7 | Southeast rivers | 305 | Shanmei | 118.41 | 25.17 | 656 | 12.7 | - | 60 | 130 | 1767.1^a^ | 22 |
| 7 | Southeast rivers | 30 | Shanzi | 119.61 | 26.24 | 180 | 5.3 | Main | 45 | 173 | 1154 | 6 |
| 7 | Southeast rivers | 39 | Shaxikou | 118.08 | 26.59 | 164 | 14.3 | Main | 300^b^ | 1182^b^ | 1176 | 17 |
| 7 | Southeast rivers | 287 | Shibi 1 | 118.36 | 24.77 | 62 | 2.1 | - | - | - | 1283 | 3 |
| 7 | Southeast rivers | 494 | Shibi 2 | 120.34 | 29.48 | 110 | 3.7 | - | - | - | 1111 | 4 |
| 7 | Southeast rivers | 292 | Shihmen | 121.25 | 24.81 | 309 | 5.5 | - | - | - | 1079 | 6 |
| 7 | Southeast rivers | 408 | Shitang | 119.66 | 28.23 | 83 | 5.2 | - | - | - | 1149 | 6 |
| 7 | Southeast rivers | 335 | Shuidong | 118.20 | 26.17 | 110 | 6.6 | - | 80^b^ | 320^b^ | 1168 | 8 |
| 7 | Southeast rivers | 31 | Shuikou | 118.81 | 26.31 | 2600 | 56.2 | Main | 1400^b^ | 6974^b^ | 1000^a^ | 56 |
| 7 | Southeast rivers | 164 | Taihu | 121.24 | 28.49 | 30 | 3.3 | Sec | - | 33 | 988 | 3 |
| 7 | Southeast rivers | 461 | Taiping | 120.18 | 29.03 | 52 | 3.7 | - | - | - | 1113 | 4 |
| 7 | Southeast rivers | 289 | Tingxi | 118.14 | 24.80 | 49 | 1.4 | - | - | - | 1494.9^a^ | 2 |
| 7 | Southeast rivers | 504 | Tingxia | 121.22 | 29.66 | 153 | 3.1 | - | - | - | 1061 | 3 |
| 7 | Southeast rivers | 839 | Tongbai pump storage | 121.00 | 29.20 | 12 | 0.4 | - | 1200^b^ | 1226^b^ | 1056 | 0.42 |
| 7 | Southeast rivers | 493 | Tongjiqiao | 119.84 | 29.45 | 81 | 4.3 | - | - | - | 1111 | 5 |
| 7 | Southeast rivers | 465 | Tongshanyuan | 118.94 | 29.13 | 171 | 8.1 | - | - | - | 1105 | 9 |
| 7 | Southeast rivers | 242 | Tsengwen | 120.54 | 23.25 | 708 | 11.9 | - | - | - | 1284 | 15 |
| 7 | Southeast rivers | 154 | Wananxi | 117.06 | 25.40 | 230 | 10.6 | Sec | 45 | 136 | 1000^a^ | 11 |
| 7 | Southeast rivers | 375 | Wujiayuan | 120.44 | 27.44 | 19 | 0.3 | - | - | - | 1086 | 0.33 |
| 7 | Southeast rivers | 237 | Wushantou | 120.37 | 23.20 | 148 | 5.6 | - | - | - | 1441 | 8 |
| 7 | Southeast rivers | 299 | Wutan | 118.57 | 25.08 | 123 | 4.0 | - | - | - | 1260 | 5 |
| 7 | Southeast rivers | 471 | Wuzhangyan | 120.71 | 29.21 | 25 | 0.6 | - | - | - | 1097 | 0.66 |
| 7 | Southeast rivers | 444 | Xikou | 121.41 | 28.79 | 20 | 2.4 | - | - | - | 993 | 2 |
| 7 | Southeast rivers | 63 | Xinan Jiang | 119.21 | 29.49 | 21626 | 424.3 | Main | 850^b^ | 2383^b^ | 1215.4^a^ | 516 |
| 7 | Southeast rivers | 455 | Xinfan | 119.33 | 28.95 | 40 | 2.6 | - | - | - | 1129 | 3 |
| 7 | Southeast rivers | 271 | Yangmei | 117.87 | 24.26 | 52 | 2.7 | - | - | - | 1261 | 3 |
| 7 | Southeast rivers | 453 | Yangxi | 120.15 | 28.89 | 55 | 5.4 | - | - | - | 1106 | 6 |
| 7 | Southeast rivers | 475 | Yankou | 119.92 | 29.29 | 50 | 3.5 | - | - | - | 1121 | 4 |
| 7 | Southeast rivers | 524 | Yanshiling | 119.63 | 30.01 | 50 | 3.6 | - | - | - | 1068 | 4 |
| 7 | Southeast rivers | 874 | Yongkou | 118.31 | 26.29 | 35 | 1.2 | - | 50^b^ | 221^b^ | 1168 | 1.40 |
| 7 | Southeast rivers | 450 | Yuankou | 119.63 | 28.84 | 24 | 0.6 | - | - | - | 1142 | 0.69 |
| 7 | Southeast rivers | 142 | Zhaokou | 118.02 | 26.69 | 61 | 2.0 | Main | 60^b^ | 249^b^ | 1177 | 2 |
| 7 | Southeast rivers | 326 | Zheya | 118.31 | 25.80 | 60 | 3.4 | - | - | - | 1155 | 4 |
| 7 | Southeast rivers | 482 | Zhiyan | 119.35 | 29.37 | 40 | 2.4 | - | - | - | 1124 | 3 |
| 7 | Southeast rivers | 401 | Zhongqian | 120.86 | 28.12 | 25 | 0.3 | - | - | - | 1030 | 0.31 |
| 7 | Southeast rivers | 858 | Zhouning | 119.38 | 27.07 | 47 | 1.6 | - | 250^b^ | 787^b^ | 1090 | 2 |
| 8 | Zhujiang | 261 | Baidonghe | 106.87 | 23.82 | 92 | 2.6 | - | - | - | 1250 | 3 |
| 8 | Zhujiang | 121 | Bailongtan | 108.13 | 23.86 | 340 | 12.4 | Main | 192^b^ | 832^b^ | 1176 | 15 |
| 8 | Zhujiang | 235 | Baipenzhu | 115.03 | 23.08 | 1220 | 29.3 | - | 24 | 86 | 1284 | 38 |
| 8 | Zhujiang | 240 | Baishi | 110.17 | 23.22 | 17 | 0.7 | - | 92 | 320 | 1211 | 0.85 |
| 8 | Zhujiang | 863 | Baishiyao | 113.43 | 24.14 | 108 | 3.7 | - | 92^b^ | 342^b^ | 1232 | 5 |
| 8 | Zhujiang | 210 | Banling | 109.52 | 18.35 | 14 | 0.7 | - | - | - | 1502 | 1.05 |
| 8 | Zhujiang | 211 | Baogu | 109.24 | 18.42 | 22 | 0.8 | - | - | - | 1509 | 1.21 |
| 8 | Zhujiang | 153 | Chaishitan | 103.28 | 25.01 | 440 | 4.0 | Sec | 60 | 183 | 1178 | 5 |
| 8 | Zhujiang | 313 | Changhe | 112.49 | 25.31 | 34 | 2.6 | - | - | - | 1086 | 3 |
| 8 | Zhujiang | 15 | Changhu | 113.47 | 24.13 | 150 | 8.2 | Main | 76^b^ | 340^b^ | 1235 | 10 |
| 8 | Zhujiang | 212 | Changmao | 109.08 | 18.63 | 171 | 7.4 | - | - | - | 1387 | 10 |
| 8 | Zhujiang | 285 | Changtan | 116.13 | 24.70 | 172 | 8.4 | - | - | - | 1262 | 11 |
| 8 | Zhujiang | 13 | Chengbihe | 106.64 | 23.95 | 1130 | 25.0 | Main | 26 | 114 | 1218 | 30 |
| 8 | Zhujiang | 215 | Daguanba | 108.98 | 19.02 | 1710 | 35.2 | - | - | - | 1407 | 50 |
| 8 | Zhujiang | 855 | Daguangba | 108.68 | 19.32 | 1700 | 66.1 | - | 240^b^ | 529^b^ | 1435 | 95 |
| 8 | Zhujiang | 258 | Dahongjiang | 107.54 | 23.75 | 66 | 2.6 | - | - | - | 1216 | 3 |
| 8 | Zhujiang | 257 | Dahua | 107.98 | 23.73 | 1210 | 31.2 | - | 556^b^ | 2420^b^ | 1191 | 37 |
| 8 | Zhujiang | 150 | Dakai | 109.71 | 23.41 | 391 | 14.9 | Sec | 60 | 255 | 1184 | 18 |
| 8 | Zhujiang | 146 | Dalongdong 1 | 112.64 | 22.03 | 265 | 14.1 | Sec | 2 | 4 | 1247 | 18 |
| 8 | Zhujiang | 250 | Dalongdong 2 | 108.58 | 23.59 | 151 | 14.1 | - | - | - | 1190 | 17 |
| 8 | Zhujiang | 148 | Dawangtan | 108.32 | 22.59 | 638 | 16.1 | Sec | 2 | 5 | 1224 | 20 |
| 8 | Zhujiang | 272 | Dongfeng | 102.58 | 24.37 | 89 | 2.9 | - | - | - | 1188 | 3 |
| 8 | Zhujiang | 225 | Donghu | 112.21 | 22.01 | 123 | 3.5 | - | - | - | 1006^a^ | 4 |
| 8 | Zhujiang | 107 | Dongjing | 105.72 | 25.60 | 955 | 17.2 | Main | 880^b^ | 2585^b^ | 1078 | 19 |
| 8 | Zhujiang | 20 | Dongluo | 113.49 | 25.22 | 31 | 2.2 | Main | 6 | 15 | 1131 | 2 |
| 8 | Zhujiang | 312 | Dumu | 104.10 | 25.30 | 100 | 14.5 | - | - | - | 1130 | 16 |
| 8 | Zhujiang | 263 | Dutang | 117.33 | 23.89 | 16 | 0.8 | - | - | - | 1285 | 1 |
| 8 | Zhujiang | 112 | Etan | 108.61 | 23.97 | 950 | 36.0 | Main | 600^b^ | 2653^b^ | 1167 | 42 |
| 8 | Zhujiang | 260 | Feilaixia 1 | 113.25 | 23.80 | 1904 | 80.2 | - | - | - | 1241 | 100 |
| 8 | Zhujiang | 857 | Feilaixia 2 | 113.27 | 23.80 | 1900 | 74.3 | - | 140^b^ | 602^b^ | 1252 | 93 |
| 8 | Zhujiang | 262 | Fenghuang | 116.66 | 23.89 | 60 | 3.5 | - | - | - | 1261 | 4 |
| 8 | Zhujiang | 274 | Fengshuba | 115.37 | 24.42 | 1940 | 92.8 | - | 176^b^ | 661^b^ | 1276 | 118 |
| 8 | Zhujiang | 228 | Fengtinghe | 108.24 | 22.31 | 616 | 11.9 | - | - | - | 1223 | 15 |
| 8 | Zhujiang | 267 | Fengtou | 117.28 | 24.06 | 175 | 2.8 | - | - | - | 1203 | 3 |
| 8 | Zhujiang | 265 | Fengxi | 116.69 | 23.95 | 31 | 0.5 | - | - | - | 1257 | 0.63 |
| 8 | Zhujiang | 4 | Fucao | 111.20 | 22.48 | 60 | 2.4 | Main | 10 | 44 | 1231 | 3 |
| 8 | Zhujiang | 219 | Fushan | 109.95 | 19.82 | 68 | 5.3 | - | - | - | 1432 | 8 |
| 8 | Zhujiang | 277 | Fushi | 115.80 | 24.56 | 10 | 0.6 | - | - | - | 1282 | 0.77 |
| 8 | Zhujiang | 147 | Gaozhou | 110.98 | 22.15 | 1151 | 21.4 | Sec | 31 | 101 | 1259 | 27 |
| 8 | Zhujiang | 111 | Guangxichangzhou | 111.21 | 23.42 | 5600 | 313.3 | Main | 630^b^ | 2690^b^ | 1225 | 384 |
| 8 | Zhujiang | 100 | Guangzhao | 105.25 | 25.63 | 3135 | 125.3 | Main | 1040^b^ | 1913^b^ | 1076 | 135 |
| 8 | Zhujiang | 9 | Guangzhou pump storage | 113.96 | 23.77 | 240 | 2.4 | Main | 2400^b^ | 3756^b^ | 1257 | 3 |
| 8 | Zhujiang | 297 | Guanyang | 116.78 | 25.04 | 22 | 0.7 | - | - | - | 1189 | 0.83 |
| 8 | Zhujiang | 152 | Guishi | 111.29 | 24.66 | 595 | 34.1 | Sec | 12 | 66 | 1104 | 38 |
| 8 | Zhujiang | 300 | Guodihe | 104.84 | 25.10 | 28 | 0.9 | - | - | - | 1088 | 0.98 |
| 8 | Zhujiang | 222 | Hedi | 110.30 | 21.72 | 1462 | 51.9 | - | - | - | 1407.4^a^ | 73 |
| 8 | Zhujiang | 151 | Hemianshi | 111.76 | 24.05 | 296 | 9.9 | Sec | 68 | 360 | 1141 | 11 |
| 8 | Zhujiang | 248 | Hengjiang | 115.80 | 23.49 | 69 | 1.9 | - | - | - | 1274 | 2 |
| 8 | Zhujiang | 224 | Hepu | 109.60 | 21.93 | 1082 | 18.9 | - | - | - | 1236 | 23 |
| 8 | Zhujiang | 270 | Heshui | 115.69 | 24.26 | 90 | 2.3 | - | - | - | 1315 | 3 |
| 8 | Zhujiang | 220 | Hexi | 110.17 | 20.50 | 17 | 1.1 | - | - | - | 1350 | 1.49 |
| 8 | Zhujiang | 145 | Hongchaojiang | 109.15 | 21.80 | 1000 | 25.2 | Sec | 1 | 3 | 1238 | 31 |
| 8 | Zhujiang | 109 | Hongjiadu | 109.85 | 26.04 | 4947 | 201.7 | Main | 600^b^ | 593^b^ | 1004 | 202 |
| 8 | Zhujiang | 296 | Huanggang | 116.92 | 25.01 | 32 | 1.0 | - | - | - | 1187 | 1.19 |
| 8 | Zhujiang | 8 | Huanglongdai | 113.74 | 23.77 | 95 | 7.5 | Main | 9 | 25 | 1246 | 9 |
| 8 | Zhujiang | 223 | Huangsha | 111.30 | 21.79 | 55 | 1.4 | - | - | - | 1268 | 2 |
| 8 | Zhujiang | 249 | Huangshandong | 114.55 | 23.57 | 31 | 3.5 | - | - | - | 1296 | 5 |
| 8 | Zhujiang | 286 | Huangtian | 115.88 | 24.71 | 10 | 1.4 | - | - | - | 1263 | 2 |
| 8 | Zhujiang | 11 | Huashan | 112.57 | 23.90 | 63 | 3.5 | Main | 0 | 1 | 1191 | 4 |
| 8 | Zhujiang | 824 | Huizhou pump storage | 114.32 | 23.16 | 57 | 2.3 | - | 2100^b^ | 1520^b^ | 1310 | 3 |
| 8 | Zhujiang | 218 | Jiatan | 110.04 | 19.65 | 46 | 1.5 | - | - | - | 1403 | 2 |
| 8 | Zhujiang | 3 | Jinjiang | 112.17 | 22.27 | 480 | 23.9 | Main | 20 | 48 | 1232 | 29 |
| 8 | Zhujiang | 139 | Jinjitan | 107.64 | 23.21 | 231 | 13.0 | Main | 72^b^ | 254^b^ | 1229 | 16 |
| 8 | Zhujiang | 252 | Jintian | 110.06 | 23.62 | 39 | 2.7 | - | - | - | 1173 | 3 |
| 8 | Zhujiang | 310 | Jitou | 116.56 | 25.27 | 32 | 2.2 | - | - | - | 1177 | 3 |
| 8 | Zhujiang | 247 | Jiuwantan | 113.40 | 23.47 | 39 | 1.4 | - | - | - | 1276 | 2 |
| 8 | Zhujiang | 229 | Kelan | 107.64 | 22.40 | 323 | 10.0 | - | - | - | 1246 | 12 |
| 8 | Zhujiang | 279 | Lalang | 108.27 | 24.59 | 112 | 9.7 | - | - | - | 1155 | 11 |
| 8 | Zhujiang | 243 | Lianhe | 113.91 | 23.28 | 82 | 2.4 | - | - | - | 1296 | 3 |
| 8 | Zhujiang | 230 | Lingdong | 109.39 | 22.47 | 179 | 4.7 | - | - | - | 1199 | 6 |
| 8 | Zhujiang | 149 | Liuchen | 110.34 | 23.20 | 333 | 10.0 | Sec | 5 | 9 | 1218 | 12 |
| 8 | Zhujiang | 7 | Liuxihe | 113.77 | 23.75 | 350 | 9.5 | Main | 42 | 154 | 1247 | 12 |
| 8 | Zhujiang | 251 | Longjing 1 | 116.07 | 23.60 | 29 | 2.3 | - | - | - | 1277 | 3 |
| 8 | Zhujiang | 254 | Longjing 2 | 116.07 | 23.65 | 166 | 7.4 | - | - | - | 1277 | 9 |
| 8 | Zhujiang | 105 | Longtan 1 | 107.05 | 25.03 | 16210 | 696.4 | Main | 4900^b^ | 11474^b^ | 1139 | 793 |
| 8 | Zhujiang | 236 | Longtan 2 | 115.90 | 23.09 | 131 | 2.8 | - | 14 | 52 | 1272 | 4 |
| 8 | Zhujiang | 18 | Lubuge | 104.54 | 24.82 | 110 | 4.1 | Main | 600^b^ | 2358^b^ | 1040^a^ | 4 |
| 8 | Zhujiang | 276 | Luodong | 108.85 | 24.53 | 132 | 8.5 | - | - | - | 1144 | 10 |
| 8 | Zhujiang | 21 | Mashi | 109.43 | 25.46 | 270 | 20.8 | Main | 100 | 455 | 1053 | 22 |
| 8 | Zhujiang | 273 | Meixi | 115.93 | 24.38 | 10 | 1.4 | - | - | - | 1299 | 2 |
| 8 | Zhujiang | 283 | Mianhuatan | 116.60 | 24.66 | 2210 | 92.6 | - | 600^b^ | 1934^b^ | 1240 | 115 |
| 8 | Zhujiang | 226 | Naban | 108.00 | 22.14 | 824 | 33.6 | - | - | - | 1226 | 41 |
| 8 | Zhujiang | 245 | Nangao | 115.58 | 23.35 | 79 | 0.9 | - | - | - | 1271 | 1.14 |
| 8 | Zhujiang | 288 | Nanshui | 113.21 | 24.79 | 1243 | 47.3 | - | 79^b^ | 258^b^ | 919.4^a^ | 43 |
| 8 | Zhujiang | 1 | Niululing | 110.20 | 19.01 | 780 | 25.3 | Main | 80 | 281 | 1412 | 36 |
| 8 | Zhujiang | 117 | Pingban | 105.49 | 24.82 | 278 | 10.0 | Main | 405^b^ | 1024^b^ | 1130 | 11 |
| 8 | Zhujiang | 238 | Pinglong | 109.41 | 23.22 | 125 | 9.0 | - | - | - | 1182 | 11 |
| 8 | Zhujiang | 255 | Qiji | 114.63 | 23.67 | 10 | 0.6 | - | - | - | 1293 | 0.78 |
| 8 | Zhujiang | 322 | Qingshitan | 110.23 | 25.53 | 560 | 23.7 | - | - | - | 1070 | 25 |
| 8 | Zhujiang | 856 | Qingxi | 116.70 | 24.37 | 80 | 3.4 | - | 144^b^ | 451^b^ | 1243 | 4 |
| 8 | Zhujiang | 17 | Quanshui | 113.09 | 24.72 | 222 | 2.2 | Main | 24 | 114 | 1129 | 2 |
| 8 | Zhujiang | 221 | Renshui | 111.17 | 21.58 | 1 | 0.7 | - | - | - | 1272 | 0.89 |
| 8 | Zhujiang | 268 | Sankeng | 112.03 | 24.11 | 47 | 1.7 | - | - | - | 1145 | 2 |
| 8 | Zhujiang | 291 | Shanlandian | 112.58 | 24.80 | 19 | 0.6 | - | - | - | 1113 | 0.67 |
| 8 | Zhujiang | 136 | Shanxiu | 108.03 | 22.77 | 606 | 22.5 | Main | 78^b^ | 318^b^ | 1236 | 28 |
| 8 | Zhujiang | 244 | Shepo | 110.23 | 23.33 | 60 | 4.0 | - | - | - | 1209 | 5 |
| 8 | Zhujiang | 233 | Shiliutan | 116.15 | 23.07 | 111 | 0.6 | - | - | - | 1286 | 0.77 |
| 8 | Zhujiang | 216 | Shilu | 109.09 | 19.25 | 141 | 8.6 | - | - | - | 1385 | 12 |
| 8 | Zhujiang | 10 | Shuangdao | 111.46 | 23.81 | 210 | 4.3 | Main | 13 | 55 | 1188 | 5 |
| 8 | Zhujiang | 253 | Shuidongpi | 114.41 | 23.63 | 61 | 2.3 | - | - | - | 1290 | 3 |
| 8 | Zhujiang | 232 | Songmushan | 113.88 | 22.89 | 100 | 4.1 | - | - | - | 1309 | 5 |
| 8 | Zhujiang | 217 | Songtao | 109.68 | 19.33 | 3345 | 58.0 | - | - | - | 1402 | 81 |
| 8 | Zhujiang | 239 | Tangkeng | 116.24 | 23.22 | 33 | 1.0 | - | - | - | 1286 | 1.29 |
| 8 | Zhujiang | 12 | Tangxi | 116.87 | 23.90 | 380 | 10.8 | Main | 8 | 23 | 1457^a^ | 16 |
| 8 | Zhujiang | 295 | Tanling | 112.65 | 24.97 | 176 | 10.9 | - | - | - | 1105 | 12 |
| 8 | Zhujiang | 19 | Tianshengqiao 1 | 105.10 | 24.94 | 10260 | 98.3 | Main | 1200 | 5200 | 1085.1^a^ | 107 |
| 8 | Zhujiang | 294 | Tianshengqiao 2 | 105.15 | 24.96 | 14 | 0.1 | - | 1320^b^ | 4872^b^ | 1106 | 0.11 |
| 8 | Zhujiang | 259 | Tiantangshan | 114.17 | 23.80 | 243 | 13.2 | - | - | - | 1266 | 17 |
| 8 | Zhujiang | 138 | Tuanpo | 106.67 | 25.72 | 1 | 0.0 | Main | 80^b^ | 230^b^ | 1054 | 0.02 |
| 8 | Zhujiang | 227 | Tunliu | 108.28 | 22.27 | 230 | 26.0 | - | 0 | 0 | 1219 | 32 |
| 8 | Zhujiang | 214 | Tuoxin | 108.79 | 18.85 | 99 | 2.9 | - | - | - | 1425 | 4 |
| 8 | Zhujiang | 275 | Wengong | 115.72 | 24.45 | 27 | 0.3 | - | - | - | 1297 | 0 |
| 8 | Zhujiang | 309 | Wudugang | 114.76 | 25.25 | 29 | 2.0 | - | - | - | 1227 | 2 |
| 8 | Zhujiang | 231 | Wusijiang | 109.66 | 22.74 | 104 | 5.6 | - | 6 | 18 | 1192 | 7 |
| 8 | Zhujiang | 234 | Wusongling | 116.26 | 23.07 | 30 | 0.9 | - | - | - | 1286 | 1.16 |
| 8 | Zhujiang | 241 | Xiangang | 114.12 | 23.25 | 100 | 8.0 | - | - | - | 1305 | 10 |
| 8 | Zhujiang | 306 | Xiangshuiba | 103.82 | 25.18 | 20 | 2.7 | - | - | - | 1157 | 3 |
| 8 | Zhujiang | 246 | Xianhu | 108.08 | 23.38 | 130 | 9.6 | - | - | - | 1216 | 12 |
| 8 | Zhujiang | 2 | Xiaojiang | 109.59 | 21.99 | 1000 | 29.5 | Main | 144 | 716 | 1218 | 36 |
| 8 | Zhujiang | 213 | Xiaomei | 109.95 | 18.68 | 49 | 2.7 | - | - | - | 1384 | 4 |
| 8 | Zhujiang | 318 | Xiaoxiang | 103.76 | 25.46 | 41 | 2.7 | - | - | - | 1164 | 3 |
| 8 | Zhujiang | 323 | Xihe | 103.72 | 25.60 | 100 | 3.2 | - | 1800^b^ | 661^b^ | 1164 | 4 |
| 8 | Zhujiang | 5 | Xijin | 109.24 | 22.65 | 3000 | 66.5 | Main | 242.2^b^ | 950^b^ | 1208 | 80 |
| 8 | Zhujiang | 6 | Xinfengjiang | 114.65 | 23.73 | 13896 | 264.3 | Main | 335^b^ | 713^b^ | 852.4^a^ | 225 |
| 8 | Zhujiang | 256 | Xinxihe | 116.29 | 23.67 | 70 | 1.6 | - | - | - | 1278 | 2 |
| 8 | Zhujiang | 319 | Xiyuan | 116.47 | 25.47 | 12 | 0.3 | - | - | - | 1183 | 0.36 |
| 8 | Zhujiang | 14 | Yantan | 107.51 | 24.05 | 2610 | 85.1 | Main | 1210^b^ | 6038^b^ | 1646^a^ | 140 |
| 8 | Zhujiang | 264 | Yitang | 115.54 | 23.94 | 160 | 13.3 | - | - | - | 1292 | 17 |
| 8 | Zhujiang | 302 | Yongqinghe | 103.60 | 25.15 | 20 | 2.9 | - | - | - | 1168 | 3 |
| 8 | Zhujiang | 316 | Zoumalong | 114.72 | 25.37 | 23 | 2.9 | - | - | - | 1222 | 4 |
| 8 | Zhujiang | 266 | Zumalin | 117.56 | 23.97 | 34 | 1.3 | - | - | - | 1261 | 2 |
| 9 | Southwest rivers | 308 | Beimiao | 99.21 | 25.25 | 74 | 3.6 | - | - | - | 1142 | 4 |
| 9 | Southwest rivers | 110 | Dachaoshan | 100.36 | 24.05 | 890 | 20.2 | Main | 1350^b^ | 6225^b^ | 1184.2^a^ | 24 |
| 9 | Southwest rivers | 125 | Dayingjiang | 97.91 | 24.68 | 0 | 0.0 | Main | 108^b^ | 475^b^ | 1118 | 0.004 |
| 9 | Southwest rivers | 115 | Gelantan | 102.06 | 22.71 | 409 | 9.1 | Main | 450^b^ | 1610^b^ | 1214 | 11 |
| 9 | Southwest rivers | 338 | Haixihai | 99.98 | 26.29 | 62 | 4.0 | - | - | - | 1111 | 4 |
| 9 | Southwest rivers | 99 | Jinghong | 100.78 | 22.05 | 1139 | 43.5 | Main | 1750^b^ | 6176^b^ | 1162.1^a^ | 51 |
| 9 | Southwest rivers | 827 | Jufudu | 101.92 | 22.85 | 169 | 4.2 | - | 285^b^ | 1033^b^ | 1208 | 5 |
| 9 | Southwest rivers | 832 | Longma | 102.02 | 23.54 | 599 | 22.2 | - | 285^b^ | 959^b^ | 1680.1^a^ | 37 |
| 9 | Southwest rivers | 869 | Lvshuihe | 103.37 | 23.37 | 0 | 0.0 | - | 65.5^b^ | 248^b^ | 1448.9^a^ | 0.01 |
| 9 | Southwest rivers | 124 | Malutang | 104.74 | 23.01 | 550 | 9.2 | Main | 300^b^ | 449^b^ | 1220 | 11 |
| 9 | Southwest rivers | 280 | Manwan | 100.45 | 24.62 | 920 | 36.9 | - | 1670^b^ | 6664^b^ | 1214 | 45 |
| 9 | Southwest rivers | 840 | Nalan | 103.20 | 22.72 | 261 | 5.3 | - | 150^b^ | 598^b^ | 1217 | 6 |
| 9 | Southwest rivers | 16 | Qiezishan Reservoir | 98.81 | 24.51 | 121 | 3.1 | Main | 16 | 66 | 1157 | 4 |
| 9 | Southwest rivers | 823 | Ruilijiang | 97.68 | 23.86 | 27 | 1.1 | - | 600^b^ | 3050^b^ | 1223 | 1.35 |
| 9 | Southwest rivers | 281 | Sankuaishi | 99.21 | 24.65 | 25 | 3.3 | - | - | - | 1163 | 4 |
| 9 | Southwest rivers | 841 | Tukahe | 102.14 | 22.63 | 78 | 2.9 | - | 165^b^ | 566^b^ | 1216 | 4 |
| 9 | Southwest rivers | 98 | Xiaowan | 100.10 | 24.68 | 15100 | 166.4 | Main | 4200^b^ | 14379^b^ | 1196 | 199 |
| 9 | Southwest rivers | 860 | Xucun | 100.04 | 25.58 | 74 | 2.5 | - | 85.8^b^ | 345^b^ | 1220.7^a^ | 3 |
| 9 | Southwest rivers | 59 | Yamzho Yumco | 90.38 | 29.10 | 42 | 566.9 | Main | 113 | 92 | 1048 | 594 |
| 9 | Southwest rivers | 842 | Yayangshan | 101.74 | 22.92 | 247 | 6.8 | - | 120^b^ | 366^b^ | 1204 | 8 |
| 9 | Southwest rivers | 334 | Zibihe | 99.95 | 26.14 | 93 | 7.9 | - | - | - | 1112 | 9 |
| 10 | Northwest rivers | 113 | Chahanwusu | 86.48 | 42.08 | 125 | 2.6 | Main | 309^b^ | 1234^b^ | 1327 | 3 |
| 10 | Northwest rivers | 114 | Jilintai 1 | 82.87 | 43.85 | 2530 | 100.1 | Main | 460^b^ | 1547^b^ | 1002 | 100 |
| 10 | Northwest rivers | 859 | Jilintai 2 | 82.99 | 43.81 | 4 | 0.2 | - | 50^b^ | 314^b^ | 1010 | 0.20 |
| 10 | Northwest rivers | 209 | Kalangguer | 83.19 | 47.00 | 40 | 2.0 | Sec | 3 | 10 | 1023 | 2 |

**Table S7 Measured evaporation (*E*_m_) for 69 reservoirs and comparison between measured and simulated reservoir evaporation. Reservoirs are listed in order from largest to smallest installed hydroelectric capacity.**

| Reservoir Name | Longitude (°E) | Latitude (°N) | Installed Hydroelectric Capacity [MW] | Hydroelectric generation [GWh yr^-1^] | Representative meteorological station | *E*_m_^a^  [mm yr^-1^] | *E*_IIASA_^b^ [mm yr^-1^] | RE^c^ | Ref. for *E*_m_^a^ |
| --- | --- | --- | --- | --- | --- | --- | --- | --- | --- |
| Three Gorges Dam | 111.00 | 30.83 | 18200 | 84700 | Yichang | 978 | 985 | 0.7% | 1 |
| Laxiwa | 101.18 | 36.07 | 4200 | 10230 | Longyangxia | 1100 | 987 | -10.3% | 2 |
| Ertan | 101.78 | 26.82 | 3300 | 17040 | Ertan | 1270 | 1203 | -5.3% | 3 |
| Gezhouba | 111.26 | 30.74 | 2715 | 15700 | Gezhouba | 753 | 1032 | 37.0% | 4 |
| Xiaolangdi | 112.36 | 34.92 | 1800 | 5900 | Xiaolangdi | 1560 | 1173 | -24.8% | 5 |
| Jinghong | 100.78 | 22.05 | 1750 | 6176 | Jinghong | 1162 | 1283 | 10.4% | 6 |
| Shuikou | 118.81 | 26.31 | 1400 | 61540 | Gutian | 1000 | 1091 | 9.1% | 7 |
| Dachaoshan | 100.36 | 24.05 | 1350 | 6225 | Linchang | 1184 | 1220 | 3.0% | 6 |
| Longyangxia | 100.92 | 36.12 | 1280 | 5940 | Longyangxia | 1100 | 988 | -10.2% | 2 |
| Yantan | 107.51 | 24.05 | 1210 | 6038 | Yantan | 1646 | 1176 | -28.5% | 8 |
| Tianshengqiao 1 | 105.10 | 24.94 | 1200 | 5200 | Tianshengqiao 1 | 1085 | 1134 | 4.5% | 9 |
| Danjiangkou | 111.49 | 32.56 | 900 | 3830 | Danjiangkou | 1000 | 1107 | 10.7% | 10 |
| Ankang | 108.88 | 32.60 | 800 | 2857 | Ankang | 748 | 974 | 30.2% | 11 |
| Fengman | 126.69 | 43.72 | 724 | 1890 | Fengman | 1300 | 987 | -24.1% | 12 |
| Xinanjiang | 119.21 | 29.49 | 663 | 1861 | Xinanjiang | 1215 | 1181 | -2.8% | 13 |
| Lubuge | 104.54 | 24.82 | 600 | 2850 | Lubuge | 1040 | 1132 | 8.9% | 8 |
| Liujiaxia | 103.34 | 35.93 | 570 | 5580 | Liujiaxia | 1000 | 957 | -4.3% | 2 |
| Yanguoxia | 103.27 | 36.06 | 440 | 2240 | Yanguoxia | 1100 | 963 | -12.4% | 2 |
| Qingtongxia | 105.99 | 37.89 | 302 | 1204 | Qingtongxia | 1300 | 1087 | -16.4% | 2 |
| Xinfengjiang | 114.65 | 23.73 | 293 | 990 | Xinfeng | 852 | 1278 | 49.9% | 14 |
| Longma | 102.02 | 23.54 | 285 | 959 | Yuanjiang | 1680 | 1226 | -27.0% | 6 |
| Sanmenxia | 111.34 | 34.83 | 250 | 1310 | Sanmenxia | 1200 | 1120 | -6.7% | 2 |
| Lushui | 113.89 | 29.68 | 240 | 12740 | Lushui | 936 | 1122 | 19.8% | 15 |
| Gutian | 118.80 | 26.57 | 186 | 915 | Gutian | 951 | 1120 | 17.8% | 16 |
| Ansha | 117.11 | 26.03 | 115 | 614 | Hydrological station in Fujian | 900 | 1174 | 30.5% | 16 |
| Chitan | 117.12 | 26.71 | 100 | 500 | Hydrological station in Fujian | 817 | 1127 | 38.0% | 16 |
| Miyun | 116.84 | 40.49 | 96 | 115 | Miyun | 1316 | 1225 | -6.9% | 7 |
| Xucun | 100.04 | 25.58 | 86 | 345 | Dali | 1221 | 1143 | -6.3% | 6 |
| Nanshui | 113.21 | 24.79 | 79 | 258 | Nanshui | 919 | 1130 | 22.9% | 14 |
| Shangyoujiang | 114.40 | 25.84 | 72 | 290 | Shangyoujiang | 1503 | 1118 | -25.6% | 13 |
| Chongjianghe | 99.80 | 27.55 | 70 | 193 | Zhongdian | 1016 | 1030 | 1.4% | 6 |
| Lvshuihe | 103.37 | 23.37 | 66 | 248 | Mengzi | 1449 | 1155 | -20.3% | 6 |
| Guxian | 111.28 | 34.24 | 60 | 97 | Guxian | 950 | 1088 | 14.6% | 2 |
| Shanmei | 118.41 | 25.17 | 60 | 130 | Shanmei | 1767 | 1242 | -29.7% | 13 |
| Luosiwan | 99.74 | 27.83 | 60 | 236 | Zhongdian | 1016 | 1019 | 0.3% | 6 |
| Shitouhe | 107.65 | 34.17 | 50 | 51 | Shitouhe | 800 | 929 | 16.2% | 2 |
| Wananxi | 117.06 | 25.40 | 45 | 136 | Hydrological station in Fujian | 1000 | 1165 | 16.5% | 16 |
| Guanting | 115.60 | 40.23 | 30 | 90 | Guanting | 1111 | 1131 | 1.7% | 17 |
| Taolinkou | 119.05 | 40.14 | 20 | 63 | Taolinkou | 1019 | 1198 | 17.6% | 18 |
| Huangbizhuang | 114.30 | 38.25 | 16 | - | Huangbizhuang | 1239 | 1216 | -1.9% | 19 |
| Changtan | 121.06 | 28.61 | 15 | 55 | Changtan | 1129 | 1009 | -10.7% | 13 |
| Fenhe | 111.91 | 38.05 | 13 | 11 | Fenhe | 1000 | 1097 | 9.7% | 2 |
| Xidayang | 114.78 | 38.75 | 12 | 40 | Xidayang | 983 | 1198 | 21.8% | 18 |
| Luhun | 112.18 | 34.20 | 10 | 10 | Luhun | 990 | 1142 | 15.4% | 20 |
| Tangxi | 116.87 | 23.90 | 8 | 23 | Tangxi | 1457 | 1256 | -13.8% | 13 |
| Hongshan | 119.70 | 42.75 | 7 | 10 | Hongshan | 1054 | 1202 | 14.0% | 21 |
| Qingshan | 114.02 | 29.43 | 7 | 79 | Qingshan | 1196 | 1107 | -7.4% | 13 |
| Dongzhen | 118.98 | 25.48 | 6 | 29 | Dongzhen | 1547 | 1229 | -20.5% | 13 |
| Hengjin | 120.46 | 29.25 | 6 | 20 | Hengjin | 1320 | 1108 | -16.0% | 13 |
| Zhaikou | 110.78 | 34.38 | 5 | 14 | Zhaikou | 1000 | 1003 | 0.3% | 2 |
| Zhuzhuang | 114.19 | 37.01 | 4 | - | Zhuzhuang | 1133 | 1199 | 5.8% | 18 |
| Wangjiachang | 111.52 | 29.77 | 4 | - | Wangjiachang | 1075 | 1041 | -3.2% | 13 |
| Wenyuhe | 112.02 | 37.52 | 3 | 8 | Wenyuhe | 900 | 1125 | 25.0% | 2 |
| Bajiazui | 107.50 | 35.69 | 2 | 4 | Bajiazui | 1000 | 1057 | 5.7% | 2 |
| Yanghe | 119.20 | 39.98 | 2 | 4 | Yanghe | 929 | 1183 | 27.4% | 18 |
| Shilianghe | 118.86 | 34.77 | 1 | 1 | Shilianghe | 1682 | 1163 | -30.8% | 22 |
| Wangyao | 109.09 | 36.90 | 1 | 2 | Wangyao | 900 | 1084 | 20.4% | 2 |
| Shixiakou | 105.91 | 36.83 | - | - | Shixiakou | 1300 | 1009 | -22.4% | 2 |
| Fengjiashan | 107.19 | 34.54 | - | 8.15 | Fengjiashan | 800 | 949 | 18.7% | 2 |
| Yangmaowan | 108.05 | 34.54 | - | - | Yangmaowan | 800 | 979 | 22.4% | 2 |
| Lincheng | 114.39 | 37.44 | - | - | Lincheng | 1008 | 1210 | 20.1% | 18 |
| Wangkuai | 114.51 | 38.74 | - | 13.70 | Wangkuai | 1265 | 1199 | -5.2% | 23 |
| Qiuzhuang | 118.15 | 40.00 | - | - | Qiuzhuang | 1000 | 1230 | 23.0% | 24 |
| Youyi | 114.05 | 40.86 | - | - | Youyi | 1100 | 1138 | 3.4% | 25 |
| Hedi | 110.30 | 21.72 | - | - | Hedi | 1407 | 1271 | -9.7% | 13 |
| Tingxi | 118.14 | 24.80 | - | - | Tingxi | 1495 | 1266 | -15.3% | 13 |
| Dahuofang | 124.10 | 41.89 | - | - | Dahuofang | 843 | 1066 | 26.4% | 26 |
| Erlongshan | 124.79 | 43.19 | - | - | Siping | 797 | 1058 | 32.9% | 27 |
| Donghu | 112.21 | 22.01 | - | - | Donghu | 1006 | 1246 | 23.9% | 26 |
| ^a^Measured reservoir evaporation; ^b^Simulated reservoir evaporation from IIASA^28^; ^c^Relative error (*RE*) = [(*E*_s_ – *E*_m_) / *E*_m_] × 100%. | | | | |  |  |  |  |  |

**Table S8. Allocation coefficient for 26 representative reservoirs in China.**

| Reservoir name | Basin no. | Basin name | Electricity generation as the primary or secondary purpose | Total economic revenues in 2008 | Income from hydroelectricpower generation in 2008 | Allocation coefficient [η] | References for economic values |
| --- | --- | --- | --- | --- | --- | --- | --- |
|  |  |  |  | (×10^6^ RMB) | (×10^6^ RMB) |  |  |
| Bapanxia | 4 | Yellow River | Primary use | 375 | 277.5 | 0.74 | 29 |
| Danjiangkou | 6 | Yangtze River | Primary use | 1358.7 | 1019 | 0.75 | 30,31 |
| Ertan | 6 | Yangtze River | Primary use | 3136.8 | 2841.5 | 0.91 | 32 |
| Gezhouba | 6 | Yangtze River | Primary use | 4686.7 | 4321.1 | 0.92 | 33 |
| Liujiaxia | 4 | Yellow River | Primary use | 2595.5 | 1484.6 | 0.57 | 34 |
| Longyangxia | 4 | Yellow River | Primary use | 2486 | 1839.8 | 0.74 | 29 |
| Lubuge | 8 | Zhujiang | Primary use | 7518.8 | 627.4 | 0.08 | 35 |
| Three Gorges Dam | 6 | Yangtze River | Primary use | 31177.1 | 22447.5 | 0.72 | 36 |
| Tianshengqiao 1 | 8 | Zhujiang | Primary use | 4768.4 | 1383.5 | 0.29 | 37 |
| Tongjiezi | 6 | Yangtze River | Primary use | 1157.2 | 854.1 | 0.74 | 35 |
| Wuqiangxi | 6 | Yangtze River | Primary use | 21518.3 | 1583.3 | 0.07 | 38 |
| Xinfengjiang | 8 | Zhujiang | Primary use | 729.6 | 189.7 | 0.26 | 39 |
| Dawangtan | 8 | Zhujiang | Secondary use | 21.1 | 1.2 | 0.06 | 40 |
| Daxia | 4 | Yellow River | Secondary use | 782.9 | 389.8 | 0.5 | 41 |
| Dongzhen | 7 | Southeast rivers | Secondary use | 14.9 | 7.9 | 0.53 | 42 |
| Erlongshan | 2 | Liaohe | Secondary use | 2580.1 | 46.3 | 0.02 | 43 |
| Fenhe | 4 | Yellow River | Secondary use | 5.9 | 3 | 0.51 | 44 |
| Gangnan | 3 | Haihe | Secondary use | - | - | 0.09 | 45 |
| Gaoyan | 6 | Yangtze River | Secondary use | 12 | 3.2 | 0.27 | 38 |
| Guanting | 3 | Haihe | Secondary use | - | - | 0.09 | 45 |
| Miyun | 3 | Haihe | Secondary use | 749 | 41.9 | 0.06 | 46 |
| Wanjiazhai | 4 | Yellow River | Secondary use | 831.4 | 731.7 | 0.88 | 35 |
| Xiaolangdi | 4 | Yellow River | Secondary use | 4347.3 | 1382.5 | 0.32 | 47 |
| Xidayang | 3 | Haihe | Secondary use | - | - | 0.09 | 45 |
| Yanguoxia | 4 | Yellow River | Primary use | 857.5 | 634.6 | 0.74 | 29 |
| Yuecheng | 3 | Haihe | Secondary use | - | - | 0.09 | 45 |

**References for Table S7 and Table S8:**

1 Zhang, Y. & Li, H. Spatial and temporal distribution of water surface evaporation in Yichang. *Yangtze River***37**, 30-31 (2006).(in Chinese)

2 Tian, J., Cui, Q., Xu, J. & Zhou, X. Surface evaporation of large and middle reservoirs affects the amount of water resource in the Yellow River valley. *J. Shandong Agric. Univ. (Nat. Sci.)* **36**, 391-394 (2005).(in Chinese with English abstract)

3 Guo, R., Liu, S. & Liu, C. Xiaodeshi station water surface evaporation observations in Yalongjiang River. *Sichuan Water Power***1986**, 3-9.(in Chinese)

4 Zhang, Y., Niu, L. & Fan, Y. Calculation and analysis of the amount of water evaporation in Gezhouba reservoir after impoundment. *Hydrology***20**, 32-35 (2000).(in Chinese)

5 Zhang, Y. *et al.* Fog days variation characteristics of Xiaolangdi reservoir before and after impoundment. *Meteorol. Environ. Sci.* **32**, 268-271 (2009). (in Chinese with English abstract)

6 Huang, Y., Fang, S. & Wang, Y. *Water Surface Evaporation Comparison on Calculation Methods in Yunnan*. pp. 165-172 (China Water and Power Press, Beijing,2003).(in Chinese)

7 Gao, H. Spatial and temporal variation of water surface evaporation in Miyun Reservoir. *Beijing Waterworks***2010**,28-30.(2010) (in Chinese)

8 Mekonnen, M. & Hoekstra, A. The blue water footprint of electricity from hydropower.*Hydrol. Earth Syst. Sci.* **16**, 179-187 (2012).

9 Lei, X., Lin, Z., Su, Z., Zhou, S. & Huang, M. Characteristics of climate change and impact analysis in Tianshengqiao No. 1 reservoir. *J. Anhui Agric. Sci.* **38**, 3556-3558, 3653 (2010).(in Chinese)

10 Geng, W. *Study on the Quantity of Removal Water of the Dan Jiang Kou Reservoir.*(Zhengzhou University, Zhengzhou, 2007). (in Chinese with English abstract)

11 Wang, N., Sun, X., Cai, X. & Wang, Q. Characteristics of climate change in Ankang Reservoir upstream basin before and after impoundment. *Meteorol.Sci. Technol.* **38**, 649-654 (2010). (in Chinese with English abstract)

12 Cai, W. Discussion of water affairs ofFengman powerplant. *J. Hydroelec. Eng.* **57**, 67-74 (1997).(in Chinese with English abstract)

13 Ni, Z. Determination of reservoir surface evaporation. *Water Power***1982**,40-45.(1982) (in Chinese)

14 Wen, X. & Liu, J. Evaporation capacity and drought index characteristic analysis in Shaoguan City. *Guangdong Water Resour. Hydropower***2010**, 39-41.(2010) (in Chinese)

15 Wu, F. & Mao, R. Ecological and environmentalassessment of the Lushui reservoir. *Yangtze River***25**, 45-50 (1994).(in Chinese with English abstract)

16 Wu, C. Spatial and temporal distribution of the evaporation and the interannual variability analysis in Fujian Province. *Water Sci. Technol.* **2004**, 3-5.(in Chinese)

17 Wang, J. & Wang, B. Monthly temperature and water surface evaporation analysis in Xinjiang province. *Arid LandGeogr.***11**, 2-10 (1988).(in Chinese with English abstract)

18 Qi, C. & Wang, C. Analysis on water surface evaporation data in Hebei Province. *Hebei Water Resour. Hydropower Eng.* **2003**, 37-38.(in Chinese)

19 China Association for Science and Technology. *Reservoir.* Available at:<http://baike.baidu.com/view/104597.htm> (2013) (in Chinese)

This web site provides a detailed introduction of the Huangbizhuang Reservoir (Reservoir ID: 748). In this paper, we use the measured annual evaporation data (1239 mm/yr) for the Huangbizhuang Reservoir, which is included in Table S7.

20 Wang, D., Chai, P. & Li, J. Water surface evaporation observation and surface evaporation law in Yellow River Basin. *Yellow River***2**, 19-20 (1996).(in Chinese)

21 Li, Y. & Bi, K. Water surface evaporation analysis and calculation in Hongshan Reservoir. *Water Resour. Hydropower Northeast China***57**, 33-36 (1989).(in Chinese)

22 Chen, J. & Xiang, Y. *Climate change and characteristics of reservoirs in 50 years in Jiangsu Province*. *6^th^ Yangtze River DeltaMeteorol.Forum***2009**, 44-49.(2009) (in Chinese)

23 Haihe river water resources commission. *An introduction to Wangkuai reservoir.* Available at:http://www.hwcc.gov.cn/pub/hwcc/wwgj/jishupd/liuyuzdgc/sk/tnull_76033.html (2013) (in Chinese)

This web site is maintained by the Haihe River Water Conservancy Commission, Ministry of Water Resources of China, and it provides a detailed introduction of the Reservoir Wangkuai (Reservoir ID: 750). In this paper, we use the measured annual evaporation data (1239 mm/yr) for Reservoir Wangkuai, which is included in Table S7.

24 China Association for Science and Technology*.Qiuzhuang Reservoir.* Available at:<http://baike.baidu.com/view/1619355.htm> (2013) (in Chinese)

This web site provides a detailed introduction of the Qiuzhuang Reservoir (Reservoir ID: 757). In this paper, we use the measured annual evaporation data (1239 mm/yr) for the Qiuzhuang Reservoir, which is included in Table S7.

25 China Association for Science and Technology*.Youyi Hydropower Station.* Available at:<http://baike.baidu.com/view/1619342.htm> (2013) (in Chinese)

This web site provides a detailed introduction of the Youyi Reservoir (Reservoir ID: 772). In this paper, we use the measured annual evaporation data (1100 mm/yr) for the Youyi Reservoir, which is included in Table S7.

26 Ni, Z. Floating evaporation data with the reservoir lake evaporation. *Hydroelec.Power***1991**, 44-48.(1991) (in Chinese)

27 Gao, Y. The rules of water surface evaporation of Jilin Province at central and western regions. *China-ASEAN Expo***8**,88 (2011).(in Chinese)

28 IIASA. Global map of monthly reference evapotranspiration—30 arc minutes (IIASA, Laxenburg, 2000).

29 Sun, H. & Fang, R. Benefit analysis of hydropower stations on the upper Yellow River. *Northwest Water Power***74**, 1-5, 52 (2000). (in Chinese with English abstract)

30 Huang, B. & Zhou, D. Preliminary analysis of Danjiangkouhydropower station and the operation of power generation benefit. *Yangtze River***1982**, 19-28. (1982) (in Chinese)

31 Guan, R. Danjiangkou hydropower generation benefit analysis. *Hubei Hydroelec.Power***1989**, 47-50.(1989) (in Chinese)

32 North Pole Star Network. *Analysising the hdyropower profit model.* Available at:<http://news.bjx.com.cn/html/20120326/350238-3.shtml> (2013) (in Chinese)

This web site provides the main revenue sources of the Ertan hydropower station (Reservoir ID: 42). In this paper, we use the total economic revenues data for the Ertan hydropower station, which is included in Table S8.

33 China Yangtze Power Co., LTD. *An introduction to Gezhouba Hydropower Station in Yangtze River.*Available at:<http://wenku.baidu.com/view/d931504de518964bcf847cd6.html> (2013) (in Chinese)

This web site provides the main revenue sources of the Gezhouba hydropower station (Reservoir ID: 71). In this paper, we use the total economic revenues data for the Gezhouba hydropower station, which is included in Table S8.

34 Chen, H. & Wang, S.Glory and dream.*Gansu Daily* (2004/04/25). (in Chinese)

This web site provides a news report on the Liujiaxia hydropower station (Reservoir ID: 83). In this paper, we use the total economic revenues data for the Liujiaxia hydropower station, which is included in Table S8.

35 Bing, F. *Almanac of China's Water Power* (China Electric Power Press, Beijing, 2010). (in Chinese)

36 Qiu, Z. & Tan, C. Resettlement planning and designing of Three Gorges Project of the Yangtze River. *Yangtze River***34**, 43-46, 66 (2003). (in Chinese with English abstract)

37 Shi, S. The evaluation of economic value in Ertan hydropower station.(China Electric Power Press, Beijing, 1987). (in Chinese)

38 People’s Government of Yongzhou City. *An introduction to Gaoyan reservoir.* Available at:<http://www.yzcity.gov.cn/art/2005/12/9/art_2996_158862.html> (2013) (in Chinese)

This web site provides the annual income data for the Gaoyan Reservoir (Reservoir ID: 156). In this paper, we use the total economic revenues data for the Gaoyan Reservoir, which is included in Table S8.

39 Ling, G. Dongjiang River basin investment share three reservoir method discussed in this paper. *Jilin Water Conserv.***313**, 63-66 (2008). (in Chinese)

40 Ning, C. Thinking for the sustainable development of Dawangtan reservoir. *Guangxi Water Resour.Hydropower***3**, 48-50 (1998). (in Chinese with English abstract)

41 Baidu Company. *Economic report of Daxia Hydropower Station.* Available at:<http://wenku.baidu.com/view/003b2e0ecc175527072208ca.html> (2013) (in Chinese)

This web site provides the annual income data for the Daxia Hydropower Station (Reservoir ID: 187). In this paper, we use the total economic revenues data for the Daxia Hydropower Station, which is included in Table S8.

42 Dongzhen reservoir authority in Putian City. *An introduction to Dongzhen reservoir..* Available at :<http://www.ptdzsk.cn/Index/Display.asp?NewsID=100300> (2013) (in Chinese)

This web site provides the annual income data for the Dongzhen Reservoir (Reservoir ID: 155). In this paper, we use the total economic revenues data for the Dongzhen Reservoir, which is included in Table S8.

43 Cui, W. & Liu, C. Conscientiously implement scientific dispatching reservoir benefits into full play. *Jilin Water Conserv.***4**, 17-19 (1995). (in Chinese)

44 Shanxi water conservancy department. *An introdocution to Fenhe reservoir.* Available at:<http://www.sxwater.gov.cn/home/details.asp?articleid=7941> (2013) (in Chinese)

This web site provides the annual income data for the Fenhe Reservoir (Reservoir ID: 191). In this paper, we use the total economic revenues data for the Fenhe Reservoir, which is included in Table S8.

45 Yuan, Z. & Zeng, Y. A preliminary analysis of Haihe River basin, some large reservoir benefit. *Haihe River Water Conserv.***4**, 24-25 (1984). (in Chinese)

46 Zheng, J. Miyun reservoir project brief comprehensive economic benefit. *Beijing Water Conserv.***6***,*43(1999). (in Chinese)

47 Shi, A. & Zheng, C. A forecasting analysis study on Xiaolangdi water contronl project during operation.*J. Hydroelec. Eng.* **73**,10-15(2001). (in Chinese with English abstract)

**Supplementary Information – Data Sources**

**Appendix I. Data sources for reservoir volume and dam height for reservoirs 776 to 885.**

1 Ai, J. Dahua hydropower station start the construction. *Guiyang Yearbook* **2004**, 454. (2004) (in Chinese)

2 Bai, S., Bai, J., Zhang, X. & Zhang, X. Analysis of dam stability of Gelantan hydropower station. *Design Water Resour. Hydroelec. Eng.* **28**, 15-18 (2009). (in Chinese)

3 Bing, F. *Design of Jinghong Hydropower Station Left Bank Abutment of Anchor Plate and Seepage Control*. Almanac of China Power pp. 209-211 (China Electric Power Press, Beijing, 2009). (in Chinese)

4 Chen, G. Engineering survey of Doulingzi hydropower station. *Hubei Hydroelec. Power* **67,** 62 (2007). (in Chinese)

5 Chen, G. & Lin, S. Environmental potection measures in Guangzhao hydropower station project. *Guizhou Water Power* **22**, 16-19 (2008). (in Chinese with English abstract)

6 Chen, H. The optimization of construction technology program for Zhaokou hydropower station project. *Water Conserv. Hydropower Construction* **01**, 41-45, 62 (2008). (in Chinese)

7 Chen, J. *Companies Introduction. Shanmei Hydropower Station in Quanzhou City*. Statistical Yearbook of Industrial Economy of Fujian **2000**, 306. (China Statistic Press, Beijing, 2000) (in Chinese)

8 Chen, Q. Construction management of Lengzhuguan hydroelectric plant of Sichuan Huaneng Corporation and its output works. *Design Hydroelec. Power Station* **17**, 1-5 (2001). (in Chinese)

9 Chen, S. The review and summary of technology improvement engineering in Shanmei hydropower station. *Hydraul. Sci. Technol.* **4**, 52-53 (2006). (in Chinese)

10 Cheng, X. *Taoyuan County Lingjintan Hydropower Station is Fully Completed and put Into Operation*. p. 412. (Hunan Yearbook Club, Changsha, 2001). (in Chinese)

11 Deng, Y. & Ji, H. The design summarize of hub layout in Tuka River hydropower station. *Yunnan Water Power* **22**, 4-6 (2006). (in Chinese)

12 Ding, R. Jinjitan hydropower station parameters determination and model selection. *Planning Design Water Conserv.* **4**, 72-74 (2010). (in Chinese)

13 Fan, Y. Appreciation, forecast of impact on ecological environment of Tuanpo hydropower station & its environment protect measures. *Guizhou Water Power* **20**, 11-14 (2006). (in Chinese)

14 Fang, K. *Xuecheng Hydropower Station Project Department 3 Generating Units in Xuecheng Hydropower Station all put Into Production Power Generation*. p. 159. (Editorial Department of Gezhouba Dam Group Yearbook, Hubei, 2008). (in Chinese)

15 Feng, Q., Lu, W., Hu, Z. & Liu, D. Land requisition and resettlement plan for the construction of Pengshui hydropower station *Yangtze River* **37**, 74-76 (2006). (in Chinese)

16 Gao, D. *Other Domestic Engineering Construction Yixing Pumped Storage Power Station’s Principal Part of the Project Start*. *Gezhouba Company Yearbook* **2004,** p. 175. (2004) (in Chinese)

17 Gao, P., Zhang, W. & Liu, J. Design on hydraulic generator set and auxiliary equipment of Gelantan hydropower station. *Design Water Resour. Hydroelec. Eng.* **28**, 42-44 (2009). (in Chinese)

18 Gu, D. & Fan, J. The general planning and deployment program of construction in Wuyiqiao hydropower station. *Design Hydroelec. Power Station* **24**, 102-105 (2008). (in Chinese)

19 Guo, P., Xu, Y. & Zhang, J. The analysis of influence complication(s) and rat-killing effect in the Yellow River Suzhi power plant submerse area. *Chin. J. Pest Control* **25**, 543-544 (2009). (in Chinese with English abstract)

20 Han, L., Wang, Z. & Wu, X. Construction technology of high pressure rotary sprinkling impervious wall in a cofferdam of Yunnan hydropower station. *Explor. Eng. (Rock Soil Drilling Tunneling)* **5**, 24-26 (2007). (in Chinese)

21 Hu, Y., Xu, W. & Zhang, W. *Bapanxia Hydropower Station Comprehensive Index*. p. 393. (Ministry of Water Resources of the Yellow River Water Conservancy Press, Henan, 2010). (in Chinese)

22 Huang, H. The selection of dam site and dam type in Tongtou hydropower station. *Water Power* **2**, 40-41 (1990). (in Chinese)

23 Huang, W. & Ren, K. Research on overall stability of Left Bank Hilly Region in Lianbu hydropower station. *J. Zhejiang Water Conserv. Hydropower Coll.* **21**, 1-6 (2009). (in Chinese with English abstract)

24 Huang, Y. *Yixing Pumped Storage Power Station in Reservoir Construction Key Technology Research*. Nanjing Hydraulic Research Institute, Jiangsu Province (2009-05-08). (in Chinese)

25 Jiang, M. & Xu, P. Application of needle beam steel form Jambo in concrete construction in headrace tunnel at Taishui hydropower station in Jilin province. *Sichuan Water Power* **23**, 115-117 (2004). (in Chinese)

26 Jiang, Z. *Development Project Hongyi Hydropower Station*. Almanac of China Power, Beijing, p. 469. (China Electric Power Press, Beijing, 2008). (in Chinese)

27 Kou, Y. & Cao, Y. The successful application of concrete construction in layered inversion at Liuping hydropower station. *Hydropower Station Design* **27**, 113-118 (2011). (in Chinese)

28 Lei, X. & Luo, B. Longtan hydropower station electrical design. *Intelligence* **24**, 232-233 (2009). (in Chinese)

29 Li, G., Lan, Y. & Liu, G. *Electrical Data Acquisition Device in Yixing Pumped Storage Power Plant Trial Success*. pp. 295-296 (China Electric Power Press, Beijing, 2009). (in Chinese)

30 Li, J. *The Survey of Zhiganglaka Hydropower Station (in Construction)*. p. 431. Yellow River Water Conservancy Commission Yearbook. (Club of Ministry of Water Resources, Zhenzhou, 2004). (in Chinese)

31 Li, M. *The Overall Design of Junctions at the First Pivot Layout of Yingjiang Hydropower Station (Level 4).* pp. 349-352 (China Electric Power Press, Beijing, 2005) (in Chinese)

32 Li, M. & Song, Q. *Design of Hydropower Planning and Design of Yixing Pumped Storage Power Station Water Power*. pp. 217-218 (China Electric Power Press, Beijing, 2001-2002). (in Chinese)

33 Li, T. The construction of water diversion tunnel intake in Wawushan hydropower station. *Water Resour. Hydropower Construc.* **30**, 24-25 (2007). (in Chinese)

34 Li, W. The application of curtain grouting in danger defence and consolidate at Youyi reservoir. *Water Sci. Eng. Technol.* **S1**, 27-28 (2007). (in Chinese)

35 Li, W. & Yang, N. Practice of engineering construction management in Dongqing hydropower station. *Guizhou Water Power* **23**, 1-5 (2009). (in Chinese)

36 Li, Y. *Environmental Protection Of Hydropower Station Impact Assessment of Xiaowan Hydropower Station*. pp. 513-514 (China Electric Power Press, Beijing 2001-2002). (in Chinese)

37 Li, Z. Application of grouting technology in spillway sluice foundation seepage control at Youyi reservoir. *Hebei Water Conservancy Hydropower Technol.* **4**, 35-36 (2004). (in Chinese)

38 Lin, G. & Wang, X. The construction and management of Xiaoguanzi hydropower station in Sichuan huaneng. *Water Power* **2**, 48-49 (2002). (in Chinese)

39 Liu, C. The analysis of present situation and compose of management about water & soil erosion in Qiuzhuang reservoir upstream basin. *Hebei Water Resour.* **5**, 38-39 (2005). (in Chinese)

40 Liu, F. & Liu, Z. Test research on Dongxiguan hydropower station dam energy dissipation layout. *Design Hydropower Station* **11**, 75-81 (1995). (in Chinese)

41 Liu, H. *Development Project Seergu Hydropower Station*. pp. 468-469 (China Electric Power Press, Beijing, 2008). (in Chinese)

42 Liu, J. Bapanxia Yellow River hydropower station expansion machine engineering through the review of preliminary design report. *Northwest Water Power* **3**, 57 (1993). (in Chinese)

43 Liu, J., Liu, B. & Dai, H. The features & difficulties of engineering, and the summary of strategies & measures about supervision work in Shiziping hydropower station. *Sichuan Water Power* **29**, 1-4 (2010). (in Chinese)

44 Liu, S. & Gan, M. Design and running of the blocking dregs dam in Huizhou pumped storage power station. *J. Guangdong Tech. Coll. Water Resour. Elec. Eng.* **7**, 42-46 (2009). (in Chinese with English abstract)

45 Lu, W. & Song, R. A summary about construction technology of the underground powerhouse's system in Muzuo hydropower station. *Construc. Org. Design* **56**, 16-18 (2005). (in Chinese)

46 Luo, L. & Yang, N. Bowl of rice is analysed slope depth of generator leading phase operation of hydropower stations. *Gansu Water Conservancy Hydropower Technol.* **46**, 23-25 (2010). (in Chinese)

47 Luo, X. & Tong, D. The construction design summarize of Yayangshan hydropower station. *Yunnan Water Power,* **19**, 33-38 (2003). (in Chinese)

48 Ma, M. *Tianhuangping Pumped—Storage Power Station Main Equipment Running Status*. pp. 260-261 (China Electric Power Press, Beijing, 2001-2002). (in Chinese)

49 Mu, H., Zhan, Z., Chen, B. & Cai, D. Pivot layout of Dongqing hydropower station project. *Guizhou Water Power* **21**, 37-40 (2007). (in Chinese with English abstract)

50 Nie, G., Cao, X. & Cao, S. Treatment of sick dam in Majitang hydropower station. *Water Power* **31**, 77-79 (2005). (in Chinese with English abstract)

51 Pan, C. & Li, Y. The dam foundation curtain grouting quality assessment of Shweli (1) hydropower project. *Yunnan Water Power,* **24**, 56-60 (2008). (in Chinese)

52 Pan, Y. *Engineering Construction Subject of Building Projects in Dongping Hydropower Station*. p. 613. (China Electric Power Press, Beijing, 2003-2005). (in Chinese)

53 Qiang, Z. *Construction and Development of Tianhuangping Pumped-Storage Power Station Through the National Completion Inspection and Acceptance*. pp. 315-316 (China Electric Power Press, Beijing, 2003). (in Chinese)

54 Ruan, Q. & Wei, A. *Conventional Hydropower Project Introduction of Letan Hydropower Station Project*. pp. 159-160 (China Electric Power Press, Beijing, 2009). (in Chinese)

55 Shang, S. The construction and management of Xucun hydropower station. *Water Resour. Hydropower Eng.* **33**, 23-26 (2002). (in Chinese)

56 Shen, C. Summary on main engineering geological problems of Siling Hydropower Station. *Guizhou Water Power* **2**, 18-20 (2003). (in Chinese)

57 Shi, H. The attempt of hydro-generator modified condenser operation in Wangjiachang hydropower station. *Hunan Water Resour.* **4**, 38-40 (1994). (in Chinese)

58 Shi, Y., Xu, M. & Zeng, L. Engineering geology study method for Ziyili hydroelectric power station air cushion surge chamber. *Design Hydroelec. Power Station* **20**, 81-84 (2004). (in Chinese)

59 Si, Y. *Engineering Construction Taolinkou Reservoir.* p. 164. (China Water and Power Press, Beijing, 1999). (in Chinese)

60 Sichuan Province: Dadu River Basin Hydropower Development Co., Ltd. *The Study of Diversion Tunnel Closing Quickly Under the Brake in Pubugou Hydropower Station*. (Sichuan Province, Dadu River Basin Hydropower Development Co., Ltd., Chengdu, 2010). (in Chinese)

61 Sichuan Province: Dadu River Basin Hydropower Development Co., Ltd. *The Optimization Research of Installed Capacity in Pubugou Hydropower Station*. (Sichuan Province, Dadu River Basin Hydropower Development Co., Ltd., Chengdu, 2006). (in Chinese)

62 Tian, L. Design and research on arrangement of discharge type powerhouse for tubular units of Shuhe hydropower project. *Northwest Water Power* **2**, 24-28 (2011). (in Chinese with English abstract)

63 Wang, B. The project construction and management of Qingju hydropower station. *Sichuan Water Power* **24**, 67-68 (2005). (in Chinese)

64 Wang, M., Shen, M.L. The water diversion and sand defend experimental study of Yinping hydropower station. *Water Resour. Hydropower Northeast China* **24**, 59-61 (2006). (in Chinese)

65 Wang, S. Construction and application of automatic hydrological information acquisition system at Pingban hydropower station. *Guangxi Water Power* **13**, 17-20 (2010). (in Chinese with English abstract)

66 Wei, H., Chao, Y. & Zhao, Y. The technology and practice of Liziping hydropower station diversion system covering layer into the hole. *Sichuan Hydropower* **27**, 33-35 (2008). (in Chinese)

67 Wen, Y. Feilaixia hydropower station economic evaluation analysis. *Guangdong Water Conserv. Hydroelec. Power* **9**, 66-68 (2011). (in Chinese)

68 Wu, S. Karst hydro-geologic characteristics in dam area of Silin hydropower station & treatment measures. *Guizhou Water Power* **19**, 20-24 (2005). (in Chinese)

69 Wu, X. & Shi, Y. Project layout of Pengshui hydropower station and energy dissipating design and research. *Yangtze River* **37**, 29-32 (2006). (in Chinese)

70 Xiang, M. & Liu, X. Discussion of hydroelectric power station electric equipment over-voltage protection measures discussed. *Power Supply Technol. Applic.* **20**, 25-27 (2012). (in Chinese with English abstract)

71 Xu, G. Wujiang River hydropower station wind camp karst leakage survey and key technology research and application. (China Hydropower Consultant Group of Guiyang Survey Design and Research Institute, Guiyang, 2011-05-27). (in Chinese)

72 Xu, P. Construction environment and ecological protection of Dongqing hydropower station project. *Guizhou Water Power* **23**, 5-8 (2009). (in Chinese)

73 Xu, P. & Chen, X. Comprehensive evaluation of pumped storage power station—taking Tongbai pumped storage power station as an example. *J. Guilin Aerospace Industry College* **57**, 53-55 (2010). (in Chinese)

74 Xu, X. The optimization design of coffer dam scheme in Mayandong hydropower station phase II construction. *Water Resour. Hydropower Northeast China* **1**, 18-20 (2012). (in Chinese)

75 Xu, X. & Fan, C. *Overview Xiaowan Hydropower Station in Yunnan*. p. 110. (Yearbook Editorial Department of Gezhouba Dam Group, Yichang, 2010). (in Chinese)

76 Yang, D. The characteristics of Hongshui gully debris flow in Seergu hydropower Station. *Design Hydroelec. Power Station* **22**, 79-81 (2006). (in Chinese)

77 Yang, J. *Xixi River Luogu Hydropower Station Project Department Construction of Concrete Mixing System and Sand Processing in Luogu Hydropower Station.* p. 144. (Yearbook Editorial Department of Gezhouba Dam Group, Yichang, 2007). (in Chinese)

78 Yang, J. & Geng, K. Stability and reinforcement action of the powerhouse back high slope of Jilintai hydropower station. *Hydrogeol. Eng. Geol.* **2**, 41-44 (2004). (in Chinese with English abstract)

79 Yang, W. & Cheng, B. The design of Gucheng hydropower station. *Hydropower New Energy* **5**, 50-53 (2012). (in Chinese with English abstract)

80 Yang, X. *The Lancang River Hydropower Project Construction Jinghong Hydropower Station Excavation Construction Meet the Contract Requirements on Left Bank Entry Road and on the Left Bank*. pp. 160-161 (2004). (Gezhouba Company Yearbook, Yichang, 2004) (in Chinese)

81 Yang, Z. *China's Hydropower Station Engineering of Hydropower Construction*. pp. 254-255 (China Electric Power Press, Beijing, 2003). (in Chinese)

82 Ye, N. Analysis on reasons of abnormal tripping of 220kV high voltage switch in Changzhou hydropower station. *Huadian Technol.* **33**, 16-18 (2011). (in Chinese)

83 Yin, W. Introduced and control analysis of Chahanwusu hydropower station governor system. W*ater Conserv. Hydropower Construct.* **117**, 96-98 (2009). (in Chinese)

84 Yu, C. & Zeng, L. *Other Domestic Engineering Construction Goupitan Hydropower Station Engineering Construction*. p. 187. (Gezhouba Company Yearbook, Yichang, 2003). (in Chinese)

85 Yuan, J. Main channel closure design of Bailongtan hydropower station (II). *Guangxi Elec. Power Eng.* **3**, 21-28 (1997). (in Chinese)

86 Yuan, J. Main channel closure design of Bailongtan hydropower station. *Guangxi Elec. Power Eng.* **36**, 23-25 (1997). (in Chinese)

87 Zeng, G. & Zheng, L. Problems & their solutions in spillway engineering of Dongqing hydropower station.*Guizhou Water Power* **24**, 50-52 (2010). (in Chinese)

88 Zeng, J. *Study on the Mechanism, Stability and Effect to the Accumulation Located in the Left Bank of the Lianbu hydropower Dam in the Xixi River Valley*. (Chengdu University of Technology, Chengdu, 2008). (in Chinese with English abstract)

89 Zeng, Q. *Conventional Hydropower Project the Construction Situation of Jiemian Hydropower Station*. p. 307. (China Electric Power Press, Beijing, 2008). (in Chinese)

90 Zeng, X. Experience of Baishiyao hydropower station engineering flood-fighting and emergency rescues. *People of the Pearl River* **4**, 30-32 (1994). (in Chinese)

91 Zhang, H. *The Electric Power Industry: Determining Xiaowan Hydropower Station Dam Site*. p. 201. (Yunnan Yearbook, Kunming, 1992). (in Chinese)

92 Zhang, H. The study of shear strength for engineering rock Massin Nalan hydropower station dam area. *Yunnan Water Power* **18**, 18-20 (2002). (in Chinese)

93 Zhang, J. Youyi reservoir in the city of Zhangjiakou. *Hebei Water Resour.* **10**, 50 (2009). (in Chinese)

94 Zhang, Q. & He, L. The layout design of powerhouse in Shanxiu hydropower station. *Water Resour. Planning Design* **4**, 45-48 (2010). (in Chinese)

95 Zhang, X. & Pan, C. Controlling curtain grouting technology in the application of earth-rock weir in hydropower station downstream flood home. *China New Technol. Prod.* **8**, 19-20 (2010). (in Chinese)

96 Zhang, Y. Hongye secondary hydropower stations connected to the grid. *J. Southwest Elec. Power* **01**, (2002-11-03). (in Chinese)

97 Zhang, Z. Yili River hydroelectric power station generates electricity in the 31 years. *Yunnan Water Power* **4**, 10-15 (1989). (in Chinese)

98 Zhang, Z. & Fan, J. The application of flood dispatching system in flood control of Qiuzhuang reservoir. *South-to-North Water Diversion Water Sci. Technol.* **3,** 39-40 (2005). (in Chinese with English abstract)

99 Zhao, B., Li, L. & Wei, F. Research on water resources justification for construction project of Shuhe hydropower on the Hanjiang River. *Water Conserv. Sci. Technol. Econ.* **17**, 77-80 (2011). (in Chinese with English abstract)

100 Zhao, W. Determination of the scale and project benefit of Suzhi hydropower station. *Northwest Hydropower* **2**, 1-3 (2008). (in Chinese with English abstract)

101 Zheng, C. & Li, T. Exploitation on changing direction rotation in unit design of Muzuo water power station. *Sichuan Water Resour.* **5**, 31-32 (2009). (in Chinese)

102 Zhou, C. & Li, Y. Research of the method for determining Hongshan reservoir water surface evaporation. *Inner Mongolia Water Resour.* **2**, 8-11 (1998). (in Chinese)

103 Zhou, W. *Xixi River Luogu Hydropower Station Engineering*. pp. 158-159 (Yearbook Editorial Department of Gezhouba Dam Group, Yichang, 2008). (in Chinese)

104 Zhou, Z. Discuss about optimal operation of the unit at Zhuzhuang hydropower station. *Small Hydropower* **1**, 57-59 (2009). (in Chinese)

105 Zhu, G. Bringing forth new ideas in practice—some features in Dongxiguan hydroelectric plant design. *Design Hydroelec. Power Station* **12**, 7-11 (1996). (in Chinese with English absrtact)

**Appendix II. Data sources for hydroelectric power generation by some reservoirs**

1 Chen, B. The grass earth cofferdam in Nahe hydropower station. *Yangtze River* **28**, 18-19 (1997). (in Chinese)

2 Cui, L. West ocean power plant No. 4 generator on the guide and thrust bearing temperature rise. *China's Rural Water Resour. Hydropower* **3***,* (2001). (in Chinese)

3 Dai, L. Fengdu hydropower station dam site downstream I on the left bank landslide stability analysis. *Water Conserv. Hydropower Technol.* **6,** (1986). (in Chinese)

4 Du, B. A bright pearl on the river—FengJiashan hydropower station. *Shanxi Hydroelec. Power* **9,** 22 (1993). (in Chinese)

5 Fu, S. Analysis on the technical transformation of the first unit in Chaihe Reservoir-type power plant. *China's Hydropower Electrification* **77**, 63-66 (2011). (in Chinese with English abstract)

6 Gao, Y. Fenhe hydropower station safety management practice and experience. *Shaanxi Water Conserv.* **4**, 56-57 (2009). (in Chinese)

7 Guo, L., Dai, D., Liu, W. & Qu, H. Zhaikou hydropower station technical transformation analysis. *China's Hydropower Electrification* **9**, 52-54 (2006). (in Chinese)

8 Li, G. Ming lake pumped storage power station. *Water Conserv. Hydropower Lett.* **17**, 18 (1996). (in Chinese)

9 Li, W., Zhong, C. & Chen, T. The quality analysis and evaluation of Mingyangguan reservoir dam. *Water Conserv. Tech. Supervision* **2**, 31-34 (2003). (in Chinese)

10 Lin, B. & Song, Y. Huamuqiao hydropower station high pressure steel pipe and steel bifurcation pipe of the prototype observation. *Hydroelec. Power* **108**, 45-50 (1987). (in Chinese)

11 Liu, C. Jiangya hydropower station, mechanical and electrical installation project construction practice. *Hydroelec. Power* **7**, 63-65 (1999). (in Chinese)

12 Liu, G. Yunshui should be more reasonable scheduling of cascade hydropower stations to generate electricity. *Hydroelec. Power* **10**, 53-54 (1983). (in Chinese)

13 Liu, S., Wu, X. & Wang, H. Composite membrane used in determine the dam seepage control panel application. *Hunan Water Resour. Hydropower* **3**, 1-2 (2009). (in Chinese)

14 Mao, J., Wang, B. & Chai, W. Luhun reservoir increase transformation efficiency. *Small Hydropower* **4**, 39-40 (2000). (in Chinese)

15 Mao, Z. Fengshuling hydropower station construction project target management produced results. *Hydroelec. Power* **3**, 57-58 (1992). (in Chinese)

16 Mu, J. FUR portfolio protection device in the application of the auxiliary power system of Jiangya hydropower station. *Hydroelec. Power* **31**, 61-63 (2005). (in Chinese with English abstract)

17 Pan, L. *Water resources argumentation of Laiwu power plant analysis*. (Shandong University, Jinan, 2010). (in Chinese with English abstract)

18 Qi, T. & Cai, J. Small hydroelectric power station electric equipment of the common electrical failure and its elimination method—fallen snow where hydropower station as an example. *China's Water Conserv.* **8**, 69-70 (2007). (in Chinese)

19 Qian, S. Regional economic development must speed up power construction. *Hydroelec. Power* **5**, 3-4 (1995). (in Chinese)

20 Qiu, P. Self-excited generator switch trip after antihypertensive measures. *Small Irrigation Water Conserv. Hydroelec. Power* **10**, 40-41 (1987). (in Chinese)

21 Shen, Z. & Liu, S. Water diversion system of Yanghe reservoir power plant technical transformation. *Hebei Province Water Conserv. Hydropower Technol.* **1**, 36 (1998). (in Chinese)

22 Shu, Y. Taiwan Ming lake pumped storage power station. *East China Elec. Power* **10**, 52 (1994). (in Chinese)

23 Song, E. The Utilization of Water Quantity and Analysis on Power Output for Large or Medium Hydraulic Power Stations in Northeast China. *Northeast Elec. Power Technol.* **7**, 7-12 (2005). (in Chinese)

24 Sun, Z. & Tan, X. Baipenzhu plant thrust bearing burning tile processing. *Hydropower Station Mech. Elec. Technol.* **4**, 43 (1986). (in Chinese)

25 Tang, Y., Ma, Y., Yang, J. & Li, R. Baihe hydropower station arch dam shape optimization design. *Water Conserv. Hydropower Technol.* **30**, 13-16 (1999). (in Chinese)

26 Tao, Q. Yunnan–Guizhouxiangshui hydropower station water wheel generator structure and characteristics. *Hydropower Station Mech. Elec. Technol.* **1**, 10-11 (2003). (in Chinese)

27 Tian, X., Xu, Y., Liu, B. & Hu, G. Dalongdong put water tower reconstruction of surface water reservoir structure choice. *China's Rural Water Resour. Hydropower* **10**, 43-45 (2001). (in Chinese)

28 Wang, S. Experimental investigation on concrete creep deformation for overflow dam of Daduan hydroelectric station. *Jiangxi Province Water Conserv. Sci. Technol.* **18**, 248-253 (1992). (in Chinese with English abstract)

29 Wen, Y. Feilaixia hydropower plant generates economic evaluation analysis. *Guangdong Water Conserv. Hydropower* **9**, 66-68 (2011). (in Chinese)

30 Wu, S., Zhang, D., Liu, Y. & Hong, L. Baiguishan reservoir economic benefit analysis of water supply. *Water Conserv. Econ.* **21**, 54-56 (2003). (in Chinese)

31 Wu, X. Hongmen hydropower station, the main dam security processing. *Labor Protection Hydraul. Elec. Eng.* **3**, 26-28 (2002). (in Chinese)

32 Xu, J. Baiyun hydropower station is completed and put into production. *Yangtze River* **3,** 16 (2001). (in Chinese)

33 Xu, Y., Guo, Z. & Sun, C. Yuxi plant imported turbine generating unit speed characteristics test and analysis. *Zhejiang Water Conserv. Sci. Technol.* **2**, 35-38 (1999). (in Chinese)

34 Xue, H. Elastic metal fluorine plastic tile in stone and application effect of water power station. *Shanxi Water Conserv.* **1**, 31-32 (2002). (in Chinese)

35 Xue, Q., Yang, Y. & Tang, W. Guxian hydropower station optimal operation mode is discussed. *Xiyan Huang (*2002), 189-206. (in Chinese)

36 Yuan, S. Dahongshan plant prevent the deluge of governor oil pump motor phase running measures. *Hydroelec. Power* **1**, 58 (1987). (in Chinese)

37 Zhang, C., Yu, P. & Yang, J. Nanwan hydropower plant technology reform. *Henan Water Conserv.* **6***,* (2002). (in Chinese)

38 Zhang, J. West ocean power plant unit 1 of capacity reform practice. *Hebei Province Water Conserv. Hydropower Technol.* **2**, 33 (2004). (in Chinese)

39 Zhang, J. & Zhou, Z. Taiwan province Ming lake pumped storage power station. *Water Conserv. Hydropower Technol.* **3**, 56-58 (1988). (in Chinese)

40 Zhang, S. Castle peak hydropower plant expansion project of computer monitoring and control system design and features. *Hunan Water Resour. Hydropower* **3**, 40-41 (2001). (in Chinese)

41 Zhang, S. Improve the Wenyuhe power station economic benefit analysis of the way. *Shanxi Water Conserv.* **8**, 33-34 (2010). (in Chinese)

42 Zhao, S. *Baiyun Hydropower Scheduling Real-Time Flood Forecasting System Research*. (Hehai University, Nanjing, 2004). (in Chinese with English abstract)

43 Zhao, Y., Qi, Z., Qi, Y., Lv, Q. & Jiao, F. Fallen snow where reservoir irrigation benefit is analysed. *Shandong Province Water Conserv. Sci. Technol.* **2**, 31-33 (1996). (in Chinese)
